# Development and application of a multi-task fusion model using susceptibility-weighted imaging-based substantia nigra and adjacent structures for prognostic prediction of subthalamic nucleus deep brain stimulation in Parkinson’s disease

**DOI:** 10.1093/braincomms/fcag333

**Published:** 2026-09-11

**Authors:** Chi Xiong, Kun Wang, Erkang Cheng, Zhiyong Sun, Bin Cai, Chaoshi Niu, Bo Song

**Affiliations:** Institute of Intelligent Machines, Hefei Institutes of Physical Science, Chinese Academy of Sciences, Hefei 230031, P.R. China; University of Science and Technology of China, Hefei 230026, P.R. China; Department of Neurosurgery, The First Affiliated Hospital of USTC, Division of Life Sciences and Medicine, University of Science and Technology of China, Hefei 230001, P.R. China; Institute of Intelligent Machines, Hefei Institutes of Physical Science, Chinese Academy of Sciences, Hefei 230031, P.R. China; Institute of Intelligent Machines, Hefei Institutes of Physical Science, Chinese Academy of Sciences, Hefei 230031, P.R. China; Institute of Intelligent Machines, Hefei Institutes of Physical Science, Chinese Academy of Sciences, Hefei 230031, P.R. China; Institute of Intelligent Machines, Hefei Institutes of Physical Science, Chinese Academy of Sciences, Hefei 230031, P.R. China; University of Science and Technology of China, Hefei 230026, P.R. China; University of Science and Technology of China, Hefei 230026, P.R. China; Department of Neurosurgery, The First Affiliated Hospital of USTC, Division of Life Sciences and Medicine, University of Science and Technology of China, Hefei 230001, P.R. China; Institute of Intelligent Machines, Hefei Institutes of Physical Science, Chinese Academy of Sciences, Hefei 230031, P.R. China; University of Science and Technology of China, Hefei 230026, P.R. China

**Keywords:** Parkinson’s disease, deep brain stimulation, prognostic prediction, deep learning, susceptibility-weighted imaging

## Abstract

We developed and evaluated a deep learning framework using susceptibility-weighted imaging signatures of the substantia nigra and adjacent structures to predict motor outcomes following subthalamic nucleus deep brain stimulation in Parkinson’s disease. This retrospective study included patients undergoing bilateral subthalamic nucleus deep brain stimulation, and motor response was defined as the percentage improvement in the original Unified Parkinson’s Disease Rating Scale Part III score from the preoperative medication-off condition to the 1-year postoperative stimulation-on/medication-off condition. Patients with at least 50% improvement were classified as optimal responders. A multi-task fusion network integrating prognostic classification and complementary regression learning was developed using preoperative susceptibility-weighted imaging data. In the initial 36-patient cohort, the proposed model outperformed baseline models and showed consistent 5-fold cross-validation performance. Substantia nigra and adjacent structures-centred sub-volumes showed better prognostic performance than non-substantia nigra and adjacent structures regions. In the expanded 60-patient cohort, the model achieved 88.3% accuracy. Additional analyses showed greater percentage improvement in the 39-item Parkinson’s Disease Questionnaire in optimal responders and no prognostic association between swallow-tail sign ratings and motor outcome. These findings suggest that preoperative susceptibility-weighted images covering the substantia nigra and adjacent structures may support prognostic prediction of subthalamic nucleus deep brain stimulation and may provide a practical decision-support tool for preoperative patient selection.

## Teaser

Xiong *et al*. report that susceptibility-weighted MRI of the substantia nigra and adjacent structures, analysed with a multi-task deep learning model, predicts motor outcome after subthalamic nucleus deep brain stimulation in Parkinson’s disease. This imaging-based framework may support preoperative patient selection and prognostic assessment.

## Graphical Abstract

**Graphical Abstract:**
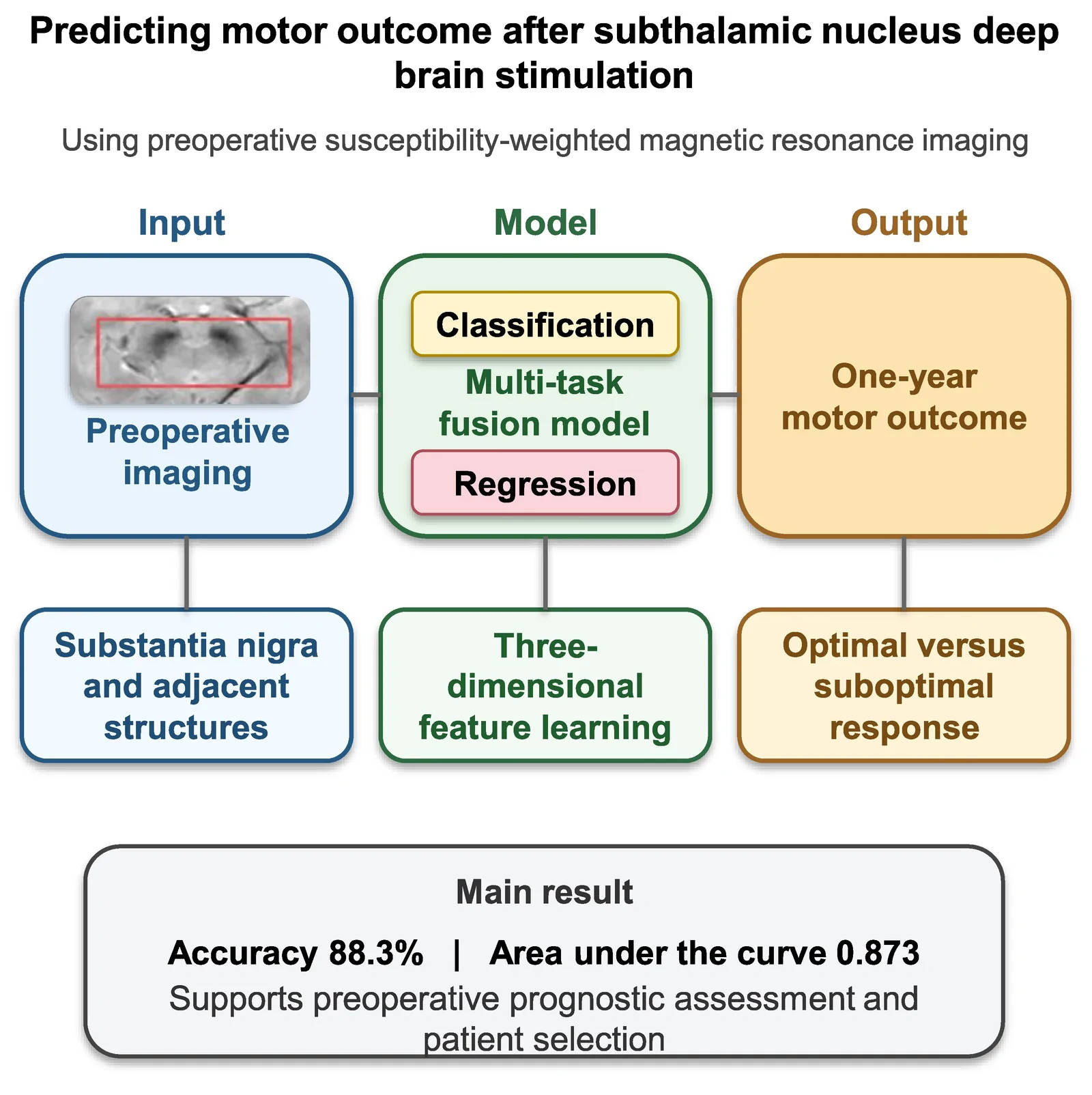

## Introduction

Parkinson’s disease is a prevalent neurodegenerative condition that tends to exacerbate with the progression of its course. While pharmaceutical interventions demonstrate efficacy in the early stages, a significant number of patients necessitate deep brain stimulation (DBS) to alleviate symptoms or mitigate side effects as the disease advances to an advanced stage, characterized by diminished efficacy and potential drug-related complications. DBS has emerged as a pivotal therapeutic intervention, supported by class I evidence, for alleviating motor symptoms associated with Parkinson’s disease. The subthalamic nucleus (STN) is a commonly targeted area for DBS, with its electrical stimulation having been shown to significantly improve motor symptoms in patients with Parkinson’s disease.^1^

Despite its recognized benefits, the efficacy of DBS surgery exhibits significant heterogeneity.^2^ Factors such as implantation accuracy and postoperative management are acknowledged as influential contributors to outcomes. Even in experienced centres, where these factors are meticulously managed, postoperative patients still manifest vastly different outcomes, implying the involvement of undisclosed factors.^3^ Considering the invasiveness and potential risks associated with surgery and anaesthesia, along with potential side effects such as the induction of mental and cognitive symptoms through STN-DBS,^3^ it is important to predict surgical efficacy accurately before operation to help decide whether to perform surgery.

Current expert consensus statements and treatment guidelines for DBS in Parkinson’s disease lack specific indications for efficacy prediction or recommendations regarding suitable prediction methods. Clinically, the widely employed levodopa challenge testing (LCT) serves as a common approach for estimating efficacy.^4^ Beyond its role as a diagnostic tool for Parkinson’s disease, LCT necessitates an improvement rate of ⩾ 30% before surgery is considered advisable. However, the predictive capacity of LCT for postoperative DBS outcome remains controversial, and recent studies have suggested that standard preoperative screening measures, including LCT, have limited ability to predict postoperative motor outcomes.^5,6^ Because the mechanism of DBS in the treatment of Parkinson’s disease differs from that of pharmacological interventions,^7^ it is essential to avoid confusion between the improvement rates, despite both interventions showing the capability to ameliorate patients’ symptoms.

The substantia nigra is pathologically central to Parkinson’s disease, with degeneration of dopaminergic neurons, α-synuclein pathology and abnormal iron accumulation. Susceptibility-weighted imaging (SWI) is sensitive to local magnetic susceptibility changes and can indirectly reflect iron-related alterations in the substantia nigra and nigrosome-1 region. Although the STN is the surgical target of STN-DBS, the substantia nigra and its adjacent structures are closely connected with basal ganglia motor circuits and may contain imaging features related to disease severity, circuit integrity and postoperative motor responsiveness. Therefore, preoperative SWI features covering the substantia nigra and adjacent structures (SNAS) may provide biologically plausible prognostic information for STN-DBS outcome prediction.

Given the inherent limitations of LCT, the pursuit of more accurate methods for preoperative prognostic prediction has become a critical focus of ongoing research in the field. Certain studies have utilized machine learning in combination with medical images to investigate strategies for prognostic prediction^8,9^ or condition evaluation.^10^ In this study, we developed and evaluated a deep learning framework using SWI signatures of the SNAS to predict motor outcomes following STN-DBS in patients with Parkinson’s disease.

## Materials and methods

### Ethical approval

This study was approved by the Ethics Committee of the First Affiliated Hospital of USTC (Approval No.: 2022-RE-154). This retrospective study met the conditions for exemption from informed consent and obtained the informed consent exemption from the Ethics Committee.

### Study design and patients

A single-centre retrospective study was conducted to develop a deep learning model to predict the prognosis of patients with Parkinson’s disease undergoing STN-DBS. The dataset used for model development comprised 60 patients who underwent bilateral STN-DBS in the Department of Neurosurgery of the First Affiliated Hospital of USTC between January 2019 and December 2023. The inclusion criteria were as follows: (i) clinically diagnosed idiopathic Parkinson’s disease based on the 2015 MDS Parkinson’s disease diagnostic criteria^11^; (ii) a disease duration of more than 5 years, with the modified Hoehn–Yahr Scale between 2.5 and 4; (iii) absence of severe cognitive impairment, defined as a Montreal Cognitive Assessment (MoCA) score ≥17 and/or a Mini–Mental State Examination (MMSE) score ≥24 according to the available clinical assessment^12^; (iv) with no obvious mental disorders [Hamilton Anxiety Rating Scale (HAMA) < 29, Hamilton Depression Rating Scale (HAMD) < 21]; (v) no surgical contraindications; (vi) no MRI contraindications and (vii) willingness and ability of the patient and family members to comply with follow-up. Clinical demographic and scale data were extracted from institutional preoperative assessment and postoperative follow-up records.

### Treatment protocol and assessment of treatment response

All eligible patients received bilateral STN-DBS with microelectrode recording assistance. Non-directional Pins L301 electrodes (Pins Medical, China) were used in all patients. The first programming session was performed approximately 1 month after surgery, and subsequent stimulation parameters were adjusted according to clinical symptoms until stable efficacy was achieved.

Postoperative CT images were co-registered with preoperative MRI to evaluate lead location. Active contact coordinates and stimulation-related metrics were retrospectively reconstructed using Lead-DBS. The active contact coordinates were transformed into Montreal Neurological Institute (MNI) space. Stimulation parameters, including voltage, pulse width, frequency and contact configuration, were recorded from the chronic stimulation settings at follow-up. The volume of tissue activated (VTA), electric-field metrics and VTA overlap with the STN and STN functional subregions were calculated for exploratory comparison between optimal and suboptimal responders.

Demographic and clinical data, including age, sex, disease duration, Hoehn–Yahr stage, levodopa equivalent daily dose (LEDD) and LCT responsiveness, were documented preoperatively. The original Unified Parkinson’s Disease Rating Scale (UPDRS), rather than the Movement Disorder Society-sponsored revision of the UPDRS, was used in this cohort. Preoperative UPDRS-III scores were assessed in the medication-off state after withdrawal of dopamine agonists for 72 h and other dopaminergic medications for at least 12 h.

Postoperative motor outcome was assessed at 1 year after surgery in the stimulation-on/medication-off condition after chronic stimulation parameters had been optimized. Clinical assessments were performed by raters blinded to imaging features and model outputs. The UPDRS-III improvement rate was calculated as follows: Improvement (%) = [(preoperative UPDRS-III score−postoperative UPDRS-III score)/preoperative UPDRS-III score] × 100%. Patients with an improvement rate of at least 50% were classified as optimal responders, whereas those with an improvement rate below 50% were classified as suboptimal responders.

### Additional clinical outcome analyses

To evaluate the clinical relevance of the response classification beyond UPDRS-III, additional postoperative clinical outcomes were analysed in an exploratory manner. These included LEDD, UPDRS-IV as an available measure of motor complications, the Non-Motor Symptoms Scale (NMSS), the 39-item Parkinson’s Disease Questionnaire (PDQ-39) as a disease-specific patient-reported quality-of-life measure, MMSE, MoCA, HAMD, HAMA, Parkinson’s Disease Sleep Scale (PDSS) and constipation score. For each scale, preoperative and 1-year postoperative values were summarized, and absolute and percentage changes were calculated where applicable. For scales in which lower scores indicate better clinical status, favourable change was defined as the preoperative score minus the postoperative score; for scales in which higher scores indicate better status, favourable change was defined as the postoperative score minus the preoperative score.

### Swallow-tail sign analysis

A retrospective swallow-tail sign analysis was performed on preoperative SWI images. The left and right swallow-tail signs were rated by two radiologists who were blinded to postoperative motor outcomes and model predictions, and final ratings were determined by consensus. Left, right and total swallow-tail scores were compared between optimal and suboptimal responders. Spearman correlation analyses were performed between swallow-tail scores and continuous UPDRS-III improvement rates. Receiver operating characteristic curve (ROC) analyses were used to evaluate the prognostic performance of swallow-tail scores for differentiating optimal from suboptimal responders.

### Data acquisition and preprocessing

All patients underwent preoperative 3.0-T MRI scanning (GE MR750, USA) with a 32-channel head coil. SWI was acquired using a gradient-echo sequence with the following parameters: 104 slices; field of view, 240 mm; phase field of view, 100%; repetition time/echo time, 39/23.5 ms; flip angle, 10°; sampling bandwidth, 162.73 Hz/pixel and acquisition time, 4 min 13 s.

To reduce the influence of irrelevant brain tissues, three dimensional (3D) sub-regions within the SWI images covering the SNAS were extracted for prognostic prediction. The SNAS-centred sub-volume was designed to include the substantia nigra and adjacent deep brain structures, including part of the STN region. A representative example of SNAS-centred sub-volume extraction in axial, coronal and sagittal views is shown in Supplementary Fig. 2. These 3D subregions were meticulously extracted by clinical surgeons from the First Affiliated Hospital of USTC as follows.

First, clinical surgeons examined the 3D SWI image slice by slice to identify the boundaries of the substantia nigra. Subsequently, a fixed-size 3D sub-volume encompassing both the left and right substantia nigra was extracted. The dimensions of this 3D sub-volume were set to 10 × 40 × 80 voxels. Finally, each 3D sub-volume was normalized by subtracting its mean intensity and dividing by its standard deviation to facilitate network training and improve convergence stability.


$$f(z,y,x)=\{\begin{array}{c} \frac{V_{z,y,x}-mean(V_{z,y,x})}{std(V_{z,y,x})},\;\;\;\;\;\;\;\;\;\;\;\;\;V_{z,y,x}>0\\ 0\;\;\;\;\;\;\;\;\;\;\;\;\;\;\;\;\;\;\;\;\;\;\;\;\;\;\;\;\;\;\;\;\;\;\;\;\;\;\;\;\;\;V_{z,y,x}=0 \end{array}$$

### Model development

**Figure 1:**
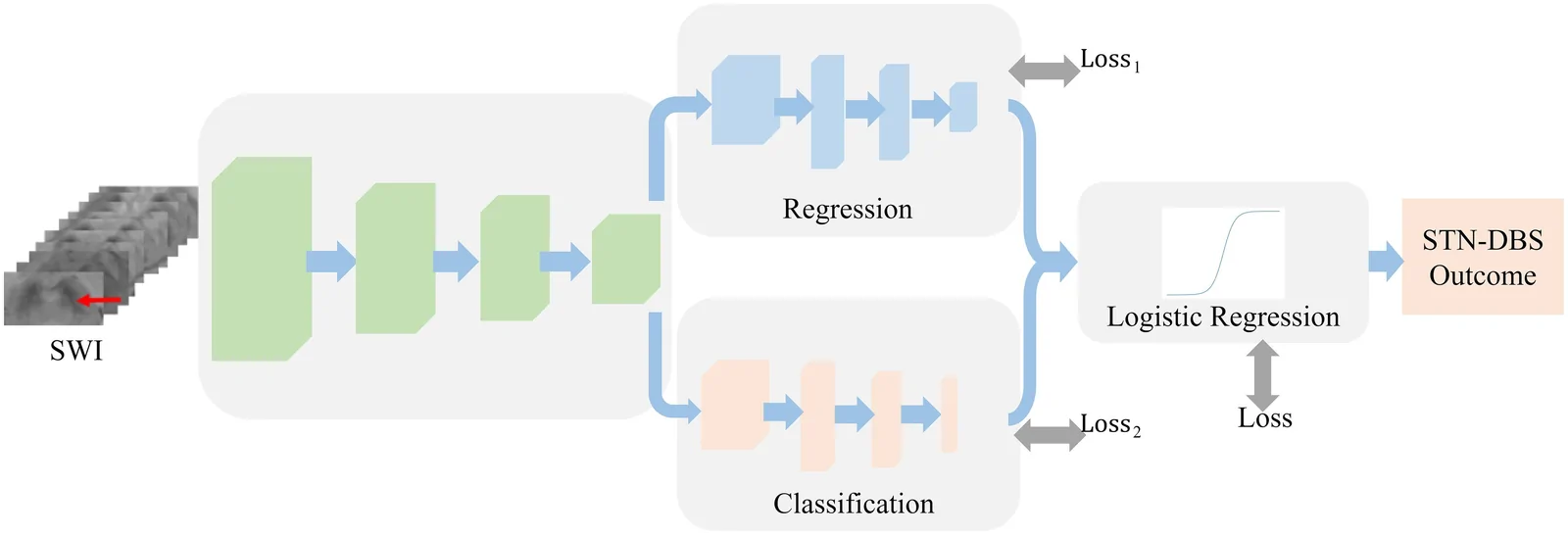
**Multi-task fusion framework.** Overview of the proposed framework for prognostic prediction of subthalamic nucleus deep brain stimulation (STN-DBS) motor outcome in Parkinson’s disease. A preoperative susceptibility-weighted imaging (SWI) sub-volume covering the substantia nigra and adjacent structures (SNAS) is first processed by a three-dimensional feature-learning backbone. The extracted features are then fed into a regression branch and a classification branch. The outputs of the two branches are fused by a logistic regression classifier to generate the final prognostic prediction. The arrow indicates the substantia nigra.

In this section, we describe the proposed multi-task fusion framework for prognostic prediction of STN-DBS motor outcome in Parkinson’s disease. The overall architecture is shown in Fig. 1. The framework consists of four components: a backbone for 3D feature learning, a classification branch, a regression branch and a final logistic regression classifier for fusion. The classification branch estimates the probability of optimal or suboptimal DBS motor response, whereas the regression branch predicts the continuous UPDRS-III improvement rate as a complementary task. The final classifier fuses the outputs of the classification and regression branches to generate the final prognostic prediction.

We formulated prognostic prediction as a binary classification task. To improve the representation ability of the neural network and to make use of the continuous clinical outcome information, complementary-task learning was introduced. Specifically, the regression branch was used as an auxiliary task to learn the continuous motor improvement rate and to support the primary classification task.

The backbone was used for 3D feature learning. To leverage the anatomical information contained in the SNAS-centred 3D SWI sub-volume, 3D convolutional blocks were used to construct the backbone. Each 3D convolutional block consisted of 3D convolution, InstanceNorm and LeakyReLU activation, followed by max pooling. After three down-sampling operations, the original 3D sub-volume size was reduced from 10 × 40 × 80 to 5 × 5 × 10. The output feature maps of the backbone served as the input for both the classification and regression branches.

**Figure 2:**
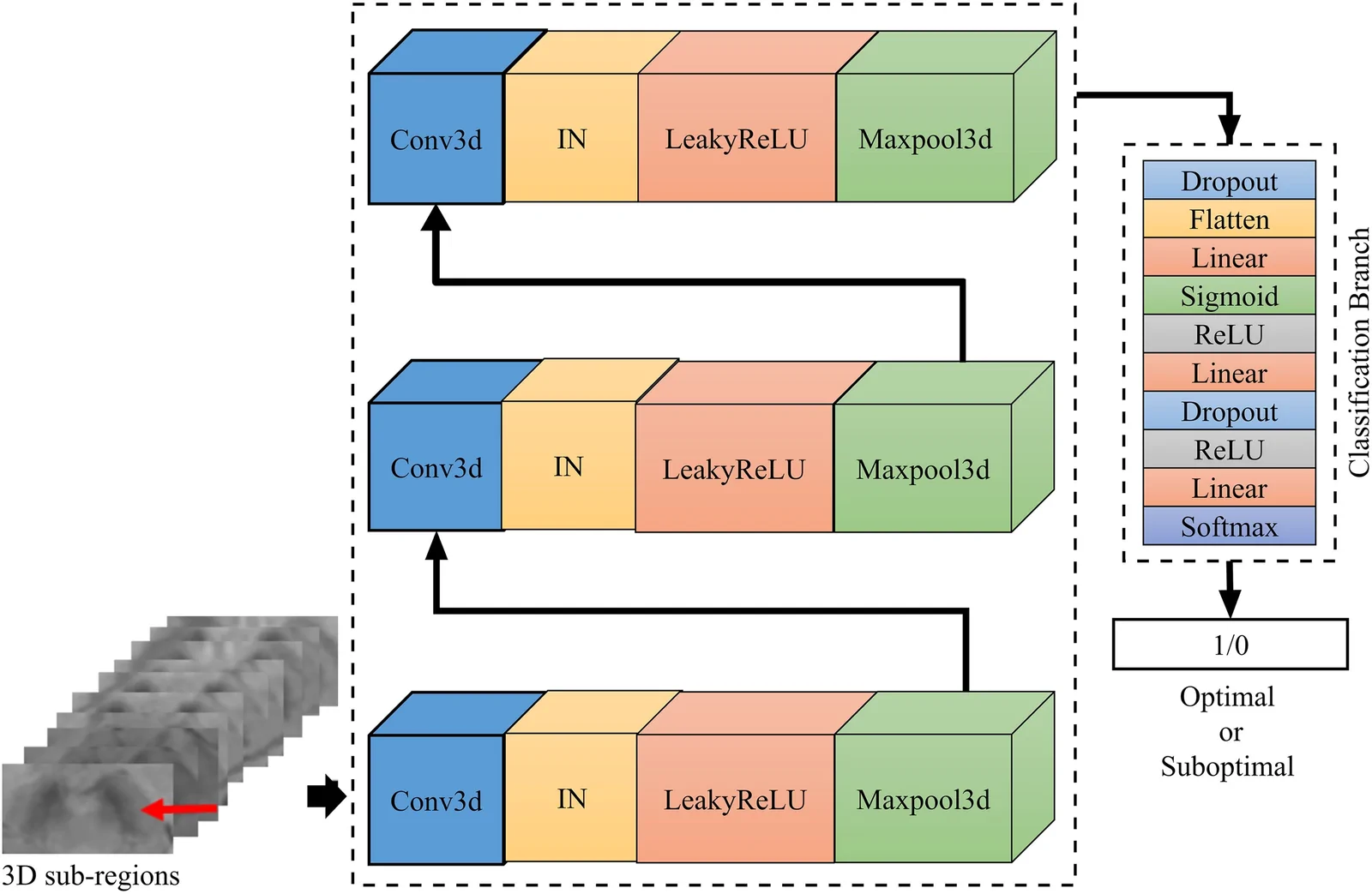
**Classification pathway.** Architecture of the classification pathway. The input substantia nigra and adjacent structures-centred three-dimensional susceptibility-weighted imaging sub-volume is processed by three consecutive three-dimensional convolutional blocks, each consisting of Conv3d, InstanceNorm, LeakyReLU and MaxPool3d. The resulting feature representation is then passed through the classification head composed of dropout, flattening and three linear layers. The first two linear layers are followed by rectified linear unit activation, and the final linear layer is followed by a softmax operation to generate class probabilities. The arrow indicates the substantia nigra. 3D, three dimensional; Conv3d, three-dimensional convolution; IN, instance normalization; LeakyReLU, leaky rectified linear unit; Maxpool3d, three-dimensional max pooling; ReLU, rectified linear unit.

The classification branch was designed to classify DBS motor outcome into optimal or suboptimal response, as shown in Fig. 2. This branch consisted of three linear layers. The first two linear layers were followed by rectified linear unit activation, and the final linear layer was followed by a softmax operation to generate class probabilities.

The regression branch was designed to predict the continuous UPDRS-III improvement rate. For regression training, the UPDRS-III improvement rate was represented as a normalized proportion ranging from 0 to 1. This branch also consisted of three linear layers. The first two linear layers were followed by rectified linear unit activation, and the final linear layer was followed by a sigmoid operation to generate an output within the same 0–1 range.

**Figure 3:**
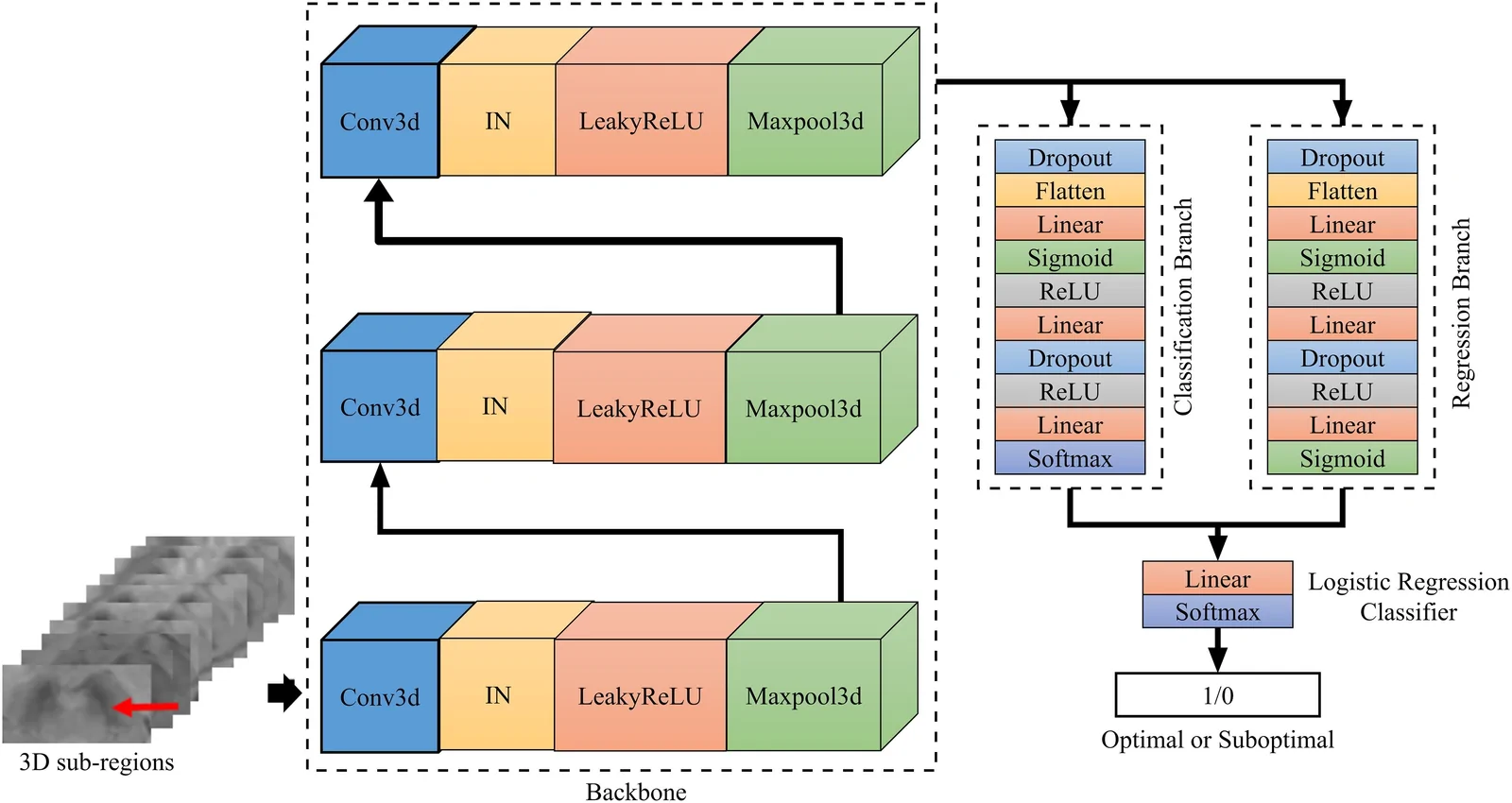
**Multi-task fusion architecture.** Architecture of the proposed multi-task fusion framework. The input substantia nigra and adjacent structures-centred three-dimensional susceptibility-weighted imaging sub-volume is first processed by a shared three-dimensional convolutional backbone. The learned features are then fed into two parallel branches: a classification branch for predicting optimal versus suboptimal motor outcome and a regression branch for predicting the continuous UPDRS Part III improvement rate. The outputs of the two branches are finally fused by a logistic regression classifier to generate the final prognostic prediction. The arrow indicates the substantia nigra. 3D, three dimensional; Conv3d, three-dimensional convolution; IN, instance normalization; LeakyReLU, leaky rectified linear unit; Maxpool3d, three-dimensional max pooling; ReLU, rectified linear unit.

The complete multi-task fusion framework, including the backbone, classification branch, regression branch, and fusion classifier, is shown in Fig. 3. In the fusion stage, a logistic regression classifier took the outputs of both the classification branch and the regression branch as input and produced the final prognostic prediction. In this way, the model incorporated both categorical response information and continuous motor improvement information during training. The loss function contained three optimization objectives: the classification loss from the classification branch, the regression loss from the regression branch and the logistic regression loss from the final fusion classifier. For case *i* in the classification branch, with probability $p_{i}$ and ground truth class $y_{i}$, the Cross Entropy loss is employed for the classification loss $L_{cls}$ and the logistic regression loss $L_{log}$. The SmoothL1 loss is utilized as the regression loss $L_{reg}$ for regressing the improvement score. The final loss $L_{total}$ is defined as:


$$L_{log}=L_{cls}=\frac{1}{N}\underset{i}{\sum }\;L_{i}=\frac{1}{N}\underset{i}{\sum }-[y_{i}\cdot \log\;(\;p_{i})+(1-y_{i})\cdot \log(1-p_{i})]$$

$$L_{reg}=\{\begin{array}{c} 0.5\cdot {\left(y_{i}-p_{i}\right)}^{2}\;\;\;\;\;\;\;if\;|y_{i}-p_{i}|<1\\ |y_{i}-p_{i}|-0.5\;\;\;\;\;\;\;\;\;\;\;\;\;otherwise\;\; \end{array}$$

$$L_{total}=\alpha \cdot L_{log}+\beta \cdot L_{cls}+\gamma \cdot L_{reg}$$

where α, β and γ are weighting parameters for the logistic regression loss, classification loss and regression loss, respectively.

### Implementation details and performance evaluation

A 5-fold cross-validation strategy was used to evaluate model performance. In each fold, the dataset was split at the patient level into training and testing subsets with a ratio of 4:1, ensuring that images from the same patient did not appear in both training and testing subsets. Data augmentation was applied only to the training data in an on-the-fly manner, including random elastic deformation, random rotation, random scaling and random mirroring. The network was optimized using the Adam optimizer with an initial learning rate of 0.01 and a weight decay of 1 × 10^−4^.

Because the proposed framework used a multi-task learning strategy, the classification and regression tasks could mutually reinforce but also compete with each other during optimization. To reduce potential task imbalance, the weighting parameters α, β and γ were dynamically adjusted during training.

**Figure 4:**
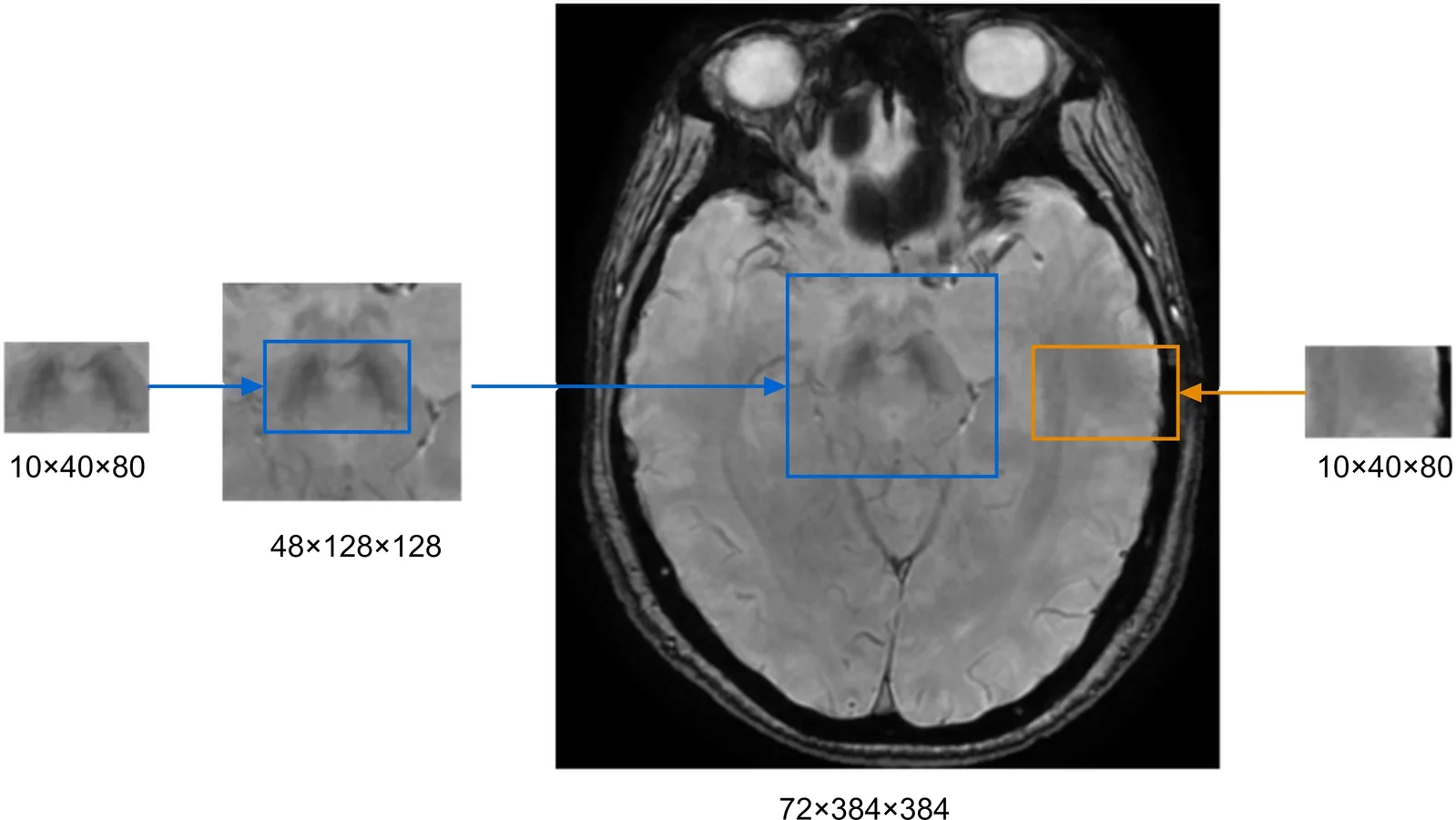
**Anatomical specificity analysis.** Illustration of the three-dimensional sub-regions extracted from preoperative susceptibility-weighted imaging for anatomical specificity analysis. The outlined boxes indicate substantia nigra and adjacent structures-centred sub-regions used for model training at different spatial scales and a control sub-region extracted from a non-substantia nigra and adjacent structures location. The substantia nigra and adjacent structures-centred region covered the substantia nigra and adjacent deep brain structures, including part of the subthalamic nucleus region. These experiments were used to examine whether prognostic information was concentrated in the substantia nigra and adjacent structures. Abbreviation: SNAS, substantia nigra and adjacent structures.

To evaluate data scalability within this single-centre retrospective cohort, we first trained and tested the model using 36 cases collected from January 2019 to December 2021. Subsequently, the dataset was expanded to 60 cases by adding 24 patients collected from January 2022 to December 2023, and the same 5-fold cross-validation strategy was applied to the expanded cohort. Because no independent external cohort was available, the expanded 60-patient analysis should be interpreted as an internal scalability analysis rather than external validation. To further examine whether SNAS-centred SWI sub-regions contained prognostic information, additional experiments were performed using 3D sub-regions of different sizes and locations, as shown in Fig. 4. These included SNAS-centred sub-volumes with different spatial extents and control sub-volumes extracted from non-SNAS regions. All sub-regions were assigned the same patient-level outcome labels, and the network was retrained using the same training and evaluation strategy.

The prognostic prediction performance was evaluated using accuracy, precision, recall, F1 score and area under the receiver operating characteristic curve (AUC). Accuracy was defined as the proportion of correctly classified samples. Precision was defined as the proportion of true positives among samples predicted as positive. Recall was defined as the proportion of true positives among all actual positive samples. The F1 score was calculated as the harmonic mean of precision and recall. AUC was used to evaluate the discrimination ability of the binary classification model. $Accuracy=\frac{TP+TN}{TP+FP+TN+FN}$


$$Precision=\frac{TP}{TP+FP}$$

$$Recall=\frac{TP}{TP+FN}$$

$$F1=\frac{2\times Precision\times Recall}{Precision+Recall}$$

where TP, TN, FP and FN denote true positives, true negatives, false positives and false negatives, respectively.

### Baseline model

Because the primary prognostic prediction task was formulated as a binary classification problem, a 3D ResNet50 model was used as the baseline classification model. The same SNAS-centred 3D SWI sub-volumes were used as input for the baseline model. The baseline model was trained and evaluated using the same patient-level data splitting strategy, 5-fold cross-validation procedure, data augmentation strategy and performance metrics as the proposed multi-task fusion framework. The architecture of the 3D ResNet50 baseline model is shown in Supplementary Fig. 1.

### Statistical analysis

Continuous variables were summarized as mean ± standard deviation or median with interquartile range, and categorical variables were summarized as counts and percentages. Between-group comparisons of continuous clinical variables between optimal and suboptimal responders were performed using the Mann–Whitney U-test because of the modest sample size and potential non-normal distribution of several clinical scales. Categorical variables were compared using Fisher’s exact test or the chi-square test, as appropriate. Rank-biserial correlation was reported as an effect-size measure for Mann–Whitney U-tests.

For additional clinical outcome analyses, preoperative values, postoperative values, absolute changes, favourable changes and percentage favourable changes were compared between groups. Spearman correlation was used to assess associations between swallow-tail scores and continuous UPDRS-III improvement rates. ROC analysis was used to evaluate the ability of swallow-tail scores to discriminate optimal from suboptimal responders. Benjamini–Hochberg false discovery rate correction was applied for exploratory multiple comparisons. DBS stimulation parameters, active contact coordinates, VTA metrics and VTA-STN overlap variables were compared between groups using Mann–Whitney U-tests and were interpreted as exploratory analyses. A two-sided *P* < 0.05 was considered nominally significant. Statistical analyses were performed using Python, including pandas, scipy and scikit-learn.

## Results

### Patient characteristics

A total of 60 patients were included, all of whom underwent successful surgery. Postoperative CT-MRI fusion was performed for routine assessment of lead location, and Lead-DBS-based reconstruction was available for exploratory electrode localization and stimulation-related analyses. Core demographic and baseline clinical characteristics are presented in Table 1, and detailed baseline clinical variables are provided in Supplementary Table 1. The optimal responder group included 37 patients, and the suboptimal responder group included 23 patients. Baseline demographic and clinical characteristics were generally comparable between groups, including age, sex, disease duration, age at onset, preoperative Hoehn–Yahr stage, LEDD, preoperative UPDRS-III, UPDRS-IV, LCT responsiveness, NMSS, PDQ-39, MMSE, MoCA, HAMD, HAMA, PDSS and constipation score, with no nominally significant between-group differences.

**Table 1:** Demographic and clinical characteristics

Variable	Optimal group	Suboptimal group	*P*-value
***N***	37	23	
**Age, years**	56.19 ± 6.51	60.00 ± 6.66	0.072
**Sex, M/F**	22/15	11/12	0.431
**Disease duration, years**	8.38 ± 2.91	8.91 ± 3.92	0.878
Hoehn**–**Yahr Scale, median **(**IQR**)**	3 (3, 4)	3 (3, 4)	0.545
**LEDD, mg**	714.86 ± 383.90	646.20 ± 241.43	0.825
**LCT response**	0.57 ± 0.13	0.52 ± 0.14	0.146
**UPDRS-III**	54.38 ± 12.67	51.78 ± 10.86	0.420

LCT, levodopa challenge test; LEDD, levodopa equivalent daily dose. UPDRS-III, Unified Parkinson’s Disease Rating Scale Part III; *N*, number of patients.

### Additional clinical outcomes beyond UPDRS-III

Additional postoperative clinical outcomes are summarized in Supplementary Table 2. Optimal responders showed greater improvement in quality of life as measured by PDQ-39. Postoperative PDQ-39 scores were lower in optimal responders than in suboptimal responders (37.95 ± 22.30 versus 56.13 ± 20.30, nominal *P* = 0.005). The percentage favourable change in PDQ-39 was also greater in optimal responders (39.19 ± 31.09% versus 13.75 ± 28.05%, nominal *P* = 0.003) and remained significant after false discovery rate correction (*q* = 0.031). The absolute favourable change in PDQ-39 was nominally greater in optimal responders (24.35 ± 21.58 versus 10.96 ± 19.05, nominal *P* = 0.011), although it did not remain significant after false discovery rate correction (*q* = 0.106). Changes in LEDD, UPDRS-IV, NMSS, MMSE, MoCA, HAMD, HAMA, PDSS and constipation score did not significantly differ between groups.

### Individual UPDRS-III values and continuous improvement rates

Individual preoperative UPDRS-III scores, 1-year postoperative stimulation-on/medication-off UPDRS-III scores, continuous UPDRS-III improvement rates and binary response classifications are provided in Supplementary Table 3. The continuous UPDRS-III improvement rate was used by the regression branch of the proposed multi-task framework as complementary learning information, whereas the binary responder label was used for the primary prognostic classification task.

### Swallow-tail sign analysis

Swallow-tail sign analyses are summarized in Supplementary Table 4. Left, right and total swallow-tail scores did not significantly differ between optimal and suboptimal responders. The total swallow-tail score was 1.81 ± 0.52 in optimal responders and 1.74 ± 0.54 in suboptimal responders (*P* = 0.453). Swallow-tail scores were not significantly correlated with continuous UPDRS-III improvement rates (total score: Spearman’s rho = 0.030, *P* = 0.817). The prognostic performance of swallow-tail scores was close to chance level, with AUC values of 0.503 for the left score, 0.533 for the right score and 0.538 for the total score.

### DBS electrode localization and stimulation-related analyses

All patients were implanted with bilateral non-directional Pins L301 electrodes. Postoperative CT-MRI fusion and Lead-DBS-based reconstruction were used to evaluate active contact coordinates and stimulation-related metrics. A group-level Lead-DBS reconstruction of bilateral electrode trajectories and active contacts relative to the STN region is shown in Supplementary Fig. 3. Active contact coordinates in MNI space are summarized in Supplementary Table 5. No significant between-group differences were observed in the left or right active contact coordinates along the x, y or z axes.

Stimulation parameters and VTA-related metrics are summarized in Supplementary Table 6, and patient-level DBS stimulation parameters, active contact coordinates, VTA-related metrics and VTA-STN overlap values are provided in Supplementary Table 7. The total bilateral total electrical energy delivered was 114.43 ± 69.23 μJ/s in optimal responders and 133.58 ± 65.74 μJ/s in suboptimal responders, with no significant between-group difference (*P* = 0.176). The mean bilateral VTA volume was numerically larger in suboptimal responders than in optimal responders (110.75 ± 50.31 versus 83.70 ± 43.94 mm^3^; nominal *P* = 0.040), but this finding did not remain significant after false discovery rate correction.

Exploratory VTA-STN overlap analyses showed that the total bilateral VTA-STN overlap was numerically larger in suboptimal responders than in optimal responders (190.69 ± 88.32 versus 144.28 ± 72.84 mm^3^; nominal *P* = 0.046). The total bilateral VTA overlap with the motor STN subregion was also larger in suboptimal responders (46.65 ± 21.97 versus 32.49 ± 20.65 mm^3^; nominal *P* = 0.015). However, none of these VTA-related differences remained significant after false discovery rate correction. These exploratory findings suggest that stimulation-related factors may also contribute to postoperative motor outcome and should be incorporated into future multimodal prediction models.

### Prediction performance comparison of different models

We first evaluated prognostic prediction performance using the initial 36-patient cohort collected from January 2019 to December 2021. A 3D ResNet50 model was used as the baseline classification model and was trained and tested using the same SNAS-centred inputs and 5-fold cross-validation strategy. As shown in Table 2, the proposed multi-task fusion strategy outperformed the 3D ResNet50 baseline and the single-task branches in terms of accuracy, recall, F1 score and AUC. Compared with the classification-only branch, the fused model achieved higher overall performance, suggesting that complementary regression learning using the continuous UPDRS-III improvement rate contributed additional prognostic information. The 5-fold cross-validation results are shown in Table 3.

**Table 2:** The performance with different prediction strategy

Method	Accuracy	Precision	Recall	F1	AUC
**3D ResNet50**	0.575	0.68	0.647	0.653	0.417
**Classification**	0.825	0.843	0.92	0.871	0.8
**Regression**	0.7	0.9	0.607	0.709	0.817
**Fused**	0.85	0.867	0.92	0.887	0.807

Classification, only using the classification branch; Regression, only using the regression branch; Fused, using the multi-task fusion strategy.

**Table 3:** Five-fold cross-validation for the performance of the prognostic prediction using multi-task fusion strategy

Fold	Accuracy	Precision	Recall	F1	AUC
**1**	0.75	0.75	1.0	0.857	0.5
**2**	1.0	1.0	1.0	1.0	1.0
**3**	1.0	1.0	1.0	1.0	1.0
**4**	0.625	0.75	0.6	0.667	0.8
**5**	0.875	0.833	1.0	0.909	0.733
**Average**	0.85	0.867	0.92	0.887	0.807

We additionally assessed the multi-task prediction framework using different loss components. As shown in Table 4, compared with using only the final logistic regression loss, the multi-task loss achieved improved performance, with absolute improvements in accuracy (0.850 versus 0.775), recall (0.920 versus 0.773) and F1 score (0.887 versus 0.817).

**Table 4:** The performance of the multi-task prediction framework using different loss components

Loss	Accuracy	Precision	Recall	F1	AUC
$L_{log}$	0.775	0.883	0.773	0.817	0.847
$L_{log}+L_{cls}+L_{reg}$	0.85	0.867	0.92	0.887	0.807

### Prognostic specificity of SNAS SWI features

To examine the anatomical specificity of the SWI-based prognostic features, we compared SNAS-centred sub-volumes of different sizes with control sub-volumes extracted from non-SNAS regions. As shown in Table 5, the SNAS-centred 10 × 40 × 80 sub-volume achieved better prognostic performance than larger sub-volumes and non-SNAS control regions. In contrast, non-SNAS regions showed substantially lower AUC values, suggesting that prognostically relevant information appeared to be concentrated around the SNAS rather than being driven by non-specific whole-image features.

**Table 5:** The prediction performance using the 3D sub-regions with different size and different location

Region-size (pixel)	Method	Accuracy	Precision	Recall	F1	AUC
10 × 40 × 80	Fused	0.85	0.867	0.92	0.887	0.807
	3D ResNet50	0.575	0.68	0.647	0.653	0.417
48 × 128 × 128	Fused	0.725	0.725	1.0	0.833	0.673
	3D ResNet50	0.625	0.808	0.64	0.642	0.483
72 × 384 × 384	Fused	0.65	0.65	1.0	0.787	0.567
	3D ResNet50	0.625	0.693	0.813	0.734	0.6
**Non-SNAS regions**	Fused	0.625	0.643	0.967	0.769	0.373
	3D ResNet50	0.65	0.671	0.88	0.757	0.613

### Internal scalability analysis of the multi-task prognostic model

To evaluate the internal scalability of the prediction network, the dataset was expanded to 60 patients by adding 24 cases collected from January 2022 to December 2023. The same 5-fold patient-level cross-validation strategy was applied to the expanded cohort. As detailed in Table 6, the multi-task fusion architecture achieved a mean accuracy of 88.3% and a mean AUC of 0.873 in the expanded cohort. Because this analysis was performed within a single-centre retrospective dataset, it should be interpreted as an internal scalability analysis rather than external validation.

**Table 6:** Five-fold cross-validation for the performance of the prognostic prediction using multi-task fusion strategy with 60 cases

Fold	Accuracy	Precision	Recall	F1	AUC
**1**	0.666667	0.75	0.75	0.75	0.625
**2**	0.916667	0.875	1.0	0.933333	0.9
**3**	0.916667	1.0	0.875	0.933333	0.9375
**4**	0.916667	0.875	1.0	0.933333	0.9
**5**	1.0	1.0	1.0	1.0	1.0
**Average**	0.883333	0.9	0.925	0.91	0.8725

**Figure 5:**
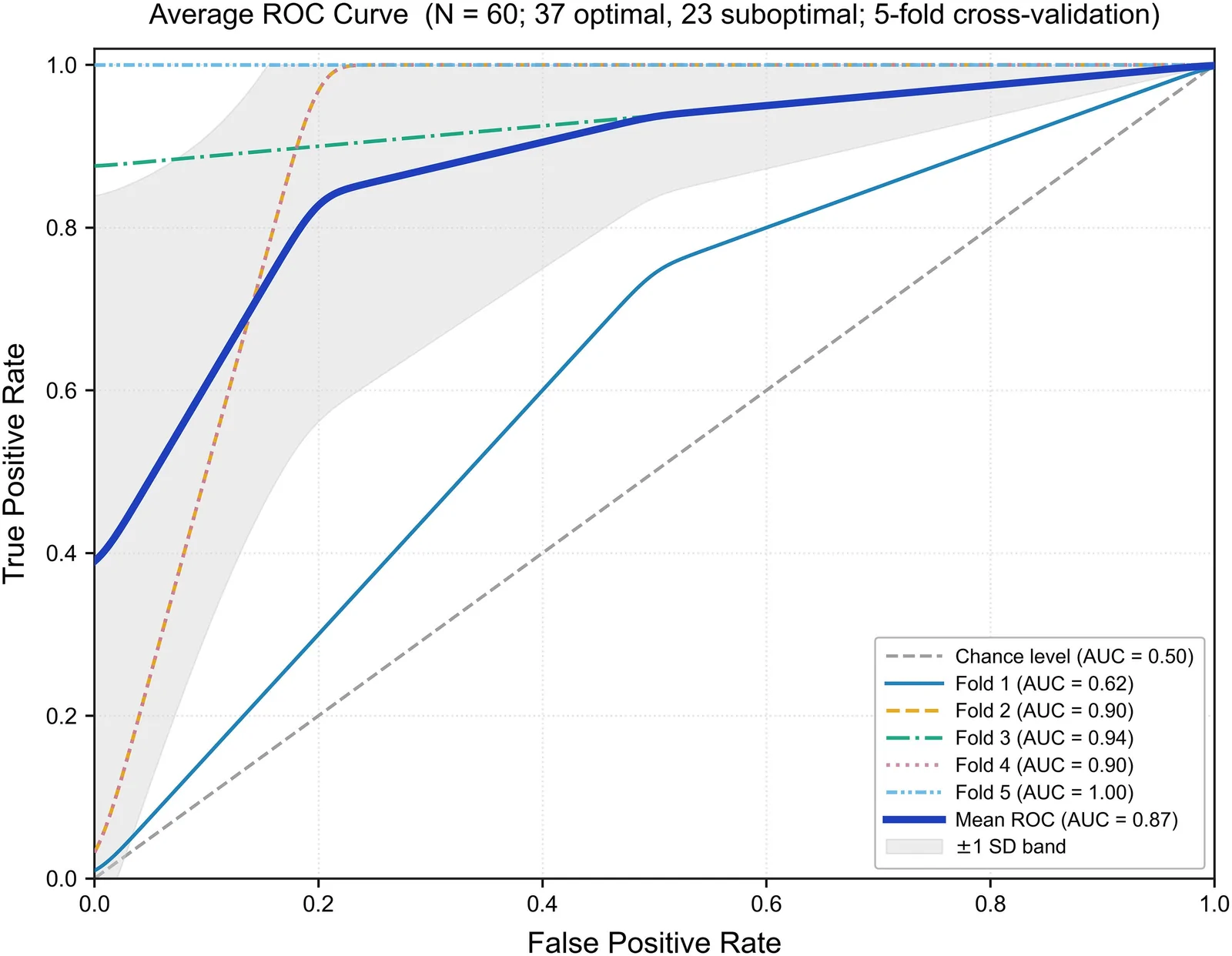
**Receiver operating characteristic curves.** Receiver operating characteristic curves of the 5-fold cross-validation in the expanded 60-patient cohort (*N* = 60). Lines with different line styles represent the receiver operating characteristic curves of individual folds, and the thick solid line represents the mean receiver operating characteristic curve. The shaded band represents ±1 standard deviation across folds, and the dashed diagonal represents chance-level performance. AUC, area under the receiver operating characteristic curve.

The ROC curves of the 5-fold cross-validation in the expanded 60-patient cohort are shown in Fig. 5.

**Figure 6:**
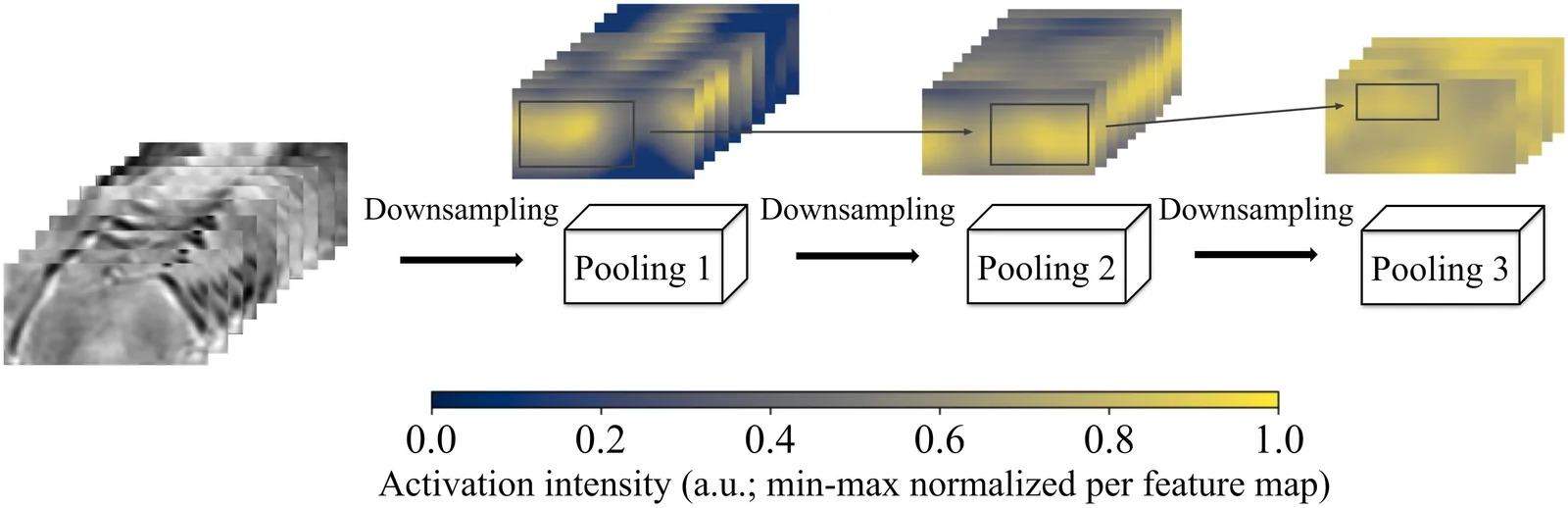
**Feature map visualization.** Visualization of feature maps before each pooling operation in the backbone. Activation intensity was min–max normalized separately for each feature map and is displayed in arbitrary units (a.u.) on a scale from 0 to 1, with higher values indicating stronger responses. The feature maps show prominent responses around the substantia nigra and adjacent structures (SNAS), suggesting that the model primarily extracted prognostically relevant information from the SNAS-centred region.

Furthermore, feature maps before each pooling operation in the backbone are displayed in Fig. 6. The heatmaps showed that the feature extraction process primarily emphasized the region where the substantia nigra is located, supporting that the features used for prognostic prediction were mainly derived from the SNAS-centred sub-volume.

## Discussion

Even though serving as a neuroregulatory therapy with significant potential benefits for patients,^13^ DBS should be guided by stringent patient selection in clinical practice,^14^ owing to its invasiveness and heterogeneous efficacy. With increasing experience and enhanced understanding, these selection criteria have become more stringent,^15^ incorporating considerations such as neuropsychology, cognitive ability and genetic characteristics,^16,17^ with the aim of achieving favourable outcomes for patients. Accurate preoperative prediction of DBS efficacy could better support treatment decision-making, and this has been a major research focus in the field. However, the method of using LCT as a traditional clinical assessment to predict prognosis has certain limitations.

Therefore, a number of studies have explored alternative methods for prognosis prediction, including those based on clinical symptoms,^18^ nucleus electrophysiological signals,^19^ EMG signals,^20^ EEG,^21^ functional fMRI data,^22^ the cortical thickness of the left lateral occipital region,^23^ as well as prognosis prediction through radiomics using MRI data.^24^ Radiomics is a method for extracting subtle quantitative features from medical images for clinical applications and has been applied to the diagnosis of Parkinson’s disease^25^ and prognostic prediction by leveraging machine learning algorithms.^18,24,26-29^ Liu *et al*.^24^ employed imaging genomics in conjunction with machine learning methods, focusing on fine features of the substantia nigra and employing binary logistic regression analysis. This innovative approach demonstrates the ability to predict the prognosis outcomes of STN-DBS in improving motor symptoms of Parkinson’s disease with an accuracy of 82%, a significant improvement compared to the currently clinically used LCT. Tsai *et al*.^28^ employed a stepwise multivariate regression model, utilizing 13 handcrafted features from the preoperative diffusion tensor imaging of patients with Parkinson’s disease, to accurately predict clinical outcomes 2 years after surgery. Saudargiene *et al*.^27^ similarly employed imaging genomics in conjunction with machine learning, focusing on radiological feature analysis of the amygdala-hippocampus. Their findings suggest that changes in imaging genomics reveal the migration and deposition of pathological α-synuclein protein. A decrease in α-synuclein in the amygdala indicates advanced Parkinson’s disease and a poorer prognosis for DBS treatment.

We hypothesized that alterations in the SNAS may correlate with disease status and DBS prognosis in patients with Parkinson’s disease, and that such alterations could be reflected on SWI images. In this study, the results showed that SNAS-centred SWI images could be used to predict motor improvement after DBS surgery, which is supported by previous studies. The pathology of Parkinson’s disease is characterized by the depletion of dopamine neurons and the aggregation of alpha-synuclein in the substantia nigra.^30^ Numerous studies have demonstrated that iron deposition in the substantia nigra is a contributory factor in Parkinson’s disease, while SWI can reflect the deposition well. Ariz *et al*. have proved that quantification of neuromelanin and iron in Nigrosome-1 could emerge as a potential biomarker for Parkinson’s disease by SWI image.^31^ Additionally, the degree of iron deposition exhibits a positive correlation with the disease’s progression,^32^ while the progression of iron deposition is also associated with different subtypes of Parkinson’s disease.^33^ Although the STN represents a crucial node within the motor circuit and is commonly targeted for DBS, it is not the primary pathological nucleus in Parkinson’s disease.^34^ Given that the substantia nigra is closely related to basal ganglia motor circuits and adjacent deep brain structures, we used SWI features of the SNAS to predict the efficacy of STN-DBS, which was consistent with our hypothesis. Our study results indicate that the prediction performance using SNAS-centred regions was higher than that using non-SNAS regions, suggesting that prognostically relevant information may be enriched in the SNAS. In addition, the prediction accuracy decreases when the extended substantia nigra region is used, which may be due to background interference.

In this study, patients were categorized into optimal and suboptimal groups based on whether postoperative motor symptoms improved by more than 50%. We acknowledge that dichotomizing a continuous motor improvement measure may lead to loss of information. To partly address this issue, individual continuous UPDRS-III improvement rates are now provided, and the regression branch of the proposed multi-task framework incorporated the continuous improvement rate as complementary learning information. Nevertheless, responder classification was retained as the primary endpoint because it provides an intuitive and clinically interpretable threshold for identifying patients with suboptimal motor benefit after STN-DBS. Although most studies have set the threshold at ≥30% with reference to the LCT,^24,35^ improvement of <50% in the total motor score often results in patient dissatisfaction. Moreover, improvement exceeding 30% was observed in the majority of patients in clinical practice. Hence, we defined the demarcation threshold as 50%, which has also been adopted in previous studies.^36^ A comparative analysis of preoperative baseline data revealed no significant between-group differences in core demographic and clinical characteristics, including age, sex, disease duration, Hoehn–Yahr stage, LEDD, medication-off UPDRS-III score and LCT improvement rate, indicating that the two groups were statistically comparable. Notably, there was no significant difference in the LCT improvement rate, consistent with previous studies suggesting that LCT alone may not optimally predict motor outcome after DBS.^5,6^ Quality of life has also been reported to be associated with DBS outcome, further supporting the clinical relevance of preoperative prognostic assessment.^37^

In contrast to previous studies that relied on handcrafted feature design followed by conventional machine learning models such as random forest,^18,24,26,29^ our approach directly used SWI images as model input in an end-to-end manner. This avoided manual feature engineering and allowed the network to learn latent prognostic features directly from the SNAS. Adams *et al*.^38^ developed a convolutional neural network using DaTscan SPECT images obtained at baseline and year 1 to regress UPDRS-III scores at year 4. In contrast, we formulated prognostic prediction primarily as a classification task and introduced a multi-task fusion strategy in which the regression branch served as a complementary task to improve classification performance. We also evaluated a regression-only strategy using SWI images, and the results are shown in Table 2. As demonstrated in Table 2, the proposed multi-task fusion strategy outperformed the regression-only model. We developed a deep learning-based multi-task fusion framework for SWI-based prognostic prediction of STN-DBS motor outcome. By incorporating complementary-task learning, the model achieved an accuracy of 88.3% and an AUC of 0.873. These findings suggest that subtle differences in SNAS-centred SWI images may serve as promising imaging biomarkers related to DBS prognosis in Parkinson’s disease and that the proposed model may provide neurosurgeons with a practical decision-support tool for identifying patients who are more likely to benefit from surgery. Although feature map visualization suggested that the network mainly focused on the SNAS region, the proposed deep learning framework remains less transparent than conventional statistical or radiomics-based models. Therefore, the current visualization results should be regarded as a preliminary interpretability analysis rather than a complete explanation of the model decision-making process. Future studies should incorporate more advanced explainable artificial intelligence methods to improve model transparency and clinical interpretability.

We also performed exploratory analyses of DBS electrode localization and stimulation-related variables to address the potential influence of surgical and programming factors on postoperative outcome. Active contact coordinates did not significantly differ between optimal and suboptimal responders, suggesting that gross between-group differences in active contact location were unlikely to fully explain the observed model performance. However, suboptimal responders showed numerically larger VTA volume and VTA-STN overlap, particularly within the motor STN subregion. These findings should be interpreted cautiously because they were exploratory and did not remain significant after correction for multiple comparisons. One possible explanation is that patients with poorer motor response required broader or stronger stimulation during postoperative programming. We did not systematically model stimulation-induced collateral effects such as dysarthria, mood changes or cognitive/behavioural adverse effects, which may also depend on contact location and current spread. Future studies should integrate preoperative imaging features, electrode localization, stimulation parameters, VTA-based metrics and adverse-effect profiles into a multimodal prognostic framework.

This study has several limitations. Firstly, the sample size was relatively small, which may limit the generalizability of the proposed model and increase the risk of overfitting. Although 5-fold cross-validation was used to mitigate this issue, the findings should still be interpreted cautiously. More cases and a longer study period are required to further verify the robustness and effectiveness of the model. Secondly, no independent external validation cohort was available in the present study. The expanded 60-patient analysis was based on the same single-centre retrospective cohort and should therefore be interpreted as an internal scalability analysis rather than external validation. Future multicentre prospective studies are needed to assess the robustness, reproducibility and transportability of the proposed framework. Thirdly, the postoperative follow-up period in this study was limited to 1 year, and a longer follow-up period would be essential to assess the long-term prognosis of patients. Additionally, although non-motor symptoms, motor complications and quality-of-life measures were explored in supplementary analyses, these outcomes were not incorporated into the present prediction model and were limited by retrospective data availability. The current framework was also primarily developed using imaging features from preoperative SWI and did not fully integrate other potentially relevant clinical, neurophysiological, surgical or postoperative programming variables, such as active-contact localization, VTA distribution, electric-field metrics and longitudinal stimulation settings. Although these stimulation-related variables were explored in supplementary analyses, they were not incorporated into the present prediction model. Future studies may explore multimodal fusion strategies combining imaging biomarkers with clinical and surgical information to further improve predictive performance. Importantly, the proposed model is intended to assist rather than replace clinical decision-making. Prognostic predictions should be interpreted in the context of comprehensive clinical evaluation, surgical planning, patient preference and multidisciplinary judgment.

In conclusion, our study demonstrated that preoperative SWI images covering the SNAS can be used for prognostic prediction of STN-DBS motor outcome in Parkinson’s disease. To improve predictive performance, we proposed a deep learning-based multi-task fusion network that formulated prognostic prediction as a classification task and incorporated complementary regression learning based on continuous motor improvement rates. The proposed framework achieved a prognostic prediction accuracy of 88.3%, suggesting that it may provide a novel and practical decision-support tool for neurosurgeons and aid in identifying suitable patients with Parkinson’s disease for surgery. Overall, the framework provides a promising imaging-based strategy for preoperative prognostic assessment of STN-DBS in Parkinson’s disease, although further external validation and methodological refinement are still required before routine clinical application.

## Supplementary Material

fcag333_Supplementary_Data

## Data Availability

The clinical and imaging data supporting the findings of this study are available from the corresponding author upon reasonable request, subject to institutional and ethical restrictions. The code used for model training, performance evaluation, statistical analysis, and DBS-related metric extraction is available at https://github.com/ceasorcai/PD. Clinical and imaging data are not publicly available because of institutional and ethical restrictions.
