# Supplementary material for: Development and application of a multi-task fusion model using susceptibility-weighted imaging-based substantia nigra and adjacent structures for prognostic prediction of subthalamic nucleus deep brain stimulation in Parkinson’s disease
Source: Brain Commun. 2026 Sep 11;8(5):fcag333. doi: 10.1093/braincomms/fcag333 (PMC13563298; doi:10.1093/braincomms/fcag333)
Supplement: Supplementary file 1 [file fcag333_supplementary_data.zip › Original Submission - Author's Response.pdf]

Response to Reviewers

Manuscript ID: BRAINCOM-2025-1391

Manuscript title: Development and Application of a Multi-Task Fusion Model Using SWI-based Substantia Nigra and Adjacent Structures for Prognostic Prediction of STN-DBS in Parkinson's Disease

Dear Prof. Houeto, Prof. Belin, the Scientific Editor, and Reviewers,

We sincerely thank the editors and reviewers for their careful evaluation of our manuscript and for the constructive comments and suggestions. We have carefully revised the manuscript according to all comments. The revision has substantially improved the clarity, methodological transparency, clinical relevance, and reporting quality of the study.

The major revisions include the following:

1. We clarified the comparison with the baseline model and added a dedicated description of the 3D ResNet50 baseline model.
2. We expanded the technical description of the proposed multi-task classification-regression fusion framework, including model architecture, patient-level data splitting, five-fold cross-validation, data augmentation, training details, loss functions, and evaluation metrics.
3. We clarified that the expanded 60-patient analysis represents an internal scalability analysis rather than external validation, because no independent external validation cohort was available.
4. We added additional clinical outcome analyses beyond UPDRS-III, including LEDD, UPDRS-IV, NMSS, PDQ-39, MMSE, MoCA, HAMD, HAMA, PDSS, and

constipation score.

5. We clarified clinical data acquisition, including the use of the original UPDRS rather than the MDS-UPDRS, medication-off and stimulation-on/medication-off assessment states, assessment timing, and blinding of raters to imaging features and model outputs.
6. We expanded the Introduction to explain the biological and clinical rationale for using SWI of the substantia nigra and adjacent structures.
7. We added a retrospective swallow-tail sign analysis performed by blinded radiologists.
8. We added DBS electrode localization and stimulation-related analyses, including electrode type, active contact coordinates, stimulation parameters, VTA metrics, and VTA-STN overlap analyses.
9. We provided individual preoperative and postoperative UPDRS-III scores and continuous improvement rates in the supplementary material.
10. We added supplementary figures showing the SNAS-centered sub-volume in axial, coronal, and sagittal views and group-level Lead-DBS reconstruction.
11. We supplied a graphical abstract, an abbreviated summary, STROBE checklist, TRIPOD-AI checklist, and code availability information according to the journal requirements.
12. During revision, we identified and corrected a known age entry error for one patient in the original clinical table. The descriptive age statistics and the corresponding baseline comparison results were therefore updated in the revised Table 1 and

Supplementary Table 1. This correction did not affect the UPDRS-III outcome definition, responder classification, model training labels, model performance results, or the main conclusions of the study.

All changes have been incorporated into the revised manuscript and supplementary files. Below, we provide a point-by-point response.

Response to Reviewer 1

We thank Reviewer 1 for the constructive and insightful comments. We have revised the manuscript to clarify the baseline model, model architecture, clinical outcome assessment, clinical data acquisition, and the biological rationale for SWI-based SNAS imaging.

Comment 1

The comparison with the "baseline" model is not clearly described in the Methods section.

Response:

We thank the reviewer for this important comment. We have added a dedicated subsection entitled **"Baseline model"** in the Methods section. We clarified that a 3D ResNet50 model was used as the baseline classification model because the primary prognostic prediction task was formulated as a binary classification problem. The baseline model used the same SNAS-centered 3D SWI sub-volumes as input and was trained and evaluated using the same patient-level data splitting strategy, five-fold cross-validation procedure, data augmentation strategy, and performance metrics as the proposed multi-task fusion framework.

Revision made:

We added the following text to the Methods section:

“Because the primary prognostic prediction task was formulated as a binary classification problem, a 3D ResNet50 model was used as the baseline classification model. The same SNAS-centered 3D SWI sub-volumes were used as input for the baseline model. The baseline model was trained and evaluated using the same patient-level data splitting strategy, five-fold cross-validation procedure, data augmentation strategy, and performance metrics as the proposed multi-task fusion framework. The architecture of the 3D ResNet50 baseline model is shown in Supplementary Figure 1.”

Comment 2

The 3D ResNet50 architecture reported in the Results is not adequately described in the Methods.

Response:

We agree with the reviewer. We have now described the baseline model in the Methods section and added its architecture as **Supplementary Figure 1**. The supplementary figure illustrates that the 3D SWI sub-volume was processed by an initial convolutional block, four Bottleneck3D residual stages, global average pooling, and a linear classification head to predict optimal versus suboptimal motor response.

Revision made:

We added **Supplementary Figure 1**, entitled:

“Architecture of the 3D ResNet50 baseline model.”

The figure legend states:

“The 3D SWI sub-volume was processed by an initial convolutional block, four Bottleneck3D residual stages, global average pooling, and a linear classification head to predict optimal versus suboptimal motor response. BN indicates batch normalization.”

Comment 3

No clinical outcome measures other than the UPDRS-III are included. Data of motor fluctuations changes, PROMs and quality of life measures should be implemented to understand the actual clinical relevance of the proposed approach.

Response:

We thank the reviewer for this helpful suggestion. To better evaluate the clinical relevance of the response classification beyond UPDRS-III, we added exploratory analyses of additional clinical outcomes. These included LEDD, UPDRS-IV as an available measure of motor complications, NMSS, PDQ-39 as a disease-specific patient-reported quality-of-life measure, MMSE, MoCA, HAMD, HAMA, PDSS, and constipation score.

The results are now reported in the revised Results section and detailed in **Supplementary Table 2**. In brief, optimal responders showed greater improvement in quality of life as measured by PDQ-39. Postoperative PDQ-39 scores were lower in optimal responders than in suboptimal responders, and the percentage favorable change in PDQ-39 remained significant after false discovery rate correction. Changes in LEDD, UPDRS-IV, NMSS, MMSE, MoCA, HAMD, HAMA, PDSS, and constipation score did not significantly differ between groups.

Revision made:

We added a new Methods subsection:

“Additional clinical outcome analyses”

We added a new Results subsection:

“Additional clinical outcomes beyond UPDRS-III”

We also added **Supplementary Table 2**, entitled:

“Additional clinical outcomes beyond UPDRS-III.”

Comment 4

The methodology for clinical data acquisition is not reported, e.g., how and when UPDRS-III scores were assessed; is UPDRS or MDS-UPDRS?

Response:

We agree that this information should be clearly stated. We have revised the Methods section to clarify that the original UPDRS, rather than the Movement Disorder Society-sponsored revision of the UPDRS, was used in this cohort. We also clarified that preoperative UPDRS-III scores were assessed in the medication-off state after withdrawal of dopamine agonists for 72 hours and other dopaminergic medications for at least 12 hours. Postoperative motor outcome was assessed at 1 year after surgery in the stimulation-on/medication-off condition after chronic stimulation parameters had been optimized. Clinical assessments were performed by raters blinded to imaging features and model outputs.

Revision made:

The revised Methods section now states:

"The original Unified Parkinson's Disease Rating Scale (UPDRS), rather than the Movement Disorder Society-sponsored revision of the UPDRS, was used in this cohort. Preoperative UPDRS-III scores were assessed in the medication-off state after withdrawal of dopamine agonists for 72 hours and other dopaminergic medications for at least 12 hours."

The revised Methods section also states:

"Postoperative motor outcome was assessed at 1 year after surgery in the stimulation-on/medication-off condition after chronic stimulation parameters had been optimized. Clinical assessments were performed by raters blinded to imaging features and model outputs."

Comment 5

The Introduction does not sufficiently explain why SWI imaging of the substantia nigra is expected to be informative, nor the biological or clinical rationale underlying this choice.

Response:

We thank the reviewer for this important suggestion. We have expanded the Introduction to explain the biological and clinical rationale for using SWI features of the substantia nigra and adjacent structures. We now discuss that the substantia nigra is pathologically central to Parkinson's disease, with degeneration of dopaminergic neurons, α-synuclein pathology, and abnormal iron accumulation. SWI is sensitive to local magnetic susceptibility changes and may indirectly reflect iron-related alterations in the substantia nigra and nigrosome-1

region. Although the subthalamic nucleus is the surgical target of STN-DBS, the substantia nigra and adjacent structures are closely connected with basal ganglia motor circuits and may contain imaging features related to disease severity, circuit integrity, and postoperative motor responsiveness.

Revision made:

We added the following paragraph to the Introduction:

"The substantia nigra is pathologically central to Parkinson's disease, with degeneration of dopaminergic neurons, α-synuclein pathology, and abnormal iron accumulation. Susceptibility-weighted imaging is sensitive to local magnetic susceptibility changes and can indirectly reflect iron-related alterations in the substantia nigra and nigrosome-1 region. Although the subthalamic nucleus is the surgical target of STN-DBS, the substantia nigra and its adjacent structures are closely connected with basal ganglia motor circuits and may contain imaging features related to disease severity, circuit integrity, and postoperative motor responsiveness. Therefore, preoperative SWI features covering the substantia nigra and adjacent structures may provide biologically plausible prognostic information for STN-DBS outcome prediction."

Response to Reviewer 2

We thank Reviewer 2 for the detailed and constructive critique. We have substantially revised the manuscript to improve the reporting of electrode type, surgical reconstruction, data splitting, model details, individual outcome values, swallow-tail sign analysis, DBS electrode and stimulation-related analyses, and visualization of the SNAS region.

Comment 1

Patients undergo STN-DBS, but the type of electrodes is not specified, directional or not, MEDTRONIC, Boston Sc.?

Response:

We thank the reviewer for pointing this out. We have now specified the electrode type in the Methods section. All patients received bilateral STN-DBS using non-directional Pins L301 electrodes.

Revision made:

The Methods section now states:

“Non-directional Pins L301 electrodes (Pins Medical, China) were used in all patients.”

Comment 2

It is said that the postop CT was registered to the preop T2 MRI, validating the adherence of electrode implantation to the planned trajectory. Where are the data? Registering does not prove that the implantation went well.

Response:

We agree with the reviewer that image registration alone does not prove successful implantation and that additional electrode localization data are needed. We have revised the manuscript to avoid overclaiming and added Lead-DBS-based reconstruction and quantitative electrode localization analyses.

Postoperative CT images were co-registered with preoperative MRI, and electrode

trajectories and active contacts were retrospectively reconstructed using Lead-DBS. We added a group-level reconstruction figure as **Supplementary Figure 3** and quantitative active contact MNI coordinates as **Supplementary Table 5**. These analyses showed no significant between-group differences in active contact coordinates along the x, y, or z axes.

Revision made:

We added the following text to the Methods section:

“Postoperative CT images were co-registered with preoperative MRI to evaluate lead location. Active contact coordinates and stimulation-related metrics were retrospectively reconstructed using Lead-DBS. The active contact coordinates were transformed into MNI space.”

We added a new Results subsection:

“DBS electrode localization and stimulation-related analyses”

We also added:

“Supplementary Figure 3. Group-level Lead-DBS reconstruction of bilateral STN-DBS electrodes and active contacts.”

and:

“Supplementary Table 5. Active contact MNI coordinates in optimal and suboptimal responders.”

Comment 3

About the model: the 3D CNN with multi-task classification-regression fusion is conceptually sound but critically underspecified. Training on only a few cases and a small

sample size with no external validation makes generalizability claims unsupportable. Combined with unclear data splitting, tiny sample size and inadequate technical detail, this manuscript cannot be assessed for validity or contribution.

Response:

We appreciate this important critique. We have substantially revised the Methods section to provide more technical details regarding the model architecture, training strategy, patient-level data splitting, cross-validation, data augmentation, optimizer settings, loss functions, regression target, and evaluation metrics.

We clarified that five-fold cross-validation was performed at the patient level, with a 4:1 training/testing split in each fold, ensuring that images from the same patient did not appear in both training and testing subsets. Data augmentation was applied only to the training data in an on-the-fly manner. We also clarified that the expanded 60-patient analysis should be interpreted as an **internal scalability analysis** rather than external validation because no independent external cohort was available.

We also revised the Discussion to more cautiously discuss the small sample size, risk of overfitting, and absence of external validation.

Revision made:

The revised Methods section now states:

"A five-fold cross-validation strategy was used to evaluate model performance. In each fold, the dataset was split at the patient level into training and testing subsets with a ratio of 4:1, ensuring that images from the same patient did not appear in both training and testing subsets. Data augmentation was applied only to the training data in an on-the-fly manner,

including random elastic deformation, random rotation, random scaling, and random mirroring.”

We also added:

“Because no independent external cohort was available, the expanded 60-patient analysis should be interpreted as an internal scalability analysis rather than external validation.”

The Discussion now includes:

“Firstly, the sample size was relatively small, which may limit the generalizability of the proposed model and increase the risk of overfitting. Although five-fold cross-validation was used to mitigate this issue, the findings should still be interpreted cautiously.”

and:

“Secondly, no independent external validation cohort was available in the present study. The expanded 60-patient analysis was based on the same single-center retrospective cohort and should therefore be interpreted as an internal scalability analysis rather than external validation.”

Comment 4

Looking at Table 1, standard deviations of UPDRS-III scores of the optimal vs suboptimal groups are much larger than the difference of the means. Individual values should be provided. Taking into account these values in the model, instead of considering 2 groups, should at least be discussed.

Response:

We agree with the reviewer. We have added individual preoperative UPDRS-III scores, 1-

year postoperative stimulation-on/medication-off UPDRS-III scores, continuous UPDRS-III improvement rates, and binary response classifications in **Supplementary Table 3**.

We also clarified that the continuous improvement rate was incorporated into the model through the regression branch as complementary learning information, while the binary responder label was used as the primary classification endpoint. In the Discussion, we now explicitly acknowledge that dichotomizing a continuous motor improvement measure may lead to loss of information, and we explain how the multi-task framework partly addresses this issue by using the continuous improvement rate in the regression branch.

Revision made:

We added **Supplementary Table 3**, entitled:

"Individual preoperative and postoperative UPDRS-III scores and continuous improvement rates."

We added the following Results subsection:

"Individual UPDRS-III values and continuous improvement rates"

We also added the following text to the Discussion:

"We acknowledge that dichotomizing a continuous motor improvement measure may lead to loss of information. To partly address this issue, individual continuous UPDRS-III improvement rates are now provided, and the regression branch of the proposed multi-task framework incorporated the continuous improvement rate as complementary learning information. Nevertheless, responder classification was retained as the primary endpoint because it provides an intuitive and clinically interpretable threshold for identifying patients with suboptimal motor benefit after STN-DBS."

Comment 5

Omission of swallow tail sign analysis. The swallow tail sign is considered the gold-standard SWI biomarker for PD. This was not mentioned or discussed. A retrospective swallow tail rating by blinded neuroradiologists, correlation with outcomes, comparison to the model's accuracy could be done.

Response:

We thank the reviewer for this important suggestion. We have added a retrospective swallow-tail sign analysis. The left and right swallow-tail signs were rated on preoperative SWI images by two radiologists who were blinded to postoperative motor outcomes and model predictions, with final ratings determined by consensus.

We compared left, right, and total swallow-tail scores between optimal and suboptimal responders, performed Spearman correlation analyses with continuous UPDRS-III improvement rates, and conducted ROC analyses for prognostic discrimination. The results are reported in the revised Results section and detailed in **Supplementary Table 4**. In brief, swallow-tail scores did not significantly differ between optimal and suboptimal responders, were not significantly correlated with continuous motor improvement, and showed near-chance prognostic discrimination, with AUC values of 0.503, 0.533, and 0.538 for left, right, and total scores, respectively.

Revision made:

We added a new Methods subsection:

“Swallow-tail sign analysis”

We added a new Results subsection:

“Swallow-tail sign analysis”

We added **Supplementary Table 4**, entitled:

“Swallow-tail sign analysis.”

Comment 6

Omission of STN-DBS results: even if this study is using preop SWI images to predict the outcome of the STN-DBS in PD patients, the authors should show informations about the implanted electrodes, coordinates of the active contact, settings of the stimulation, anatomical localisation of the active contact and discuss them. For example, is anatomical localisation consistent for the whole group? No significative difference of coordinates of the active contact between the 2 groups? Also, you should discuss about collateral effects of STN-DBS that may occur.

Response:

We agree that surgical and stimulation-related factors are important for interpreting DBS outcomes. We have added detailed DBS electrode localization and stimulation-related analyses.

All patients were implanted with bilateral non-directional Pins L301 electrodes. Postoperative CT-MRI fusion and Lead-DBS-based reconstruction were used to evaluate electrode trajectories, active contact coordinates, stimulation parameters, VTA metrics, and VTA-STN overlap. A group-level reconstruction is provided in **Supplementary Figure 3**. Active contact coordinates in MNI space are summarized in **Supplementary Table 5**.

Stimulation parameters, VTA metrics, and VTA-STN overlap variables are summarized in **Supplementary Table 6**. Patient-level DBS stimulation parameters, active contact coordinates, VTA metrics, and VTA-STN overlap values are provided as a separate Excel file, **Supplementary Table 7**.

The revised Results section reports that no significant between-group differences were observed in active contact coordinates. Some VTA-related metrics were numerically larger in suboptimal responders, but these findings did not remain significant after false discovery rate correction and were interpreted as exploratory.

We also added discussion of possible stimulation-induced collateral effects, including dysarthria, mood changes, and cognitive/behavioral adverse effects, and emphasized that future studies should incorporate adverse-effect profiles and stimulation-related variables into multimodal prognostic models.

Revision made:

We added the following supplementary materials:

"Supplementary Figure 3. Group-level Lead-DBS reconstruction of bilateral STN-DBS electrodes and active contacts."

"Supplementary Table 5. Active contact MNI coordinates in optimal and suboptimal responders."

"Supplementary Table 6. Stimulation parameters, VTA metrics, and VTA-STN overlap analyses by outcome group."

"Supplementary Table 7. Patient-level DBS stimulation parameters, active contact coordinates, VTA metrics, and VTA-STN overlap values."

The Discussion now states:

“We also performed exploratory analyses of DBS electrode localization and stimulation-related variables to address the potential influence of surgical and programming factors on postoperative outcome. Active contact coordinates did not significantly differ between optimal and suboptimal responders, suggesting that gross between-group differences in active contact location were unlikely to fully explain the observed model performance.”

The Discussion also states:

“We did not systematically model stimulation-induced collateral effects such as dysarthria, mood changes, or cognitive/behavioral adverse effects, which may also depend on contact location and current spread. Future studies should integrate preoperative imaging features, electrode localization, stimulation parameters, VTA-based metrics, and adverse-effect profiles into a multimodal prognostic framework.”

Comment 7

Minor request: show SNAS axially, coronally and sagittally. Does it include the STN?

Response:

We thank the reviewer for this helpful suggestion. We have added **Supplementary Figure 2**, which shows representative SNAS-centered sub-volume extraction from preoperative SWI images in axial, coronal, and sagittal views. We also clarified in the Methods section and supplementary figure legend that the SNAS-centered sub-volume covered the substantia nigra and adjacent deep brain structures, including part of the STN region.

Revision made:

We added **Supplementary Figure 2**, entitled:

“Representative illustration of SNAS-centered sub-volume extraction from preoperative SWI.”

The revised Methods section now states:

“The SNAS-centered sub-volume was designed to include the substantia nigra and adjacent deep brain structures, including part of the STN region.”

Response to the Scientific Editor

We thank the Scientific Editor for the detailed guidance regarding journal formatting, reporting guidelines, graphical abstract, abbreviated summary, statistical reporting, figure formatting, and code availability. We have revised the manuscript and submission files accordingly.

Comment 1

Please supply a graphical abstract. A graphical abstract is a visual representation of the central finding or methodology of the paper. It will be displayed in the table of contents. Authors should strive to make the graphical abstract informative, interesting, visually appealing, and straightforward.

Response:

We have prepared and uploaded a graphical abstract as a separate file. The graphical abstract summarizes the central methodology and main finding: preoperative susceptibility-weighted magnetic resonance imaging of the substantia nigra and adjacent

structures was analyzed using a multi-task deep learning model to predict 1-year motor outcome after subthalamic deep brain stimulation.

Revision made:

A separate graphical abstract file has been uploaded.

Comment 2

Please add an Abbreviated Summary, up to 50 words, of your paper that captures the main purpose and conclusions of the work. The summary should state the main results written in the third person and should not contain any abbreviations other than those on the accepted abbreviations list. Please upload it as a Word document.

Response:

We have prepared and uploaded an abbreviated summary as a separate Word document. It is written in the third person, contains fewer than 50 words, and avoids non-allowed abbreviations except MRI, which is included in the journal's accepted abbreviation list.

Revision made:

The uploaded abbreviated summary reads:

"Xiong et al. report that susceptibility-weighted MRI of the substantia nigra and adjacent structures, analyzed with a multi-task deep learning model, predicts motor outcome after subthalamic nucleus deep brain stimulation in Parkinson's disease. This imaging-based framework may support preoperative patient selection and prognostic assessment."

Comment 3

Please include the appropriate checklist to comply with our ethical standards.

Response:

We have completed and uploaded the appropriate reporting guideline checklists. Because this study is a single-center retrospective cohort study involving a prediction model developed using artificial intelligence/deep learning methods, we provided both a STROBE cohort checklist and a TRIPOD-AI checklist.

Revision made:

We uploaded:

“STROBE_checklist_filled_cohort.docx”

and:

“TRIPOD_AI_checklist_filled.docx”

Comment 4

For each statistical analysis in a figure, please report in the figure legend, where missing, the test used and relevant test statistic values alongside p values. Also provide details of the experimental unit for each group analysed in these plots, and if the number is <10 show the single data-points.

Response:

We reviewed all figures and figure legends. The main figures are primarily schematic diagrams, anatomical region illustrations, ROC curves, and feature-map visualizations. They do not present between-group statistical comparisons requiring test statistics in the figure legends. Statistical analyses are reported in the Results and in

Tables/Supplementary Tables, including test methods, P values, and where applicable effect sizes and false discovery rate correction. The experimental unit throughout the prediction analyses was the patient. Individual patient-level UPDRS-III values are now provided in Supplementary Table 3, and patient-level DBS metrics are provided in Supplementary Table 7.

Revision made:

We expanded the Statistical analysis subsection in the Methods section and provided additional quantitative results in Supplementary Tables 1–7. The ROC curve legend was revised to clarify that thin lines represent individual folds and the thick blue line represents the mean ROC curve.

Comment 5

If you plan to edit the current figures and tables or make new figures and tables, please ensure that these items fit a typeset journal page, or are uploaded as supplementary material. Please view your figures at this size and make sure that all lettering and text is large enough to read.

Response:

We have checked and revised the figures for readability and formatting. Large tables and additional analyses have been provided as supplementary material to avoid exceeding the size of a typeset journal page. Supplementary Table 7 is provided as a separate Excel file because it contains patient-level DBS stimulation parameters, active contact coordinates, VTA metrics, and VTA-STN overlap values.

Revision made:

We uploaded revised figures and supplementary files, including the supplementary material PDF and Supplementary Table 7 as a separate Excel file.

Comment 6

Please note our policy on code availability. If any code was generated for data analysis for this manuscript, please make this code available, either via an online repository and provide a link within the data availability statement, or within the manuscript.

Response:

We have added a Code availability section to the revised manuscript. The code used for model training, performance evaluation, statistical analysis, and DBS-related metric extraction is available through an online repository.

Revision made:

The revised manuscript now includes:

"The code used for model training, performance evaluation, statistical analysis, and DBS-related metric extraction is available at https://github.com/ceasorcai/PD. Clinical and imaging data are not publicly available because of institutional and ethical restrictions."

Additional formatting and reporting revisions

In addition to the scientific revisions described above, we revised the manuscript to improve compliance with the Brain Communications formatting checklist. Specifically:

1. We added a short title to the title page.

2. We revised the abstract into an unstructured format.
3. We revised the keywords.
4. We added Data availability and Code availability sections.
5. We placed the Funding section before the Competing interests section.
6. We revised the figure legends and placed them after the references.
7. We added short titles to each figure legend.
8. We uploaded the graphical abstract and abbreviated summary as separate files.
9. We uploaded the STROBE and TRIPOD-AI reporting checklists.
10. We provided Supplementary Table 7 as a separate Excel file.

Closing statement

We are grateful to the editors and reviewers for their valuable comments, which have substantially improved the manuscript. We believe that the revised manuscript is clearer, more transparent, and more clinically informative. We hope that the revised version is now suitable for further consideration by **Brain Communications**.

Sincerely,

Chi Xiong, Kun Wang, Erkang Cheng, Zhiyong Sun, Bin Cai, Chaoshi Niu, and Bo Song

On behalf of all authors
