# Supplementary material for: Development and application of a multi-task fusion model using susceptibility-weighted imaging-based substantia nigra and adjacent structures for prognostic prediction of subthalamic nucleus deep brain stimulation in Parkinson’s disease
Source: Brain Commun. 2026 Sep 11;8(5):fcag333. doi: 10.1093/braincomms/fcag333 (PMC13563298; doi:10.1093/braincomms/fcag333)
Supplement: Supplementary file 1 [file fcag333_supplementary_data.zip › Original Submission - Decision Letter.pdf]

Original Submission - Decision Letter

23-Apr-2026

Dear Prof. song,

Thank you for your recent submission BRAINCOM-2025-1391 entitled "Development and Application of a Multi-Task Fusion Model Using SWI-based Substantia Nigra and Adjacent Structures for Prognostic Prediction of STN-DBS in Parkinson's Disease" to Brain Communications.

Your manuscript has been reviewed, and while the reviewers and editorial team appreciate many aspects of the paper, they raise some concerns that you will need to address in order for the paper to be considered for publication.

If you can address these concerns, I invite you to submit a revised manuscript and a response to each of the reviewers' comments. The reviewer(s)' comments are included at the bottom of this message.

In addition to the reviewers' comments, please also address any comments from our Scientific Editor added below to comply with our requirements for presentation of scientific data at Brain Communications. Please note we will not accept the paper unless you comply with our policies on data analysis and presentation as noted in the comments from the Scientific Editor.

Please format your revised manuscript according to our Instructions to Authors https://academic.oup.com/braincomms/pages/General_Instructions
A summary of key points is attached to help with this. Please note that there can be substantial delays to publication if you don't resubmit in line with our requirements.

To revise your manuscript, click the link below or log into https://mc.manuscriptcentral.com/braincom and enter your Author Centre, where you will find your manuscript title listed under "Manuscripts with Decisions." Under "Actions," click on "Create a Revision." Your manuscript number will be appended to denote a revision.

*** PLEASE NOTE: This is a two-step process. After clicking on the link, you will be directed to a webpage to confirm. ***

https://mc.manuscriptcentral.com/braincom?URL_MASK=5a6629dc3b0346dc8455c6c3a68e6204

Please upload a revised manuscript with changes tracked with track changes or in coloured text.

When submitting your revised manuscript, you will be asked to respond to the reviewer(s) comments. Please be as specific as possible in your response to all of the comments.

The editorial team at Brain Communications aims to increase transparency in scientific publishing and to demonstrate the value that peer review brings to the scientific process. Please therefore consider whether you are willing to have reviewer(s)' comments on your paper and your response published as supplementary data with the paper if it is accepted. This is entirely optional and will only be done if both the authors and reviewers agree. While submitting your revision there will be an opportunity to review or update the answer you gave previously, if necessary.

IMPORTANT: Your original files are available to you when you upload your revised manuscript. Please delete any redundant files before completing the submission.

Because we are trying to facilitate timely publication of manuscripts submitted to Brain Communications, we would appreciate your revised manuscript as soon as possible. If it is not possible for you to resubmit within 3 months, the option to submit a revision will expire, and we would then consider your revised

paper as a new submission. However, if you need a little more time, please get in touch and we can extend this deadline for you.

Please note that Brain Communications is a fully open access journal, and if your article is accepted for publication, an open access charge will be payable (this does not apply to letters and scientific commentaries). Details can be found in our Instructions for Authors: https://academic.oup.com/braincomms/pages/General_Instructions

Once again, thank you for submitting your manuscript to Brain Communications and I look forward to receiving your revision.

Best wishes

Prof. Jean-Luc Houeto
Associate Editor, Brain Communications

Prof. David Belin
Editor-in-Chief Brain Communications

Reviewer(s)' Comments to Author:

Reviewer: 1 (Carlo Artusi)

Comments to the Author
The authors propose a novel prognostic deep learning model based on automatic SWI imaging to identify STN-DBS patients with a suboptimal response, defined as a <50% improvement in UPDRS-III scores comparing the presurgical Med-OFF and 1-year post-surgical StimON–Med-OFF conditions. The model achieved an accuracy of 85% (compared with a 57.5% baseline) and an F1 score of 0.887, corresponding to a 9.7% absolute improvement in accuracy.
The concept underlying the study is interesting. However, several aspects require careful clarification and further consideration, representing important study limitations:
- The comparison with the “baseline” model is not clearly described in the Methods section.
- The 3D ResNet50 architecture reported in the Results is not adequately described in the Methods.
- No clinical outcome measures other than the UPDRS-III are included. Data of motor fluctuations changes, PROMs and quality of life measures should be implemented to understand the actual clinical relevance of the proposed approach.
- The methodology for clinical data acquisition is not reported (e.g., how and when UPDRS-III scores were assessed; is UPDRS or MDS-UPDRS?).
- The Introduction does not sufficiently explain why SWI imaging of the substantia nigra is expected to be informative, nor the biological or clinical rationale underlying this choice.

Reviewer: 2

Comments to the Author
In this study, the authors propose an original deep learning framework utilizing Substantia Nigra and Adjacent Structures (SNAS) susceptibility-weighted imaging (SWI) signatures for predicting motor outcomes following subthalamic nucleus deep brain stimulation (STN-DBS) in Parkinson’s disease (PD) patients. This is a single-center retrospective study: 36 patients for validation, 60 patients for generalizability.
Patients were stratified into 2 groups (optimal vs suboptimal) based on their UPDRS-III before and after STN-DBS.

The proposed multi-task fusion model achieved 85% accuracy. SN-centric subvolumes provided the best prognostic accuracy.
The authors conclude that preop SWI around the SNAS can be used for prognostic prediction of STN-DBS for PD.
This is an important study as it reflects the urgent need for SWI biomarker in investigating Parkinson's disease.

But important informations are lacking: patients undergo STN-DBS, but the type of electrodes is not specified (directional or not, MEDTRONIC, Boston Sc. ?). It is said that the postop CT was registered the the preop T2 MRI, validating the adherence of electrode implantation to the planned trajectory. Where are the data? Registering doesn't prove that the implantation went well.

About the model: the 3D CNN with multi-task classification-regression fusion is conceptually sound but critically underspecified. Training on only a few cases and a small sample size with no external validation makes generalizability claims unsupportable. Combined with unclear data splitting, tiny sample size and inadequate technical detail, this manuscript cannot be assessed for validity or contribution.

Looking at table 1, standard deviations of UPDRS-III scores of the optimal vs suboptimal groups are much larger that the difference of the means. Individual values should be provided. Taking into account these values in the model, instead of considering 2 groups, should at least be discussed.

Finally, there are two major omissions:
- omission of swallow tail sign analysis. The swallow tail sign is considered the gold-standard SWI biomarker for PD (90-96% diagnostic accuracy). This was not mentioned or discussed. A retrospective swallow tail rating by blinded neuroradiologists, correlation with outcomes, comparison to the model's accuracy could be done.
- omission of STN-DBS results: even if this study is using preop SWI images to predict the outcome of the STN-DBS in PD patients, the authors should show informations about the implanted electrodes (coordinates of the active contact, settings of the stimulation, anatomical localisation of the active contact) and discuss them. Fro example, is anatomical localisation consistent for the whole group? No significative difference of coordinates of the active contact between the 2 groups? Also, you should discuss about colateral effects of STN-DBS that may occur.

Minor request: show SNAS axially, coronally and sagitally. Does it include the STN?

<strong>Comments from the Scientific Editor:</strong>

-Please supply a graphical abstract. A graphical abstract is a visual representation of the central finding or methodology of the paper. It will be displayed in the table of contents. Authors should strive to make the graphical abstract informative, interesting, visually appealing, and straightforward (no caption needed, no abbreviations except gene names). The technical requirements for the graphical abstracts are:
- Size: 120 x 120 mm (300-600dpi) or 3000 x 3000 pixels
- Font: Arial 12-16 pt
- File type: tiff or PDF
- Aspect ratio: 1:1 preferred

-Please add an Abbreviated Summary (up to 50 words) of your paper that captures the main purpose and conclusions of the work. The summary should state the main results written in the third person ('[1st author surname] et al. report that …') and conclusion. This summary will be used in the contents list online and should not contain any abbreviations other than those on the accepted abbreviations list and accepted gene/protein names, making it accessible to the non-specialist. Please upload it as a Word document. Allowed abbreviations: AIDS; ANOVA; AMPA; ATP; A,T,C,G; CNS; CSF; CT; DNA; ECG; EEG; EMG; GABA; HIV; MRI; NMDA; PET; PCR; RNA.

- Please include the appropriate checklist to comply with our ethical standards (see Reporting Guideline section within 'Instructions to authors' ).

-for each statistical analysis in a figure, please report in the figure legend, where missing the test used and relevant test statistic values alongside p values (for example F or t values for ANOVAs and t-tests). Also, please, provide details of the experimental unit for each group analysed in these plots, and if the number is <10 show the single data-points. Please note: We do not accept analyses where individual measurements are the experimental unit when there is likely to be biological variability between animals, culture replicates, etc. For this type of data, we require either that a mean or median is taken per animal/culture replicate followed by statistical analyses, or that more sophisticated analyses are performed such as mixed effects modelling which can account for random effects.

- As a reminder at this revision stage, if you plan to edit the current figures and tables or to make new figures and tables, please ensure that these items fit a typeset journal page (the area available for figure or table is 170 mm x 210 mm ), or are uploaded as supplementary material. Please view your figures at this size and make sure that all lettering and text is large enough to read.

- Please note our policy on code availability. If any code was generated for data analysis for this manuscript (e.g. using R or MATLAB or similar), please make this code available, either via an online repository, ad provide a link within the data availability statement, or within the manuscript.
