# Supplementary material for: Development and application of a multi-task fusion model using susceptibility-weighted imaging-based substantia nigra and adjacent structures for prognostic prediction of subthalamic nucleus deep brain stimulation in Parkinson’s disease
Source: Brain Commun. 2026 Sep 11;8(5):fcag333. doi: 10.1093/braincomms/fcag333 (PMC13563298; doi:10.1093/braincomms/fcag333)
Supplement: Supplementary file 1 [file fcag333_supplementary_data.zip › Original Submission.pdf]

OXFORD UNIVERSITY PRESS | Brain Communications

Development and Application of a Multi-Task Fusion Model Using SWI-based Substantia Nigra and Adjacent Structures for Prognostic Prediction of STN-DBS in Parkinson's Disease

Journal:	*Brain Communications*
Manuscript ID	BRAINCOM-2025-1391
Manuscript Type:	Original Article
Date Submitted by the Author:	08-Jan-2026

Complete List of Authors:	Xiong, Chi; Chinese Academy of Sciences Hefei Institutes of Physical Science, Institute of Intelligent Machines; The First Affiliated Hospital of USTC, Department of Neurosurgery<br>wang, kun; Chinese Academy of Sciences Hefei Institutes of Physical Science, Institute of Intelligent Machines<br>cheng, erkang; Chinese Academy of Sciences Hefei Institutes of Physical Science, Institute of Intelligent Machines<br>sun, zhiyong; Chinese Academy of Sciences Hefei Institutes of Physical Science, Institute of Intelligent Machines<br>cai, bin; Chinese Academy of Sciences Hefei Institutes of Physical Science, Institute of Intelligent Machines<br>Niu, Chaoshi; The First Affiliated Hospital of USTC, Neurosurgery<br>song, bo; Chinese Academy of Sciences Hefei Institutes of Physical Science, Institute of Intelligent Machines

Keywords:	Deep Brain Stimulation, Prognostic Prediction, Deep Learning, Susceptibility-Weighted Imaging, Parkinson's disease

SCHOLARONE™ Manuscripts

Development and Application of a Multi-Task Fusion Model Using SWI-based Substantia Nigra and Adjacent Structures for Prognostic Prediction of STN-DBS in Parkinson's Disease

Chi Xiong[1, 2] , Kun Wang[1] , Erkang Cheng[1] , Zhiyong Sun[1] , Bin Cai[1] *, Chaoshi Niu[2] *, Bo Song[1]*

[1]Institute of Intelligent Machines, HFIPS, Chinese Academy of Sciences, Hefei, China

[2]Department of Neurosurgery, The First Affiliated Hospital of USTC, Division of Life Sciences and Medicine, University of Science and Technology of China, Hefei, China

Correspondence to： DR. Chao-Shi Niu, Department of Neurosurgery, The First Affiliated Hospital of USTC, Division of Life Sciences and Medicine, University of Science and Technology of China, Hefei 230001 ，China. E-Mail: niuchaoshi@ustc.edu.cn.

Prof. Bin Cai, Institute of Intelligent Machines, HFIPS, Chinese Academy of Sciences, Hefei 230000,China.E-Mail: bcai@iim.ac.cn;

Prof. Bo Song, Institute of Intelligent Machines, HFIPS, Chinese Academy of Sciences, Hefei 230000,China.E-Mail: songbo@iim.ac.cn

For Review Only

[Abstract] Objective To develop and validate a deep learning framework utilizing Substantia Nigra and Adjacent Structures (SNAS) susceptibility-weighted imaging (SWI) signatures for predicting motor outcomes following subthalamic nucleus deep brain stimulation (STN-DBS) in Parkinson's disease (PD) patients.

Methods This study enrolled PD patients undergoing STN-DBS, stratified into Optimal and Suboptimal groups based on whether their Unified Parkinson's Disease Rating Scale Part III (UPDRS-III) score decreased by 50% or more during the medication-off (med-off) period one year post-surgery compared to baseline. We developed a deep learning-based multi-task fusion network integrating prognostic classification with complementary regression learning to predict surgical outcomes using preoperative SWI data covering the SNAS. Initial validation (n=36 cases from January 2019 to December 2021) demonstrated superior predictive performance of our multi-task fusion network compared to baseline models. Additionally, extended SNAS and non-SNAS images were used for training to investigate the specificity of SNAS in outcome prediction. The cohort was subsequently expanded to 60 cases (including 24 additional patients from January 2022 to December 2023) to validate the model's generalizability.

Results No significant differences were observed between the two groups in terms of age, gender, disease duration, preoperative UPDRS-III score, and the levodopa challenge test improvement rate ($P > 0.05$). Our multi-task fusion model achieved 85% accuracy (vs 57.5% baseline) and 0.887 F1-score, with 9.7% accuracy improvement through multi-loss optimization. Five-fold cross-validation demonstrated consistent performance (mean AUC:0.807). SN-centric subvolumes ($10 \times 40 \times 80$ voxels) showed 85.3% prognostic accuracy versus 62.5% for extranigral regions (AUC:0.87 vs 0.37, P<0.01). Expanded validation with 60 cases enhanced generalizability (88.3% accuracy, +3.9% vs baseline).

Conclusion: Preoperative SWI images that cover the SNAS can be used for prognostic prediction of STN-DBS for PD. The proposed model offers a novel and practical decision-support tool for neurosurgeons, aiding in the identification of suitable PD patients for surgery.

[Key words] Parkinson's disease; Deep Brain Stimulation; Prognostic Prediction; Deep Learning; Susceptibility-Weighted Imaging

Introduction

Parkinson's disease (PD) is a prevalent neurodegenerative condition that tends to exacerbate with the progression of its course. While pharmaceutical interventions demonstrate efficacy in the early stages, a significant number of patients necessitate Deep Brain Stimulation (DBS) to alleviate symptoms or mitigate side effects as the disease advances to an advanced stage, characterized by diminished efficacy and potential drug-related complications. The DBS emerges as a pivotal therapeutic intervention, supported by class I evidence, for alleviating motor symptoms associated with PD. The subthalamic nucleus (STN) is a commonly targeted area for DBS, with its electrical stimulation having been shown to significantly improve motor symptoms in patients with PD [1].

Despite its recognized benefits, the efficacy of DBS surgery exhibits significant heterogeneity[2]. Factors such as implantation accuracy and postoperative management are acknowledged as influential contributors to outcomes. Even in experienced centers, where these factors are meticulously managed, postoperative patients still manifest vastly different outcomes, implying the involvement of undisclosed factors[3] . Considering the invasiveness and potential risks associated with surgery and anesthesia, along with potential side effects such as the induction of mental and cognitive symptoms through STN-DBS[3], it is important to predict surgical efficacy accurately before operation to help decide whether to perform surgery.

In the current expert consensus and treatment guidelines for PD treatment with DBS, it lacks specific indications for efficacy prediction or recommendations of suitable prediction methods. Clinically, the widely employed levodopa challenge testing (LCT) serves as a common approach for estimating efficacy[4].Beyond its role as a diagnostic tool for Parkinson's disease, LCT necessitates an improvement rate of $\geqslant$ 30% before surgery is considered advisable. However, the predictive capacity of LCT for prognosis has been a subject of controversy, and recent studies generally indicate that it is a challenge task[5,6]. Because the mechanism of DBS in the treatment of PD differs from that of pharmacological interventions[7], it is essential to avoid confusion between the improvement rates, despite both interventions showing the capability to ameliorate patients' symptoms.

Given the inherent limitations of LCT, the pursuit of more accurate methods for preoperative prognostic prediction has become a critical focus of ongoing research in the field. Certain studies have utilized machine learning in combination with medical images to investigate strategies for prognostic prediction[8,9] or condition evaluation[10]. This study develops and validate a deep learning framework utilizing Substantia Nigra and Adjacent Structures (SNAS) susceptibility-weighted imaging (SWI) signatures for predicting motor outcomes following STN-DBS in PD, and the report is as follows.

Methods

Ethical approval

This study was approved by the Ethics Committee of the First Affiliated Hospital of USTC (Approval No.: 2022-RE-154). This retrospective study met the conditions for exemption from informed

For Review Only

consent, and obtained the informed consent exemption from the Ethics Committee.

Study design and patients

A single-center retrospective study was conducted to develop a deep learning model to predict the prognosis of PD patients undergoing STN-DBS. The dataset utilized for the development of the predictive system comprises 60 cases of bilateral STN DBS, all of them were got surgery during January 2019 to December 2023 in the Neurosurgery Department of the First Affiliated Hospital of USTC.The admission criteria of the patient included: (1) clinically diagnosed idiopathic PD based on the 2015 MDS PD diagnostic criteria[11]; (2) a disease duration of more than 5 years, with the modified Hoehn-Yahr Scale between 2.5 and 4; (3) absence of severe cognitive impairment [Montreal Cognitive Assessment (MoCA) score ⩾ 17, or Mini-mental State Examination (MMSE) score ⩾ 24][12]; (4) with no obvious mental disorders [Hamilton Anxiety Scale (HAMA) < 29,Hamilton Depression Scale(HAMD) < 21]; (5) no surgical contraindications; (6) no MRI contraindications; (7) Patient and family members cooperate with follow-up.

Treatment Protocol and Assessment of treatment response

All eligible patients received bilateral STN-DBS with microelectrode assistance. And all patients underwent their first programming session approximately one month postoperatively, then subsequent chronic parameter programming was adjusted based on the patients' symptoms until stable efficacy was achieved.

Demographic and clinical data, encompassing age, gender, disease duration, Hoehn-Yahr Scale, levodopa equivalent daily dose (LEDD), and Levodopa Challenge Test (LCT) responsiveness, were documented preoperatively. The baseline values for the preoperative Unified Parkinson’s Disease Rating Scale Part III scores were recorded during the medication-off state(med-off), involvinga 72-hour discontinuation of dopamine agonists and a12-hour discontinuation of other drugs.

Follow-up assessments were conducted when patients’ symptoms stabilized at one year postoperatively, and data on UPDRS III scores were obtained in the stimulation on (stim-on)/med-off state. Improvement (%) = [(preoperative UPDRS III score) − (postoperative UPDRS III score)] / (preoperative UPDRS III score) × 100%.

Patients were divided into optimal group and suboptimal group based on motor outcomes. Optimal outcomes were defined as improvements exceeding 50%, while suboptimal outcomes were characterized by improvements below 50%.

Data acquisition and preprocessing

All patients underwent preoperative 3T MRI scanning (US GE, MR750) with a 32-channel head coil. SWI Acquisitions Gradient echo sequences, using the following parameters: 104 slices, Field-of-view (FoV) = 240 mm, FoV phase 100%, Repetition time/Echo time = 39/23.5 ms; flip angle of 10°, sampling bandwidth = 162.73 Hz/pixel, Acquisition time was 4 min and 13 s for acquiring each SWI dataset.

To mitigate the impact of other brain tissues, only the 3D sub-regions within the SWI image covering the substantia nigra and adjacent structures(SNAS) are employed for the prognostic prediction. These 3D sub-regions were meticulously extracted by clinical surgeons from the First Affiliated Hospital of USTC as follows.

Firstly, clinical surgeons meticulously examined the 3D SWI image slice by slice to identify the

For Review Only

boundaries of the substantia nigra. Subsequently, a fixed-size 3D sub-volume encompassing both the left and right substantia nigra was extracted. The dimensions of this 3D sub-volume were set at 80 pixels in length, 40 pixels in width, and 10 pixels in height. Finally, to emphasize distinctions between pixel points and enhanced the network model's focus on overall image changes, facilitating smoother convergence and improved recognition rates, we normalized the 3D sub-volume by subtracting the mean value of the current pixel and dividing by the total standard deviation.

$$f(z,y,x) = \begin{cases} \frac{V_{z,y,x} - mean(V_{z,y,x})}{std(V_{z,y,x})}, & V_{z,y,x} > 0 \\ 0 & V_{z,y,x} = 0 \end{cases}$$

Model development

In this section, our solution for prognostic prediction of STN-DBS in PD is described. The overview, of our framework for prognostic prediction of STN-DBS in PD is shown in Fig. 1. It is a multi-task fusion framework which consists of four parts, the backbone for feature learning, the regression branch, the classification branch and a classifier. The regression branch is used to regress the motor outcome improvement rate scores, the classification branch is used to give a probability value for optimal or suboptimal DBS outcome improvement. Finally, a logistic regression classifier fuses the output of the regression branch and the classification branch to predict the motor outcome of STN-DBS in PD.

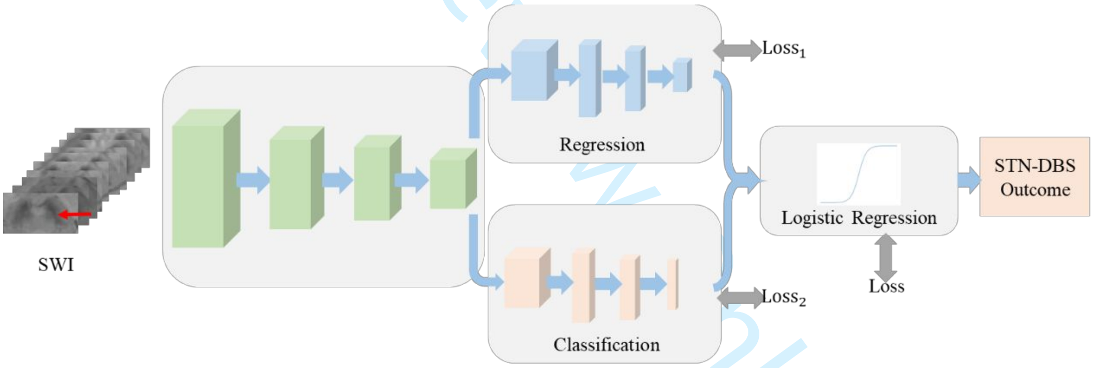

Figure 1: Illustration of our proposed framework for prognostic prediction of STN-DBS in PD. $Loss_1$: the regression loss, $Loss_2$: the classification loss, Loss: the logistic regression loss, Red arrow: substantia nigra.

We formulate the prognostic prediction as a classification task, as shown in Fig. 2. To enhance the representation ability of the neural network, the idea of complementary-task learning is introduced. The details of our multi-task fusion framework for the prognostic prediction of STN-DBS in PD is shown in Fig. 3. The regression branch acts as a complementary-task to improve the performance of the classification task.

For Review Only

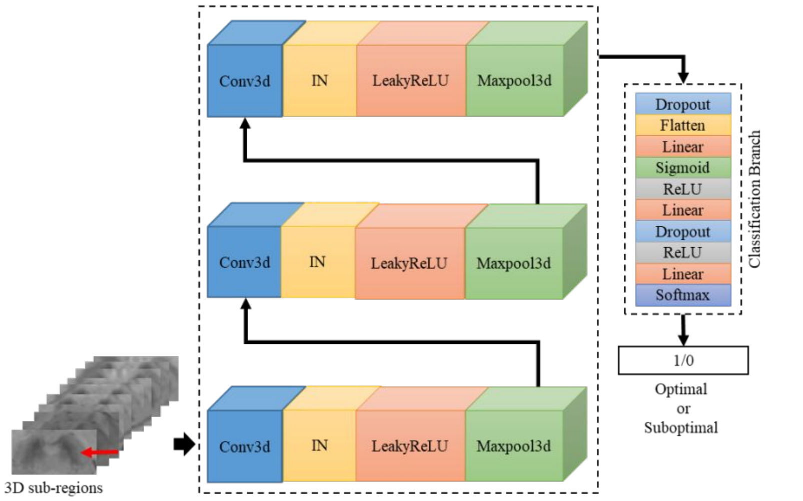

Figure 2: The architecture of the classification network consists of three linear convolution layers. The first two linear convolutions are followed by a Rectified Linear Unit (ReLU) operation, while the final linear convolution is followed by a softmax operation. Red arrow: substantia nigra.

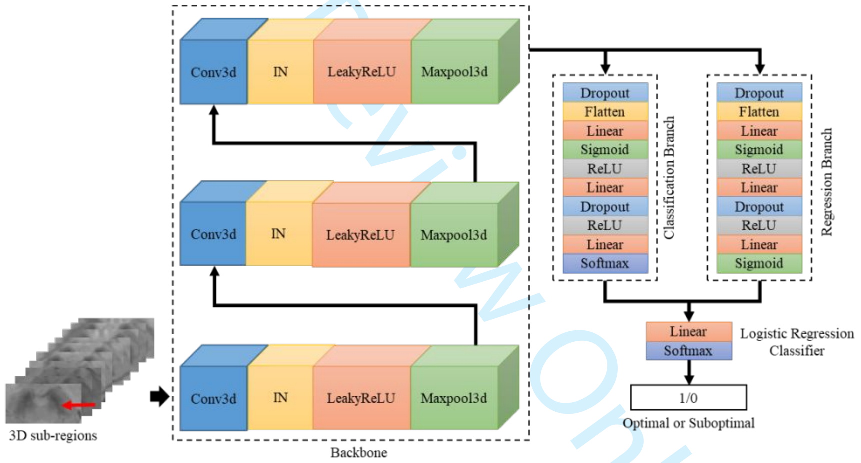

Figure 3: The structure of the proposed multi-task fusion framework
Red arrow: substantia nigra

For Review Only

To effectively leverage the 3D anatomical information of the substantia nigra, we employ a 3D convolution to construct the backbone. The 3D. convolutional block in the backbone primarily consists of 3D convolution, InstanceNorm (IN), and LeakyReLU. Each 3D convolutional block is followed by a Max Pooling operation. After three down sampling operations of the Max Pooling layer, the original size of the 3D sub-volume is reduced from $10\times40\times80$ to $5\times5\times10$. The output feature maps of the backbone serve as the input for the multi-task learning component. The classifier aggregates the outputs of the multi-task branches and determines the final improvement effect.

The multi-task learning component comprises two parallel branches, a classification branch and a regression branch. The classification branch utilizes the output of the backbone as input and produces the probability of classifying the deep brain stimulation outcome into either optimal or suboptimal categories. To enhance the representation ability of the backbone, and act as a

supplement to the final prediction results, a complementary-task learning regression branch is incorporated. This branch takes the output of the backbone as input and performs regression on the improvement rate scores, as shown in Fig. 3.

Lastly, a logistic regression classifier is introduced as the fusion phase. It takes the outputs of both the classification branch and the regression branch as input and produces the final prognostic prediction.

The loss function encompasses three optimization objectives corresponding to the tasks: two classification tasks and one regression task. For case i in the classification branch, with probability p_i and ground truth class y_i, the Cross Entropy loss is employed for the classification loss L_{cls}, and the logistic regression loss L_{log}. The SmoothL1 loss is utilized as the regression loss L_{reg} for regressing the improvement score. The final loss L_{total} is defined as:

$$L_{log} = L_{cls} = \frac{1}{N}\sum_i L_i = \frac{1}{N}\sum_i -[y_i \cdot \log(p_i) + (1 - y_i) \cdot \log(1 - p_i)]$$

$$L_{reg} = \begin{cases} 0.5 \cdot (y_i - p_i)^2 & if\ |y_i - p_i| < 1 \\ |y_i - p_i| - 0.5 & otherwise \end{cases}$$

$$L_{total} = \alpha \cdot L_{log} + \beta \cdot L_{cls} + \gamma \cdot L_{reg}$$

where α , β and γ are balancing parameters between the logistic regression loss ,the classification loss and the regression loss.

Implementation details and Evaluation Indicators

A five-fold cross-validation is used to investigate the prediction accuracy and the generalizability of the prediction network. We randomly split the cases for training and testing with a ratio of 4:1. We used the data augmentation strategies during training, including random elastic deformation, random rotation, random scaling and random mirroring. The data augmentation is performed “on-the-fly” during training. The network is optimized by Adam with an initial learning rate 0.01, and weight decay of 1e-4. Given that the proposed method employs a multi-task training strategy, the tasks mutually reinforce and compete with each other. To mitigate potential conflicts arising from competition, the balancing parameters α, β, and γ are dynamically adjusted to different values as the number of training epochs increases. To investigate the generalizability of the prediction network, we first selected 36 cases from January 2019 to December 2021 for training and testing at a ratio of 4:1. Subsequently, we increased the volume to 60 by adding 24 cases from January 2022 to December 2023, and all of them were utilized for training and testing at a ratio of 4:1.

In addition, to further validate the hypothesis that only the 3D sub-regions within SWI images featuring the SNAS carry information relevant to prognostic prediction, we conducted experiments involving cropping 3D sub-regions of different sizes and locations, as shown in Fig. 4. All the cropped 3D sub-regions were assigned the same label, and the network was retrained accordingly.

For Review Only

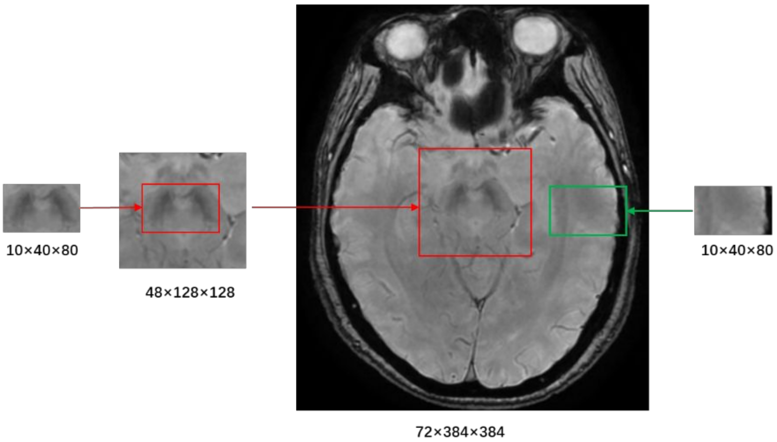

Figure 4: The 3D sub-regions extracted with different size and different location to validate whether only the 3D subregion covering the SNAS carry information relevant to prognostic prediction. The red rectangles indicate the 3D sub-regions contain the SNAS. The green rectangle indicate a 3D sub-region randomly extracted from other locations in the SWI image that does not encompass any SNAS. 10×40×80, 48×128×128, 72×384×384 denotes the size of the 3D sub-regions.

The quality of the prognostic prediction results is assessed using five metrics: Accuracy, Precision, Recall, F1 score, and AUC (Area Under the Curve). Accuracy gauges the proportion of correctly classified samples, Precision measures the proportion of true examples among samples classified as positive examples, Recall calculates the proportion of true examples among all actual positive examples. F1 Score represents the weighted harmonic average of Precision and Recall. Additionally, AUC is an indicator employed for evaluating the performance of a binary classification model.

$$Accuracy = \frac{TP + TN}{TP + FP + TN + FN}$$

$$Precision = \frac{TP}{TP + FP}$$

$$Recall = \frac{TP}{TP + FN}$$

$$F1 = \frac{2 \times Precision \times Recall}{Precision + Recall}$$

where TP is the true positive examples, TN is the true negative examples, FP is the false positive examples, FN is the false negative examples.

Statistical analysis

Demographic information and clinical details, including age, disease duration, preoperative Levodopa Challenge Test (LCT) responsiveness, pre-surgical Levodopa Equivalent Daily Dose (LEDD), and UPDRS III, were evaluated in the two groups using an independent sample t-test. Patient gender was examined using a chi-square test. The HoehnYahr Scale was analyzed using the Wilcoxon rank-sum test.

For Review Only

A significance level of $p < 0.05$ was set for a two-tailed test. The statistical analyses mentioned above were conducted using IBM SPSS Statistics Version 22.0 (International Business Machines Corporation, Armonk, NY, United States).

RESULTS

Patient characteristics

A total of 60 patients were included, all of whom underwent successful surgery. The length of STN electrical signal was recorded with an average accuracy of 5 mm during the operation. Additionally, one week after surgery, the postoperative head CT image was co-registered with preoperative T2 MRI, validating the adherence of electrode implantation to the planned trajectory. Detailed clinical data of the two groups are presented in Table 1, and there were no significant differences between the two groups in terms of age, gender, disease duration, preoperative UPDRS-III score, and the levodopa challenge test improvement rate (P >0.05).

Table 1 Demographic and clinical characteristics.

	N	Age (Mean±SD)	Gender (M or F)	Disease Duration (Years)	Hoehn-Yahr Scale (M(Q1, Q3))	LEDD (mg)	LCT Response	UPDRS-III
Optimal Group	37	57.00±8.25	22/15	8.38±2.91	3 [3, 4]	714.86±383.90	0.57±0.13	54.38±12.67
Suboptimal Group	23	60.00±6.66	11/12	8.91±3.92	3 [3, 4]	646.20±241.43	0.52±0.14	51.78±10.86
$t/\chi^2/Z$		1.47	0.776	0.605	-0.614	-0.767	-1.585	-0.814
P		0.147	0.379	0.548	0.540	0.446	0.118	0.419

*LCT, levodopa challenge test; LEDD, levodopa equivalent daily dosage. UPDRS III, Unified Parkinson's Disease Rating Scale Part III; N, the case numbers.

Prediction performance comparison of different models

In this section, we firstly analyze the quality of our prognostic prediction performance by selecting 36 cases from January 2019 to December 2021 for training and testing at a ratio of 4:1. A 3D ResNet50 is used as the baseline for the classification task. Our proposed multi-task fusion strategy outperforms others in terms of prediction accuracy, Recall and F1 score. With the complementary-task of the regression task, the final prediction results across all the evaluation metrics are improved compared to the prediction strategy that only employs use the classification branch (Table 2), and the five-fold cross-validation outcomes are detailed in Table 3 and Figure 5.

Table 2. The performance with different prediction strategy.

Method	Accuracy	Precision	Recall	F1	AUC
3D ResNet50	0.575	0.68	0.647	0.653	0.417
Classification	0.825	0.843	0.92	0.871	0.8

For Review Only

Regression	0.7	0.9	0.607	0.709	0.817
Fused	0.85	0.867	0.92	0.887	0.807

*Classification, only using the classification branch; Regression, only using the regression branch; Fused, using the multi-task fusion strategy.

Table 3 Five-fold cross-validation for the performance of the prognostic prediction using multi-task fusion strategy.

Fold	Accuracy	Precision	Recall	F1	AUC
1	0.75	0.75	1.0	0.857	0.5
2	1.0	1.0	1.0	1.0	1.0
3	1.0	1.0	1.0	1.0	1.0
4	0.625	0.75	0.6	0.667	0.8
5	0.875	0.833	1.0	0.909	0.733
Average	0.85	0.867	0.92	0.887	0.807

For Re

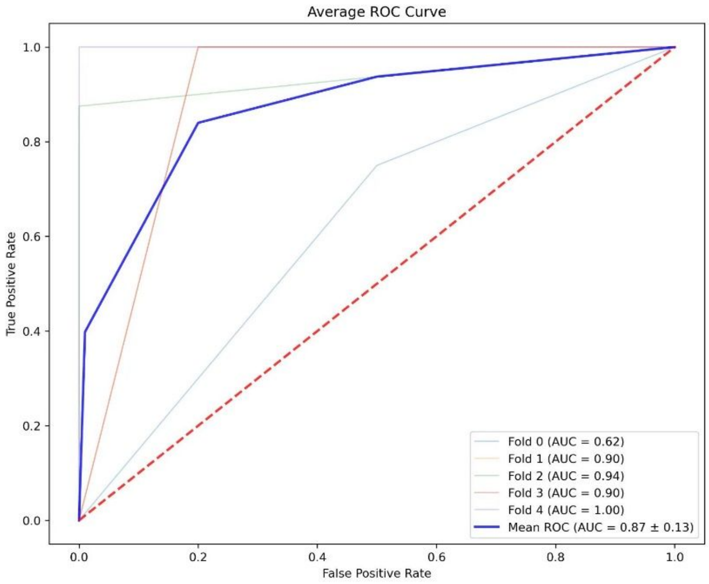

Figure 5: The ROC results of five-fold cross-validation.

We additiongally assess the multi-task prediction framework using different loss components. As show in Table 4, compared to only using the final logistic regression loss L_{log}, using the multi-task loss achieves considerable improvement, with relative improvements of 9.7% in accuracy (0.85 vs 0.775), 19.0% in recall (0.92 vs 0.773), and 8.6% in F1-score (0.887 vs 0.817).

Table 4 The performance of the multi-task prediction framework using different loss components.

Loss	Accuracy	Precision	Recall	F1	AUC

L_{log}	0.775	0.883	0.773	0.817	0.847
$L_{log} + L_{cls} + L_{reg}$	0.85	0.867	0.92	0.887	0.807

Prognostic Specificity of SNAS SWI Features

Training paradigms incorporated substantia nigra (SN)-centric subvolumes of varying dimensions (10×40×80 to 72×384×384 voxels) alongside control regions from extranigral loci. The results demonstrated SN-focused training achieved 85.3% prediction accuracy versus 62.5% for extranigral inputs, and despite uniform labeling, non-nigral 3D regions demonstrated statistically inferior performance (mean accuracy: 62.5% vs 85%, AUC: 0.37 vs 0.87) (Table 5).

Table 5. The prediction performance using the 3D sub-regions with different size and different location.

Region-Size(Pixel)	Method	Accuracy	Precision	Recall	F1	AUC
10×40×80	Fused	0.85	0.867	0.92	0.887	0.807
	3D ResNet50	0.575	0.68	0.647	0.653	0.417
48×128×128	Fused	0.725	0.725	1.0	0.833	0.673
	3D ResNet50	0.625	0.808	0.64	0.642	0.483
72×384×384	Fused	0.65	0.65	1.0	0.787	0.567
	3D ResNet50	0.625	0.693	0.813	0.734	0.6
Without SNAS	Fused	0.625	0.643	0.967	0.769	0.373
	3D ResNet50	0.65	0.671	0.88	0.757	0.613

For Review Only

Generalizability and Data Scalability of the Multi-task Prognostic Model

To investigate the generalizability of the prediction network, we increased the volume to 60 by adding 24 cases from January 2022 to December 2023, and all of them were utilized for training and testing at a ratio of 4:1. As detailed in Table 6, the multi-task fusion architecture achieved a mean accuracy of 88.3%, representing a 3.9% absolute improvement over baseline models.

Table 6 Five-fold cross-validation for the performance of the prognostic prediction using multi-task fusion strategy with 60 cases.

Fold	Accuracy	Precision	Recall	F1	AUC
1	0.666667	0.75	0.75	0.75	0.625
2	0.916667	0.875	1.0	0.933333	0.9

3	0.916667	1.0	0.875	0.933333	0.9375
4	0.916667	0.875	1.0	0.933333	0.9
5	1.0	1.0	1.0	1.0	1.0
Average	0.883333	0.9	0.925	0.91	0.8725

Furthermore, we display the feature maps before each pooling operation in the backbone, as shown in Fig. 6. The heatmaps reveal that the feature extraction process primarily emphasizes the region where the substantia nigra is located. This indicates that the features utilized for prognostic prediction predominantly originate from the SNAS.

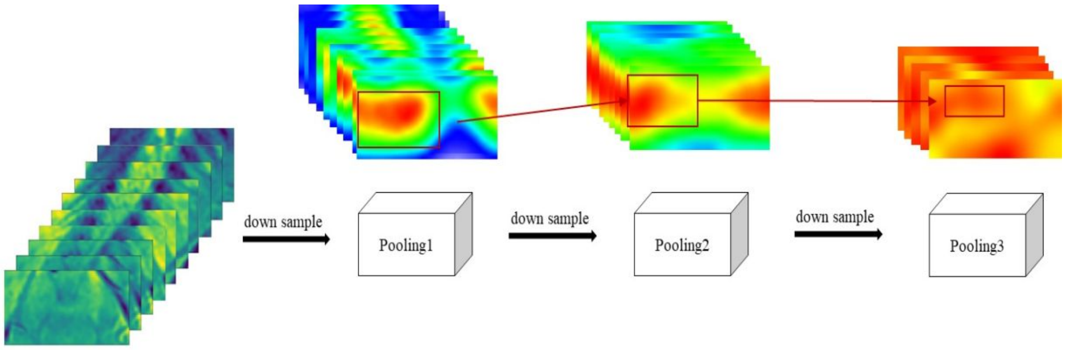

Figure 6: The feature maps before each pooling operation in the backbone.

Discussions

Even though serving as a neuroregulatory therapy with significant potential benefits for patients[13], DBS should be guided by stringent patient selection in clinical practice[14], owing to its invasiveness and heterogeneous efficacy. With increasing experience and enhanced understanding, these selection criteria have become more stringent[15], incorporating considerations such as neuropsychology, cognitive ability, and genetic characteristics[16, 17], aiming to achieve favorable outcomes for patients. Obviously, accurately predicting the efficacy of deep brain stimulation (DBS) preoperatively could better support treatment decision-making. which has been a major research focus in this field. However, the method of using LCT as a traditional clinical assessment to predict prognosis has certain limitations.

Therefore, a number of studies have explored alternative methods for prognosis prediction, including those based on clinical symptoms[18], nucleus electrophysiological signals[19], EMG signals[20], electroencephalograms[21], functional magnetic resonance imaging (fMRI) data[22], the cortical thickness of the left lateral occipital region[23], as well as prognosis prediction through Radiomics using MRI data[24]. Radiomics is a method capable of analyzing subtle features in images for clinical purposes, which has been applied to the diagnose of PD[25] by leveraging machine learning algorithms[18,24,26-29]. Liu et al.[24] employed imaging genomics in conjunction with machine learning methods, focusing on fine features of the substantia nigra and employing binary logistic regression analysis. This innovative approach demonstrates the ability to predict the prognosis outcomes of STN-DBS in improving motor symptoms of Parkinson's disease with an accuracy of 82%, a

significant improvement compared to the currently clinically used levodopa challenge test. Tsai et al[28] employed a stepwise multivariate regression model, utilizing 13 handcraft features from the preoperative diffusion tensor imaging (DTI) of PD patients, to accurately predict clinical outcomes two years after surgery. Saudargiene et al. [27] similarly employed imaging genomics in conjunction with machine learning, focusing on radiological feature analysis of the amygdala-hippocampus. Their findings suggest that changes in imaging genomics reveal the migration and deposition of pathological α-synuclein protein. A decrease in α-synuclein in the amygdala indicates advanced Parkinson's disease and a poorer prognosis for DBS treatment.

We hypothesizes that alterations in the SNAS correlate with disease status and the prognosis of DBS in PD patients, and that such alterations could be reflected by SWI image. In this study, the results showed that the SNAS in SWI image could be used to predict the improvement of motor symptoms after DBS surgery, and this also can be supported with other studies as follows. The pathology of PD is characterized by the depletion of dopamine neurons and the aggregation of alpha-synuclein in the substantia nigra[30]. Numerous studies have demonstrated that iron deposition in the substantia nigra is a contributory factor in PD, while SWI can reflect the deposition well. Ariz et al. have proved that quantification of neuromelanin and iron in Nigrosome-1 could emerge as a potential biomarker for PD by SWI image[31]. Additionally, the degree of iron deposition exhibits a positive correlation with the disease's progression[32],while the progression of iron deposition is also associated with different subtypes of Parkinson's disease[33]. Although STN represents a crucial node within the motor circuit commonly targeted for DBS, it is not a pathological nucleus for PD[34], furthermore, given that the substantia nigra functions as a node in the motor circuit and is associated with adjacent structures, in this study, SWI imaging of the SNAS was employed to predict the efficacy of STN-DBS, which aligned with our hypothesis. Our study results indicate that the accuracy of model training and prediction using the substantia nigra region is significantly higher than that using non-substantia nigra regions, suggesting that substantia nigra pathology drives this prediction accuracy. In addition, the prediction accuracy decreases when the extended substantia nigra region is used, which may be due to background interference.

In this study, patients were categorized into optimal group and suboptimal groups based on whether postoperative motor symptoms improved by more than 50%. Although most studies set the threshold at ≥ 30% referencing LCT[24, 35], however, this often results in patient dissatisfaction when the improvement in the total motor score is less than 50%. And prognosis improvement exceeding 30% was observed in the majority of patients in practice. Hence, we defined the demarcation line as 50%, which also adopted in other studies[36]. A comparative analysis of preoperative baseline data revealed no significant differences between the two groups in terms of age, disease duration, Hoehn-Yahr Scale, levodopa equivalent daily dose (LEDD), "med-off" UPDRS III score, and levodopa challenge test (LCT) improvement rate, which indicates that the two groups were statistically comparable. Notably, there was no significant distinction in the rate of LCT improvement, indicating that LCT might not be optimal for predicting motor outcome after DBS[37].

In contrast to previous studies that need to design hand-crafted features and then used the traditional machine learning model[18,24,26,29], such as random forest, to predict deep brain stimulation outcome, our approach directly employs Susceptibility Weighted Imaging (SWI) images as the input for our model in an end-to-end manner. This eliminates the need for manually designing feature

For Review Only

extraction operators. Hand-crafted features are typically carefully designed based on visual neural theories to match the data's characteristics. However, this process introduces limitations associated with human intervention. In this work, the model itself extracts features from the substantia nigra and adjacent structures, enabling better extraction of hidden features. The work that most similar with us is Matthew's study [38], which developed a convolutional neural network using the SPECT images at year 0 and year 1 to regress the UPDRS III scores at year 4. Different with it[38] , we formulate the prognostic prediction as a classification task, and propose a multi-task fusion strategy to enhance the representation ability of the neural network, the regression network in our study only acts as a complementary-task to improve the performance of the classification task. Additionally, we also used the SWI images as input and tried to predict the UPDRS III scores with a regression network, which is similar with the network used in[38], the regression results is shown in Table 2. From Table 2, it is evident that our proposed multi-task fusion strategy outperforms the regression network.

We employed a deep learning-based approach, defining the prognosis prediction task as a classification problem, and proposed a multi-task fusion network based on SWI images. By employing the concept of complementary task learning, we conducted prognosis prediction, achieving an accuracy of 88.3% (AUC = 0.873). The results indicate the presence of subtle differences in substantia nigra SWI images, which may serve as promising biomarkers associated with the DBS prognosis in PD patients. This also suggests that our model could provide neurosurgeons with a novel and practical decision-support tool, aiding in the selection of Parkinson's disease patients suitable for surgery.

This study has several limitations. Firstly, more cases need to be collected and a longer time span is required to further verify the effectiveness of the model. To mitigate potential overfitting issues arising from sample sizes, we employed a five-fold cross-validation. Secondly, the postoperative follow-up period in this study was limited to 1 year, and a more extended follow-up period would be essential to assess the long-term prognosis of patients. Additionally, non-motor symptoms and motor complications significantly impact the prognosis and patient experience after DBS, but these factors were not considered in this study. Therefore, future research should conduct long-term follow-up studies with larger sample sizes to further elucidate the prognostic predictive power of preoperative SWI of the SNAS.

In the conclusion, our study demonstrated that the SWI images covering the SNAS can be used for prognostic prediction of STN-DBS for PD. To improve the prediction performance, we proposed a deep learning based multi-task fusion network. By formulating the prognostic prediction as a classification task, and fusing the complementary-task regression learning results, our prognostic prediction accuracy achieves 88.3%, which suggests that our approach could offer a novel and practical decision-support tool for neurosurgeons and aid in screening suitable Parkinson's patients for surgery.

Declaration of Competing Interest

The authors declare that they have no known competing financial interests or personal relationships that could have appeared to influence the work reported in this paper.

Acknowledgments

This work was supported in part by the National Natural Science Foundation of China under

For Review Only

Grant 61973294, in part by the Anhui Provincial Key R&D Program under Grant (2023s07020017, 2022i01020020), in part by the niversity Synergy Innovation Program of Anhui Province, China, under Grant GXXT-2021-030, and in part by the Anhui Provincial Key Laboratory of Bionic Sensing and Advanced Robot Technology.

Author contributions

Chi Xiong: Data collection, data analysis, writing the manuscript, conceived the study and responsible for scientific integrity. Kung Wang: data analysis, writing the manuscript. Erkang Cheng: critical revision of the manuscript. Zhiyong Sun: critical revision of the manuscript. Bin Cai: data analysis, writing the manuscript, data collection, critical revision of the manuscript. Chaoshi Niu: critical revision of the manuscript, conceived the study and responsible for scientific integrity. Bo Song: critical revision of the manuscript.

For Review Only

References:

1. Lachenmayer ML, Murset M, Antih N et al. Subthalamic and pallidal deep brain stimulation for Parkinson's disease-meta-analysis of outcomes. *NPJ Parkinsons Dis*. 2021;7(1):77
2. Liang K, Li RP, Gao Y et al. Emotional symptoms and cognitive function outcomes of subthalamic stimulation in Parkinson's disease depend on location of active contacts and the volume of tissue activated. *CNS Neurosci Ther*. 2023;29(8):2355-2365
3. Geraedts VJ, Koch M, Contarino MF et al. Machine learning for automated EEG-based biomarkers of cognitive impairment during Deep Brain Stimulation screening in patients with Parkinson's Disease. *Clin Neurophysiol*. 2021;132(5):1041-1048
4. Saranza G, Lang AE. Levodopa challenge test: indications, protocol, and guide. *J Neurol*. 2021;268(9):3135-3143
5. Lin W, Shi D, Wang D et al. Can Levodopa Challenge Testing Predict the Effect of Deep Brain Stimulation? One-Year Outcomes in a Chinese Cohort. *Front Aging Neurosci*. 2021;13:764308
6. Tsafack EG, Mbiantcha M, Ateufack G et al. Antihypernociceptive and Neuroprotective Effects of the Aqueous and Methanol Stem-Bark Extracts of Nauclea pobeguinii (Rubiaceae) on STZ-Induced Diabetic Neuropathic Pain. *Evid Based Complement Alternat Med*. 2021;2021:6637584
7. Mueller K, Urgošík D, Ballarini T et al. Differential effects of deep brain stimulation and levodopa on brain activity in Parkinson's disease. *Brain Commun*. 2020;2(1):fcaa005
8. Zhao CH, Wu HT, Che HB et al. Prediction of fatal adverse prognosis in patients with fever-related diseases based on machine learning: A retrospective study. *Chin Med J (Engl)*. 2020;133(5):583-589
9. Qiu QT, Zhang J, Duan JH et al. Development and validation of radiomics model built by incorporating machine learning for identifying liver fibrosis and early-stage cirrhosis. *Chin Med J (Engl)*. 2020;133(22):2653-2659
10. Zhu X, Shi W, Ling Y et al. Contactless evaluation of rigidity in Parkinson's disease by machine vision and machine learning. *Chin Med J (Engl)*. 2023;136(18):2254-2256
11. Postuma RB, Berg D, Stern M et al. MDS clinical diagnostic criteria for Parkinson's disease. *Mov Disord*. 2015;30(12):1591-601

12. Hoops S, Nazem S, Siderowf AD et al. Validity of the MoCA and MMSE in the detection of MCI and dementia in Parkinson disease. *Neurology*. 2009;73(21):1738-45

13. Castrioto A, Lozano AM, Poon YY et al. Ten-year outcome of subthalamic stimulation in Parkinson disease: a blinded evaluation. *Arch Neurol*. 2011;68(12):1550-6

14. Defer GL, Widner H, Marie RM, Remy P, Levivier M. Core assessment program for surgical interventional therapies in Parkinson's disease (CAPSIT-PD). *Mov Disord*. 1999;14(4):572-84

15. Bronstein JM, Tagliati M, Alterman RL et al. Deep brain stimulation for Parkinson disease: an expert consensus and review of key issues. *Arch Neurol*. 2011;68(2):165

16. de Oliveira LM, Barbosa ER, Aquino CC et al. Deep Brain Stimulation in Patients With Mutations in Parkinson's Disease-Related Genes: A Systematic Review. *Mov Disord Clin Pract*. 2019;6(5):359-368

17. Tang V, Zhu XL, Lau C et al. Pre-operative cognitive burden as predictor of motor outcome following bilateral subthalamic nucleus deep brain stimulation in Parkinson's disease. *Neurol Sci*. 2022;43(12):6803-6811

18. Habets J, Janssen M, Duits AA et al. Machine learning prediction of motor response after deep brain stimulation in Parkinson's disease-proof of principle in a retrospective cohort. *PeerJ*. 2020;8:e10317

19. Ozturk M, Telkes I, Jimenez-Shahed J et al. Randomized, Double-Blind Assessment of LFP Versus SUA Guidance in STN-DBS Lead Implantation: A Pilot Study. *Front Neurosci*. 2020;14:611

20. Lagerweij S, van Zijl JC, van Laar T et al. Intermuscular coherence as a biomarker of subthalamic nucleus deep brain stimulation efficacy in Parkinson's disease. *Clin Neurophysiol*. 2022;142:36-43

21. Geraedts VJ, Koch M, Kuiper R et al. Preoperative Electroencephalography-Based Machine Learning Predicts Cognitive Deterioration After Subthalamic Deep Brain Stimulation. *Mov Disord*. 2021;36(10):2324-2334

22. Wu C, Matias C, Foltynie T et al. Dynamic Network Connectivity Reveals Markers of Response to Deep Brain Stimulation in Parkinson's Disease. *Front Hum Neurosci*. 2021;15:729677

23. Frizon LA, Gopalakrishnan R, Hogue O et al. Cortical thickness in visuo-motor areas is related to motor outcomes after STN DBS for Parkinson's disease. *Parkinsonism Relat Disord*. 2020;71:17-22

24. Liu Y, Xiao B, Zhang C et al. Predicting Motor Outcome of Subthalamic Nucleus Deep Brain Stimulation for Parkinson's Disease Using Quantitative Susceptibility Mapping and Radiomics: A Pilot Study. *Front Neurosci*. 2021;15:731109

25. Xiao B, He N, Wang Q et al. Quantitative susceptibility mapping based hybrid feature extraction for diagnosis of Parkinson's disease. *Neuroimage Clin*. 2019;24:102070

26. Hirschmann J, Steina A, Vesper J, Florin E, Schnitzler A. Neuronal oscillations predict deep brain stimulation outcome in Parkinson's disease. *Brain Stimul*. 2022;15(3):792-802

27. Saudargiene A, Radziunas A, Dainauskas JJ et al. Radiomic features of amygdala nuclei and hippocampus subfields help to predict subthalamic deep brain stimulation motor outcomes for Parkinson's disease patients. *Front Neurosci*. 2022;16:1028996

28. Tsai CC, Lin YC, Ng SH et al. A Method for the Prediction of Clinical Outcome Using Diffusion Magnetic Resonance Imaging: Application on Parkinson's Disease. *J Clin Med*. 2020;9(3)

29. Wang J, Shang R, He L et al. Prediction of Deep Brain Stimulation Outcome in Parkinson's Disease With Connectome Based on Hemispheric Asymmetry. *Front Neurosci*. 2021;15:620750

30. Hardman CD, McRitchie DA, Halliday GM, Cartwright HR, Morris JG. Substantia nigra pars

For Review Only

reticulata neurons in Parkinson's disease. *Neurodegeneration*. 1996;5(1):49-55

31. Ariz M, Martinez M, Alvarez I et al. Automatic Segmentation and Quantification of Nigrosome-1 Neuromelanin and Iron in MRI: A Candidate Biomarker for Parkinson's Disease. *J Magn Reson Imaging*. 2024;60(2):534-547
32. Yan S, Lu J, Li Y et al. Spatiotemporal patterns of brain iron-oxygen metabolism in patients with Parkinson's disease. *Eur Radiol*. 2024;34(5):3074-3083
33. Bu S, Pang H, Li X et al. Spatiotemporal nigrostriatal iron accumulation in motor subtypes of Parkinson's disease: from early to late stage. *Brain Commun*. 2025;7(6):fcaf473
34. Mazumder S, Bahar AY, Shepherd CE, Prasad AA. Post-mortem brain histological examination in the substantia nigra and subthalamic nucleus in Parkinson's disease following deep brain stimulation. *Front Neurosci*. 2022;16:948523
35. Rodriguez RL, Fernandez HH, Haq I, Okun MS. Pearls in patient selection for deep brain stimulation. *Neurologist*. 2007;13(5):253-60
36. Hayashi Y, Mishima T, Fujioka S et al. Unilateral GPi-DBS Improves Ipsilateral and Axial Motor Symptoms in Parkinson's Disease as Evidenced by a Brain Perfusion Single Photon Emission Computed Tomography Study. *Front Hum Neurosci*. 2022;16:888701
37. Schuepbach W, Tonder L, Schnitzler A et al. Quality of life predicts outcome of deep brain stimulation in early Parkinson disease. *Neurology*. 2019;92(10):e1109-e1120
38. Adams MP, Rahmim A, Tang J. Improved motor outcome prediction in Parkinson's disease applying deep learning to DaTscan SPECT images. *Comput Biol Med*. 2021;132:104312

For Review Only
