# Supplementary material for: Development and application of a multi-task fusion model using susceptibility-weighted imaging-based substantia nigra and adjacent structures for prognostic prediction of subthalamic nucleus deep brain stimulation in Parkinson’s disease
Source: Brain Commun. 2026 Sep 11;8(5):fcag333. doi: 10.1093/braincomms/fcag333 (PMC13563298; doi:10.1093/braincomms/fcag333)
Supplement: Supplementary file 1 [file fcag333_supplementary_data.zip › Revision 1 - Author's Response.pdf]

Response to the Editor and Scientific Editorial Office

Manuscript ID: BRAINCOM-2025-1391.R2

Title: Development and Application of a Multi-Task Fusion Model Using SWI-based Substantia Nigra and Adjacent Structures for Prognostic Prediction of STN-DBS in Parkinson's Disease

Dear Dr Marescotti and the Scientific Editorial Office,

Thank you for the provisional acceptance of our manuscript and for the careful editorial assessment. We have revised the manuscript, figures, figure legends, and supplementary material in accordance with all comments. A point-by-point response is provided below. All changes are shown in the tracked-changes version of the revised manuscript. We have also added a short alternative-text description under the legend of each main and supplementary figure.

Reviewer 1

Comment: The Authors have thoroughly and nicely addressed all my previous comments. I believe the article is now more robust, clear, and of possible clinically meaningfulness.

Response: We sincerely thank the reviewer for the positive assessment of our revised manuscript and for the constructive comments provided during the review process.

Comments from the Scientific Editorial Office

Comment 1: Figure 1: in the figure legend, please provide fully spelled out definitions for any abbreviations such as "SWI", "STN" and "DBS".

Response: Thank you. We revised the Figure 1 legend to provide the full definitions of subthalamic nucleus deep brain stimulation (STN-DBS), susceptibility-weighted imaging (SWI), and substantia nigra and adjacent structures (SNAS) at their first occurrence.

Comment 2: Figure 5: the combination of green and orange/red lines in this ROC curve may be difficult to interpret for colorblind readers. If possible, please reconsider the color scheme used to represent the lines in this plot. You may wish to use any colorblind-friendly palette of your choice or consider using different line styles to differentiate between certain color combinations. Please also provide a statement in the figure legend to indicate the sample size (N) upon which these analyses are based.

Response: Thank you. We revised Figure 5 using a colour-blind-friendly palette together with different line styles to distinguish the five individual cross-validation folds. The fold labels have also been standardized as Fold 1 through Fold 5,

consistent with Table 6. The mean receiver operating characteristic curve is shown as a thick blue line, the shaded band represents ±1 standard deviation across folds, and the dashed diagonal represents chance-level performance. We also added the sample size to the figure and legend: N = 60, comprising 37 optimal responders and 23 suboptimal responders.

Comment 3: Figure 6: if possible, please consider a colorblind-friendly version of these heatmaps that do not include a red-green combination. Also, if possible please include a color key/heatmap scale that indicates what the colors/shades shown in the representative heatmaps represent.

Response: Thank you. We revised Figure 6 using a colour-blind-friendly blue–gray–yellow heatmap without a red–green combination. We added a colour scale ranging from 0 to 1 and revised the legend to clarify that activation intensity was min–max normalized separately for each feature map and is displayed in arbitrary units. Blue, gray, and yellow indicate lower, intermediate, and higher responses, respectively. The feature maps are presented in the order of Pooling 1, Pooling 2, and Pooling 3.

Comment 4: Supplementary Figure 1: please spell out the abbreviation “SWI” in the figure legend.

Response: Thank you. We revised the Supplementary Figure 1 legend to define SWI as susceptibility-weighted imaging at its first occurrence.

Comment 5: Supplementary Figure 2: please spell out the abbreviations “SWI”, “SNAS” and “STN” in the figure legend.

Response: Thank you. We revised the Supplementary Figure 2 legend to provide the full definitions of susceptibility-weighted imaging (SWI), substantia nigra and adjacent structures (SNAS), and subthalamic nucleus (STN) at their first occurrence.

Comment 6: Supplementary Figure 3: please spell out the abbreviations “CT”, “MRI”, “SWI”, “DBS”, “STN” and “VTA” in the figure legend.

Response: Thank you. We revised the Supplementary Figure 3 legend to provide the full definitions of computed tomography (CT), magnetic resonance imaging (MRI), subthalamic nucleus deep brain stimulation (STN-DBS), and volume of tissue activated (VTA) at their first occurrence. We also carefully checked the revised figure and legend and confirmed that the abbreviation “SWI” does not appear in Supplementary Figure 3 and was not used to describe the image-registration or electrode-reconstruction procedure; therefore, no definition of SWI was added to this legend.

Additional accessibility requirement: Please include a short alternative-text description under the figure legend for each figure.

Response: Thank you. We added a concise alternative-text description under the legend of every main figure (Figures 1–6) and every supplementary figure (Supplementary Figures 1–3) to improve accessibility.

We appreciate the careful editorial review and believe that these revisions have improved the clarity, consistency, and accessibility of the manuscript and figures.

Sincerely,

Bo Song, Bin Cai, ChaoShi Niu

on behalf of all authors
