# Supplementary material for: Development and application of a multi-task fusion model using susceptibility-weighted imaging-based substantia nigra and adjacent structures for prognostic prediction of subthalamic nucleus deep brain stimulation in Parkinson’s disease
Source: Brain Commun. 2026 Sep 11;8(5):fcag333. doi: 10.1093/braincomms/fcag333 (PMC13563298; doi:10.1093/braincomms/fcag333)
Supplement: Supplementary file 1 [file fcag333_supplementary_data.zip › Revision 1 - Decision Letter.pdf]

Revision 1 - Decision Letter

05-Aug-2026

Dear Prof. song,

Thank you for your recent submission BRAINCOM-2025-1391.R1
entitled "Development and Application of a Multi-Task Fusion Model Using SWI-based Substantia Nigra and Adjacent Structures for Prognostic Prediction of STN-DBS in Parkinson’s Disease" to Brain Communications.

I am pleased to let you know that we would like to provisionally accept your manuscript entitled "Development and Application of a Multi-Task Fusion Model Using SWI-based Substantia Nigra and Adjacent Structures for Prognostic Prediction of STN-DBS in Parkinson’s Disease" for publication in Brain Communications. There are still some minor points for you to address before final acceptance, but further peer-review will not be necessary. However, please note that we will not accept the paper unless you comply with our policies on data analysis and presentation as noted in the comments from the Scientific Editor.

Please respond to the comments shown below from reviewers and/or our Scientific Editor and submit a revised version.

Please format your revised manuscript according to our Instructions to Authors https://academic.oup.com/braincomms/pages/General_Instructions
A summary of key points is attached to help with this. Please note that there can be substantial delays to publication if you don't resubmit in line with our requirements.

REMINDER: if you have not already done so, when submitting your revised manuscript, please include a short alternative-text description under the figure legend for each figure. Alt-text is required to ensure the accessibility of our publications. Guidance is available on the journal’s Author Guidelines webpage and at https://academic.oup.com/pages/for-authors/journals/preparing-and-submitting-your-manuscript

To revise your manuscript, click the link below or log into https://mc.manuscriptcentral.com/braincom and enter your Author Centre, where you will find your manuscript title listed under "Manuscripts with Decisions." Under "Actions," click on "Create a Revision." Your manuscript number will be appended to denote a revision.

*** PLEASE NOTE: This is a two-step process. After clicking on the link, you will be directed to a webpage to confirm. ***

https://mc.manuscriptcentral.com/braincom?URL_MASK=c9f066aa8025452b9f9592f6066f2191

Please upload a revised manuscript with the changes you have made shown either with "track changes" or in coloured text.

When submitting your revised manuscript, you will be asked to respond to the reviewer(s) and editor's comments. Please be as specific as possible in your response to all of the comments.

The editorial team at Brain Communications also aims to increase transparency in scientific publishing and to demonstrate the value that peer review brings to the scientific process. Please therefore consider whether you are willing to have reviewer comments on your paper and your response published as supplementary data with the paper if it is accepted. This is entirely optional and will only be done if both the authors and reviewers agree. When submitting your revision you will have the opportunity to update the answer you entered previously.

IMPORTANT: Your original files are available to you when you upload your revised manuscript. Please delete any redundant files before completing the submission.

Because we are trying to facilitate timely publication of manuscripts submitted to Brain Communications, we would appreciate your revised manuscript as soon as possible. If it is not possible for you to submit your revision within 3 months, the option to submit a revision will expire, and we will consider your revised paper as a new submission. However, if you need a little more time, please get in touch and we can extend this deadline for you.

Please note that Brain Communications is a fully open access journal, and if your article is accepted for publication, an open access charge will be payable (this does not apply to letters and scientific commentaries). Details can be found in our Instructions for Authors: https://academic.oup.com/braincomms/pages/General_Instructions

Once again, thank you for submitting your manuscript to Brain Communications and I look forward to receiving your revision.

Best wishes

Dr. Manuela Marescotti
Associate Editor, Brain Communications

Prof. David Belin
Editor-in-Chief Brain Communications

Reviewer(s)' Comments to Author:

Reviewer: 1 (Carlo Artusi)

Comments to the Author
The Authors have thoroughly and nicely addressed all my previous comments. I believe the article is now more robust, clear, and of possible clinically meaningfulness.

Comments from the Scientific Editorial Office:

- Figure 1: in the figure legend, please provide fully spelled out definitions for any abbreviations such as “SWI”, “STN” and “DBS”.

- Figure 5: the combination of green and orange/red lines in this ROC curve may be difficult to interpret for colorblind readers. If possible, please reconsider the color scheme used to represent the lines in this plot. You may wish to use any colorblind-friendly palette of your choice or consider using different line styles to differentiate between certain color combinations. Please also provide a statement in the figure legend to indicate the sample size (N) upon which these analyses are based.

- Figure 6: if possible, please consider a colorblind-friendly version of these heatmaps that do not include a red-green combination. Also, if possible please include a color key/heatmap scale that indicates what the colors/shades shown in the representative heatmaps represent.

- Supplementary Figure 1: please spell out the abbreviation “SWI” in the figure legend.

- Supplementary Figure 2: please spell out the abbreviations “SWI”, “SNAS” and “STN” in the figure legend.

- Supplementary Figure 3: please spell out the abbreviations “CT”, “MRI”, “SWI”, “DBS”, “STN” and “VTA” in the figure legend.
