# Supplementary material for: Development and application of a multi-task fusion model using susceptibility-weighted imaging-based substantia nigra and adjacent structures for prognostic prediction of subthalamic nucleus deep brain stimulation in Parkinson’s disease
Source: Brain Commun. 2026 Sep 11;8(5):fcag333. doi: 10.1093/braincomms/fcag333 (PMC13563298; doi:10.1093/braincomms/fcag333)
Supplement: Supplementary file 1 [file fcag333_supplementary_data.zip › Revision 1.pdf]

OXFORD UNIVERSITY PRESS | Brain Communications

Development and Application of a Multi-Task Fusion Model Using SWI-based Substantia Nigra and Adjacent Structures for Prognostic Prediction of STN-DBS in Parkinson's Disease

Journal:	*Brain Communications*
Manuscript ID	BRAINCOM-2025-1391.R1
Manuscript Type:	Original Article
Date Submitted by the Author:	02-Jun-2026

Complete List of Authors:	Xiong, Chi; Chinese Academy of Sciences Hefei Institutes of Physical Science, Institute of Intelligent Machines; The First Affiliated Hospital of USTC, Department of Neurosurgery<br>wang, kun; Chinese Academy of Sciences Hefei Institutes of Physical Science, Institute of Intelligent Machines<br>cheng, erkang; Chinese Academy of Sciences Hefei Institutes of Physical Science, Institute of Intelligent Machines<br>sun, zhiyong; Chinese Academy of Sciences Hefei Institutes of Physical Science, Institute of Intelligent Machines<br>cai, bin; Chinese Academy of Sciences Hefei Institutes of Physical Science, Institute of Intelligent Machines<br>Niu, Chaoshi; The First Affiliated Hospital of USTC, Neurosurgery<br>song, bo; Chinese Academy of Sciences Hefei Institutes of Physical Science, Institute of Intelligent Machines

Keywords:	Deep Brain Stimulation, Prognostic Prediction, Deep Learning, Susceptibility-Weighted Imaging, Parkinson's disease

SCHOLARONE MANUSCRIPTS

Development and Application of a Multi-Task Fusion Model Using SWI-based Substantia Nigra and Adjacent Structures for Prognostic Prediction of STN-DBS in Parkinson's Disease

Short title: SWI prediction of DBS outcome

Chi Xiong[1,2] , Kun Wang[1] , Erkang Cheng[1] , Zhiyong Sun[1] , Bin Cai[1] *, Chaoshi Niu[2] *, Bo Song[1]*

[1]Institute of Intelligent Machines, HFIPS, Chinese Academy of Sciences, Hefei, China

[2]Department of Neurosurgery, The First Affiliated Hospital of USTC, Division of Life Sciences and Medicine, University of Science and Technology of China, Hefei, China

Correspondence to~~：~~:

Dr~~R.~~ Chao-Shi Niu, Department of Neurosurgery, The First Affiliated Hospital of USTC, Division of Life Sciences and Medicine, University of Science and Technology of China, Hefei

230001~~，~~,China. E-m~~M~~ail: niuchaoshi@ustc.edu.cn.

Prof. Bin Cai, Institute of Intelligent Machines, HFIPS, Chinese Academy of Sciences, Hefei 230000,China. E-~~M~~mail: bcai@iim.ac.cn;

Prof. Bo Song, Institute of Intelligent Machines, HFIPS, Chinese Academy of Sciences, Hefei 230000,China. E-~~M~~mail: songbo@iim.ac.cn

For Review Only

~~[Abstract]~~ Abstract

We developed and evaluated a deep learning framework using susceptibility-weighted imaging signatures of the substantia nigra and adjacent structures to predict motor outcomes following subthalamic nucleus deep brain stimulation in Parkinson's disease. This retrospective study included patients undergoing bilateral subthalamic nucleus deep brain stimulation, and motor response was defined as the percentage improvement in the original Unified Parkinson's Disease Rating Scale Part III score from the preoperative medication-off condition to the 1-year postoperative stimulation-on/medication-off condition. Patients with at least 50% improvement were classified as optimal responders. A multi-task fusion network integrating prognostic classification and complementary regression learning was developed using preoperative susceptibility-weighted imaging data. In the initial 36-patient cohort, the proposed model outperformed baseline models and showed consistent five-fold cross-validation performance. Substantia nigra and adjacent structures-centered sub-volumes showed better prognostic performance than non-substantia nigra and adjacent structures regions. In the expanded 60-patient cohort, the model achieved 88.3% accuracy. Additional analyses showed greater 39-item Parkinson's Disease Questionnaire percentage improvement in optimal responders and no prognostic association between swallow-tail sign ratings and motor outcome. These findings suggest that preoperative susceptibility-weighted images covering the substantia nigra and adjacent structures may support prognostic prediction of subthalamic nucleus deep brain stimulation and may provide a practical decision-support tool for preoperative patient selection.~~Objective To develop and validate a deep learning framework utilizing Substantia Nigra and Adjacent Structures (SNAS) susceptibility-weighted imaging (SWI) signatures for predicting~~

motor outcomes following subthalamic nucleus deep brain stimulation (STN-DBS) in Parkinson's disease (PD) patients.

Methods This study enrolled PD patients undergoing STN-DBS, stratified into Optimal and Suboptimal groups based on whether their Unified Parkinson's Disease Rating Scale Part III (UPDRS-III) score decreased by 50% or more during the medication-off (med-off) period one year post-surgery compared to baseline. We developed a deep learning-based multi-task fusion network integrating prognostic classification with complementary regression learning to predict surgical outcomes using preoperative SWI data covering the SNAS. Initial validation (n=36 cases from January 2019 to December 2021) demonstrated superior predictive performance of our multi-task fusion network compared to baseline models. Additionally, extended SNAS and non-SNAS images were used for training to investigate the specificity of SNAS in outcome prediction. The cohort was subsequently expanded to 60 cases (including 24 additional patients from January 2022 to December 2023) to validate the model's generalizability.

Results No significant differences were observed between the two groups in terms of age, gender, disease duration, preoperative UPDRS-III score, and the levodopa challenge test improvement rate ($P > 0.05$). Our multi-task fusion model achieved 85% accuracy (vs 57.5% baseline) and 0.887 F1-score, with 9.7% accuracy improvement through multi-loss optimization. Five-fold cross-validation demonstrated consistent performance (mean AUC:0.807). SN-centric subvolumes (10×40×80 voxels) showed 85.3% prognostic accuracy versus 62.5% for extranigral regions (AUC:0.87 vs 0.37, P<0.01). Expanded validation with 60 cases enhanced generalizability (88.3% accuracy, +3.9% vs baseline).

Conclusion: Preoperative SWI images that cover the SNAS can be used for prognostic prediction of STN-DBS for PD. The proposed model offers a novel and practical decision-support tool for neurosurgeons, aiding in the identification of suitable PD patients for surgery.

[**Keywords: Key words]** Parkinson's disease; deep brain stimulation; prognostic prediction; deep learning; susceptibility-weighted imagingParkinson's disease; Deep Brain Stimulation; Prognostic Prediction; Deep Learning; Susceptibility-Weighted Imaging

For Review Only

Introduction

Parkinson's disease (PD) is a prevalent neurodegenerative condition that tends to exacerbate with the progression of its course. While pharmaceutical interventions demonstrate efficacy in the early stages, a significant number of patients necessitate Deep Brain Stimulation (DBS) to alleviate symptoms or mitigate side effects as the disease advances to an advanced stage, characterized by diminished efficacy and potential drug-related complications. The DBS has emergeds as a pivotal therapeutic intervention, supported by class I evidence, for alleviating motor symptoms associated with PD. The subthalamic nucleus (STN) is a commonly targeted area for DBS, with its electrical stimulation having been shown to significantly improve motor symptoms in patients with PD[1].[1]

Despite its recognized benefits, the efficacy of DBS surgery exhibits significant heterogeneity[2].[2] Factors such as implantation accuracy and postoperative management are acknowledged as influential contributors to outcomes. Even in experienced centers, where these factors are meticulously managed, postoperative patients still manifest vastly different outcomes, implying the involvement of undisclosed factors[3].[3] Considering the invasiveness and potential risks associated with surgery and anesthesia, along with potential side effects such as the induction of mental and cognitive symptoms through STN-DBS[3],[3]

it is important to predict surgical efficacy accurately before operation to help decide whether to perform surgery.

Current expert consensus statements and treatment guidelines for DBS in PD lack specific indications for efficacy prediction or recommendations regarding suitable prediction methods.~~In the current expert consensus and treatment guidelines for PD treatment with DBS, it lacks specific indications for efficacy prediction or recommendations of suitable prediction methods.~~ Clinically, the widely employed levodopa challenge testing (LCT) serves as a common approach for estimating efficacy~~[4]~~.[4] Beyond its role as a diagnostic tool for Parkinson's disease, LCT necessitates an improvement rate of ⩾ 30% before surgery is considered advisable. However, the predictive capacity of LCT for postoperative DBS outcome remains controversial, and recent studies have suggested that standard preoperative screening measures, including LCT, have limited ability to predict postoperative motor outcomes.[5,6]~~However, the predictive capacity of LCT for prognosis has been a subject of controversy, and recent studies generally indicate that it is a challenge task[5,6].~~ Because the mechanism of DBS in the treatment of PD differs from that of pharmacological interventions~~[7]~~,[7] it is essential to avoid confusion between the improvement rates, despite both interventions showing the capability to ameliorate patients' symptoms.

The substantia nigra is pathologically central to Parkinson's disease, with degeneration of dopaminergic neurons, α-synuclein pathology, and abnormal iron accumulation. Susceptibility-weighted imaging is sensitive to local magnetic susceptibility changes and can indirectly reflect iron-related alterations in the substantia nigra and nigrosome-1 region. Although the subthalamic nucleus is the surgical target of STN-DBS, the substantia nigra and its adjacent structures are closely connected with basal ganglia motor circuits and may contain imaging features related to disease severity, circuit integrity, and postoperative motor responsiveness. Therefore, preoperative SWI features covering the substantia nigra and adjacent structures may provide biologically plausible prognostic information for STN-DBS outcome prediction.

For Review Only

Given the inherent limitations of LCT, the pursuit of more accurate methods for preoperative prognostic prediction has become a critical focus of ongoing research in the field. Certain studies have utilized machine learning in combination with medical images to investigate strategies for prognostic prediction~~[8,9]~~[8,9] or condition evaluation~~[10]~~.[10] In this study, we developed and evaluated a deep learning framework using susceptibility-weighted imaging (SWI) signatures of the substantia nigra and adjacent structures (SNAS) to predict motor outcomes following STN-DBS in patients with PD.~~This study develops and validate a deep learning framework utilizing Substantia Nigra and Adjacent Structures (SNAS) susceptibility-weighted imaging (SWI) signatures for predicting motor outcomes following STN-DBS in PD, and the report is as follows.~~

Methods

Ethical approval

This study was approved by the Ethics Committee of the First Affiliated Hospital of USTC (Approval No.: 2022-RE-154). This retrospective study met the conditions for exemption from informed consent, and obtained the informed consent exemption from the Ethics Committee.

Study design and patients

A single-center retrospective study was conducted to develop a deep learning model to predict the

prognosis of PD patients undergoing STN-DBS. The dataset used for model development comprised 60 patients who underwent bilateral STN-DBS in the Department of Neurosurgery of the First Affiliated Hospital of USTC between January 2019 and December 2023. The dataset utilized for the development of the predictive system comprises 60 cases of bilateral STN DBS, all of them were got surgery during January 2019 to December 2023 in the Neurosurgery Department of the First Affiliated Hospital of USTC.The inclusion criteria were as follows:The admission criteria of the patient included: (1) clinically diagnosed idiopathic PD based on the 2015 MDS PD diagnostic criteria[11];[11] (2) a disease duration of more than 5 years, with the modified Hoehn-Yahr Scale between 2.5 and 4; (3) absence of severe cognitive impairment-, defined as a MoCA score ≥ 17 and/or an MMSE score ≥ 24 according to the available clinical assessment[Montreal Cognitive Assessment (MoCA) score ⩾ 17, or Mini-mental State Examination (MMSE) score ⩾ 24][12];[12] (4) with no obvious mental disorders [Hamilton Anxiety Scale (HAMA-) < 29,Hamilton Depression Scale(HAMD) < 21]; (5) no surgical contraindications; (6) no MRI contraindications; (7) willingness and ability of the patient and family members to comply with follow-up. (7) Patient and family members cooperate with follow-up.Clinical demographic and scale data were extracted from institutional preoperative assessment and postoperative follow-up records.

Treatment Protocol and Assessment of treatment response

All eligible patients received bilateral STN-DBS with microelectrode recording assistance. Non-directional Pins L301 electrodes (Pins Medical, China) were used in all patients. The first programming session was performed approximately 1 month after surgery, and subsequent stimulation parameters were adjusted according to clinical symptoms until stable efficacy was achieved.

Postoperative CT images were co-registered with preoperative MRI to evaluate lead location. Active contact coordinates and stimulation-related metrics were retrospectively reconstructed using Lead-DBS. The active contact coordinates were transformed into MNI space. Stimulation parameters, including voltage, pulse width, frequency, and contact configuration, were recorded from the chronic stimulation settings at follow-up. The volume of tissue activated (VTA), electric-field metrics, and VTA overlap with the STN and STN functional subregions were calculated for exploratory comparison between optimal and suboptimal responders.

Demographic and clinical data, including age, sex, disease duration, Hoehn-Yahr stage, levodopa equivalent daily dose (LEDD), and levodopa challenge test (LCT) responsiveness, were documented preoperatively. The original Unified Parkinson's Disease Rating Scale (UPDRS), rather than the Movement Disorder Society-sponsored revision of the UPDRS, was used in this cohort. Preoperative UPDRS-III scores were assessed in the medication-off state after withdrawal of dopamine agonists for 72 hours and other dopaminergic medications for at least 12 hours.

Postoperative motor outcome was assessed at 1 year after surgery in the stimulation-on/medication-off condition after chronic stimulation parameters had been optimized. Clinical assessments were performed by raters blinded to imaging features and model outputs. The UPDRS-III improvement rate was calculated as follows: Improvement (%) = [(preoperative UPDRS-III score − postoperative UPDRS-III score) / preoperative UPDRS-III score] × 100%. Patients with an improvement rate of at least 50% were classified as optimal responders, whereas those with an improvement rate below 50% were classified as suboptimal responders.All eligible patients received bilateral STN-DBS with microelectrode assistance. And all patients underwent their first programming session approximately one month postoperatively, then subsequent chronic parameter programming was adjusted based on the patients' symptoms until stable efficacy was achieved.

For Review Only

Demographic and clinical data, encompassing age, gender, disease duration, Hoehn-Yahr Scale, levodopa equivalent daily dose (LEDD), and Levodopa Challenge Test (LCT) responsiveness, were documented preoperatively. The baseline values for the preoperative Unified Parkinson's Disease Rating Scale Part III scores were recorded during the medication-off state(med-off), involvinga 72-hour discontinuation of dopamine agonists and a12-hour discontinuation of other drugs.

Follow-up assessments were conducted when patients' symptoms stabilized at one year postoperatively, and data on UPDRS III scores were obtained in the stimulation on (stim-on)/med-off state. Improvement (%) = [(preoperative UPDRS III score) − (postoperative UPDRS III score)] / (preoperative UPDRS III score) × 100%.

Patients were divided into optimal group and suboptimal group based on motor outcomes. Optimal outcomes were defined as improvements exceeding 50%, while suboptimal outcomes were characterized by improvements below 50%.

Additional clinical outcome analyses

To evaluate the clinical relevance of the response classification beyond UPDRS-III, additional postoperative clinical outcomes were analyzed in an exploratory manner. These included LEDD, UPDRS-IV as an available measure of motor complications, the Non-Motor Symptoms Scale (NMSS), the 39-item Parkinson's Disease Questionnaire (PDQ-39) as a disease-specific patient-reported quality-of-life measure, Mini-Mental State Examination (MMSE), Montreal Cognitive Assessment (MoCA), Hamilton Depression Rating Scale (HAMD), Hamilton Anxiety Rating Scale (HAMA), Parkinson's Disease Sleep Scale (PDSS), and constipation score. For each scale, preoperative and 1-year postoperative values were summarized, and absolute and percentage changes were calculated where applicable. For scales in which lower scores indicate better clinical status, favorable change was defined as the preoperative score minus the postoperative score; for scales in which higher scores indicate better status, favorable change was defined as the postoperative score minus the preoperative score.

Swallow-tail sign analysis

A retrospective swallow-tail sign analysis was performed on preoperative SWI images. The left and right swallow-tail signs were rated by two radiologists who were blinded to postoperative motor outcomes and model predictions, and final ratings were determined by consensus. Left, right, and total swallow-tail scores were compared between optimal and suboptimal responders. Spearman correlation analyses were performed between swallow-tail scores and continuous UPDRS-III improvement rates. Receiver operating characteristic analyses were used to evaluate the prognostic performance of swallow-tail scores for differentiating optimal from suboptimal responders.

Data acquisition and preprocessing

All patients underwent preoperative 3.0-T MRI scanning (GE MR750, USA) with a 32-channel head coil. SWI was acquired using a gradient-echo sequence with the following parameters: 104 slices; field of view, 240 mm; phase field of view, 100%; repetition time/echo time, 39/23.5 ms; flip angle, 10°; sampling bandwidth, 162.73 Hz/pixel; and acquisition time, 4 min 13 s.All patients underwent preoperative 3T MRI scanning (US GE, MR750) with a 32-channel head coil. SWI Acquisitions Gradient

For Review Only

echo sequences, using the following parameters: 104 slices, Field-of-view (FoV) = 240 mm, FoV phase 100%, Repetition time/Echo time = 39/23.5 ms; flip angle of 10°, sampling bandwidth = 162.73 Hz/pixel, Acquisition time was 4 min and 13 s for acquiring each SWI dataset.

To reduce the influence of irrelevant brain tissues, 3D sub-regions within the SWI images covering the substantia nigra and adjacent structures (SNAS) were extracted for prognostic prediction. The SNAS-centered sub-volume was designed to include the substantia nigra and adjacent deep brain structures, including part of the STN region.To mitigate the impact of other brain tissues, only the 3D sub-regions within the SWI image covering the substantia nigra and adjacent structures(SNAS) are employed for the prognostic prediction. A representative example of SNAS-centered sub-volume extraction in axial, coronal, and sagittal views is shown in Supplementary Figure 2. These 3D sub-regions were meticulously extracted by clinical surgeons from the First Affiliated Hospital of USTC as follows.

First, clinical surgeons examined the 3D SWI image slice by slice to identify the boundaries of the substantia nigra.Firstly, clinical surgeons meticulously examined the 3D SWI image slice by slice to identify the boundaries of the substantia nigra. Subsequently, a fixed-size 3D sub-volume encompassing both the left and right substantia nigra was extracted. The dimensions of this 3D sub-volume were set to 10 × 40 × 80 voxels.The dimensions of this 3D sub-volume were set at 80 pixels in length, 40 pixels in width, and 10 pixels in height. Finally, each 3D sub-volume was normalized by subtracting its mean intensity and dividing by its standard deviation to facilitate network training and improve convergence stability.Finally, to emphasize distinctions between pixel points and enhanced the network model's focus on overall image changes, facilitating smoother convergence and improved recognition rates, we normalized the 3D sub-volume by subtracting the mean value of the current pixel and dividing by the total standard deviation.

$$f(z,y,x) = \begin{cases} \dfrac{V_{z,y,x} - mean(V_{z,y,x})}{std(V_{z,y,x})}, & V_{z,y,x} > 0 \\ 0 & V_{z,y,x} = 0 \end{cases}$$

Model development

In this section, we describe the proposed multi-task fusion framework for prognostic prediction of STN-DBS motor outcome in PD. The overall architecture is shown in Fig. 1. The framework consists of four components: a backbone for 3D feature learning, a classification branch, a regression branch, and a final logistic regression classifier for fusion. The classification branch estimates the probability of optimal or suboptimal DBS motor response, whereas the regression branch predicts the continuous UPDRS-III improvement rate as a complementary task. The final classifier fuses the outputs of the classification and regression branches to generate the final prognostic prediction.In this section, our solution for prognostic prediction of STN-DBS in PD is described. The overview, of our framework for prognostic prediction of STN-DBS in PD is shown in Fig. 1. It is a multi-task fusion framework which consists of four parts, the backbone for feature learning, the regression branch, the classification branch and a classifier. The regression branch is used to regress the motor outcome improvement rate scores, the classification branch is used to give a probability value for optimal or suboptimal DBS outcome improvement. Finally, a logistic regression classifier fuses the output of the regression branch and the classification branch to predict the motor outcome of STN-DBS in PD.

For Review Only

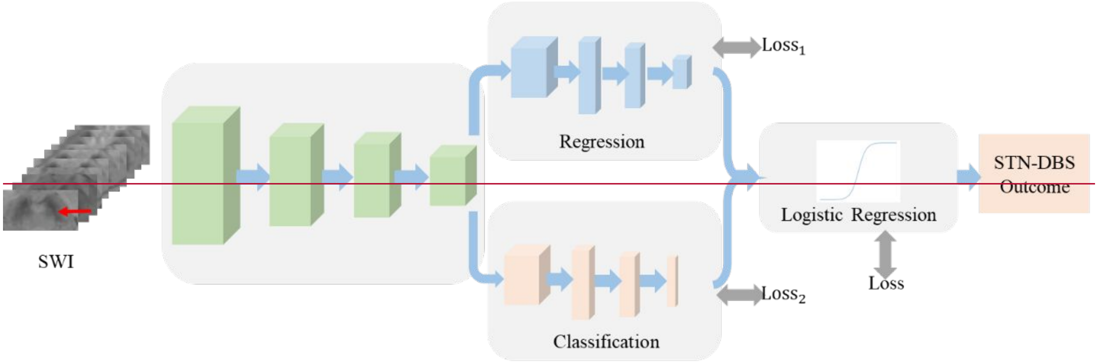

~~Figure 1: Illustration of our proposed framework for prognostic prediction of STN-DBS in PD. $Loss_1$: the regression loss, $Loss_2$: the classification loss, Loss: the logistic regression loss, Red arrow: substantia nigra.~~

We formulated prognostic prediction as a binary classification task. To improve the representation ability of the neural network and to make use of the continuous clinical outcome information, complementary-task learning was introduced. Specifically, the regression branch was used as an auxiliary task to learn the continuous motor improvement rate and to support the primary classification task.~~We formulate the prognostic prediction as a classification task, as shown in Fig. 2. To enhance the representation ability of the neural network, the idea of complementary-task learning is introduced. The details of our multi-task fusion framework for the prognostic prediction of STN-DBS in PD is shown in Fig. 3. The regression branch acts as a complementary-task to improve the performance of the classification task.~~

For Review Only

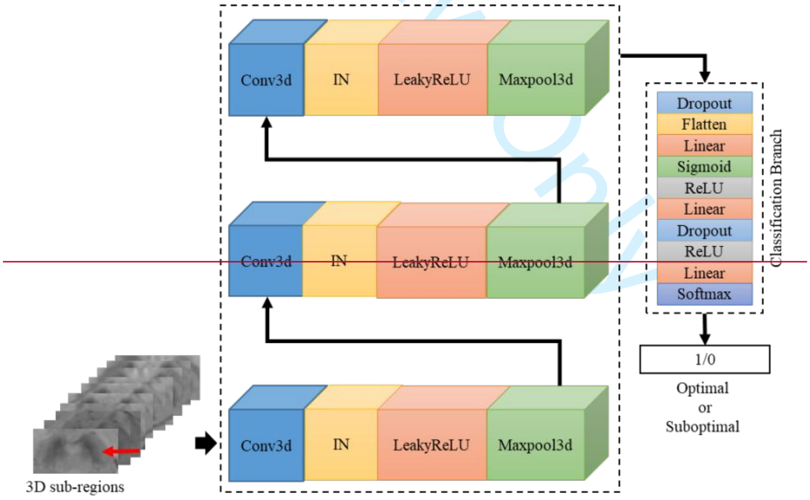

~~Figure 2: The architecture of the classification network consists of three linear convolution layers. The first two linear convolutions are followed by a Rectified Linear Unit (ReLU) operation, while the final linear convolution is followed by a softmax operation. Red arrow: substantia nigra.~~

The backbone was used for 3D feature learning. To leverage the anatomical information contained in the SNAS-centered 3D SWI sub-volume, 3D convolutional blocks were used to construct the backbone. Each 3D convolutional block consisted of 3D convolution, InstanceNorm, and LeakyReLU activation, followed by max pooling. After three down-sampling operations, the

original 3D sub-volume size was reduced from 10 × 40 × 80 to 5 × 5 × 10. The output feature maps of the backbone served as the input for both the classification and regression branches.

The classification branch was designed to classify DBS motor outcome into optimal or suboptimal response, as shown in Fig. 2. This branch consisted of three linear layers. The first two linear layers were followed by rectified linear unit activation, and the final linear layer was followed by a softmax operation to generate class probabilities.

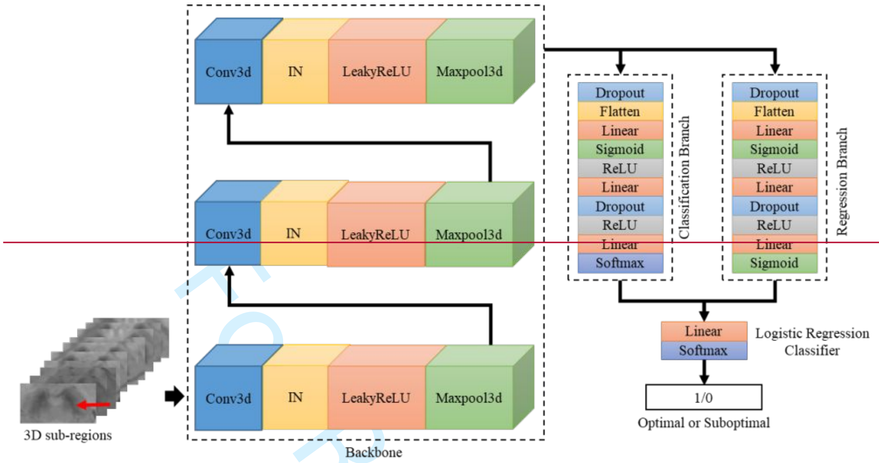

Figure 3: The structure of the proposed multi-task fusion framework

Red arrow: substantia nigra

The regression branch was designed to predict the continuous UPDRS-III improvement rate. For regression training, the UPDRS-III improvement rate was represented as a normalized proportion ranging from 0 to 1. This branch also consisted of three linear layers. The first two linear layers were followed by rectified linear unit activation, and the final linear layer was followed by a sigmoid operation to generate an output within the same 0–1 range.

The complete multi-task fusion framework, including the backbone, classification branch, regression branch, and fusion classifier, is shown in Fig. 3. In the fusion stage, a logistic regression classifier took the outputs of both the classification branch and the regression branch as input and produced the final prognostic prediction. In this way, the model incorporated both categorical response information and continuous motor improvement information during training. To effectively leverage the 3D anatomical information of the substantia nigra, we employ a 3D convolution to construct the backbone. The 3D. convolutional block in the backbone primarily consists of 3D convolution, InstanceNorm (IN), and LeakyReLU. Each 3D convolutional block is followed by a Max Pooling operation. After three down sampling operations of the Max Pooling layer, the original size of the 3D sub-volume is reduced from 10×40×80 to 5×5×10. The output feature maps of the backbone serve as the input for the multi-task learning component. The classifier aggregates the outputs of the multi-task branches and determines the final improvement effect.

The multi-task learning component comprises two parallel branches, a classification branch and a regression branch. The classification branch utilizes the output of the backbone as input and produces the probability of classifying the deep brain stimulation outcome into either optimal or suboptimal categories. To enhance the representation ability of the backbone, and act as a

For Review Only

supplement to the final prediction results, a complementary-task learning regression branch is incorporated. This branch takes the output of the backbone as input and performs regression on the improvement rate scores, as shown in Fig. 3.

Lastly, a logistic regression classifier is introduced as the fusion phase. It takes the outputs of both the classification branch and the regression branch as input and produces the final prognostic prediction.

The loss function contained three optimization objectives: the classification loss from the classification branch, the regression loss from the regression branch, and the logistic regression loss from the final fusion classifier.The loss function encompasses three optimization objectives corresponding to the tasks: two classification tasks and one regression task. For case i in the classification branch, with probability p_i and ground truth class y_i, the Cross Entropy loss is employed for the classification loss L_{cls}, and the logistic regression loss L_{log}. The SmoothL1 loss is utilized as the regression loss L_{reg} for regressing the improvement score. The final loss L_{total} is defined as:

$$L_{log} = L_{cls} = \frac{1}{N}\sum_i L_i = \frac{1}{N}\sum_i -[y_i \cdot \log(p_i) + (1-y_i) \cdot \log(1-p_i)]$$

$$L_{reg} = \begin{cases} 0.5 \cdot (y_i - p_i)^2 & if\ |y_i - p_i| < 1 \\ |y_i - p_i| - 0.5 & otherwise \end{cases}$$

$$L_{total} = \alpha \cdot L_{log} + \beta \cdot L_{cls} + \gamma \cdot L_{reg}$$

where α, β, and γ are weighting parameters for the logistic regression loss, classification loss, and regression loss, respectively.where α , β and γ are balancing parameters between the logistic regression loss ,the classification loss and the regression loss.

Implementation details and performance evaluationImplementation details and Evaluation Indicators

A five-fold cross-validation strategy was used to evaluate model performance. In each fold, the dataset was split at the patient level into training and testing subsets with a ratio of 4:1, ensuring that images from the same patient did not appear in both training and testing subsets. Data augmentation was applied only to the training data in an on-the-fly manner, including random elastic deformation, random rotation, random scaling, and random mirroring. The network was optimized using the Adam optimizer with an initial learning rate of 0.01 and a weight decay of 1 × 10−4.

Because the proposed framework used a multi-task learning strategy, the classification and regression tasks could mutually reinforce but also compete with each other during optimization. To reduce potential task imbalance, the weighting parameters α, β, and γ were dynamically adjusted during training.

To evaluate data scalability within this single-center retrospective cohort, we first trained and tested the model using 36 cases collected from January 2019 to December 2021. Subsequently, the dataset was expanded to 60 cases by adding 24 patients collected from January 2022 to December 2023, and the same five-fold cross-validation strategy was applied to the expanded cohort. Because no independent external cohort was available, the expanded 60-patient analysis should be interpreted as an internal scalability analysis rather than external validation. A five-fold cross-validation is used to investigate the prediction accuracy and the generalizability of the prediction

For Review Only

network. We randomly split the cases for training and testing with a ratio of 4:1. We used the data augmentation strategies during training, including random elastic deformation, random rotation, random scaling and random mirroring. The data augmentation is performed "on-the-fly" during training. The network is optimized by Adam with an initial learning rate 0.01, and weight decay of 1e-4. Given that the proposed method employs a multi-task training strategy, the tasks mutually reinforce and compete with each other. To mitigate potential conflicts arising from competition, the balancing parameters α, β, and γ are dynamically adjusted to different values as the number of training epochs increases. To investigate the generalizability of the prediction network, we first selected 36 cases from January 2019 to December 2021 for training and testing at a ratio of 4:1. Subsequently, we increased the volume to 60 by adding 24 cases from January 2022 to December 2023, and all of them were utilized for training and testing at a ratio of 4:1.

To further examine whether SNAS-centered SWI sub-regions contained prognostic information, additional experiments were performed using 3D sub-regions of different sizes and locations, as shown in Fig. 4. These included SNAS-centered sub-volumes with different spatial extents and control sub-volumes extracted from non-SNAS regions. All sub-regions were assigned the same patient-level outcome labels, and the network was retrained using the same training and evaluation strategy.In addition, to further validate the hypothesis that only the 3D sub-regions within SWI images featuring the SNAS carry information relevant to prognostic prediction, we conducted experiments involving cropping 3D sub-regions of different sizes and locations, as shown in Fig. 4. All the cropped 3D sub-regions were assigned the same label, and the network was retrained accordingly.

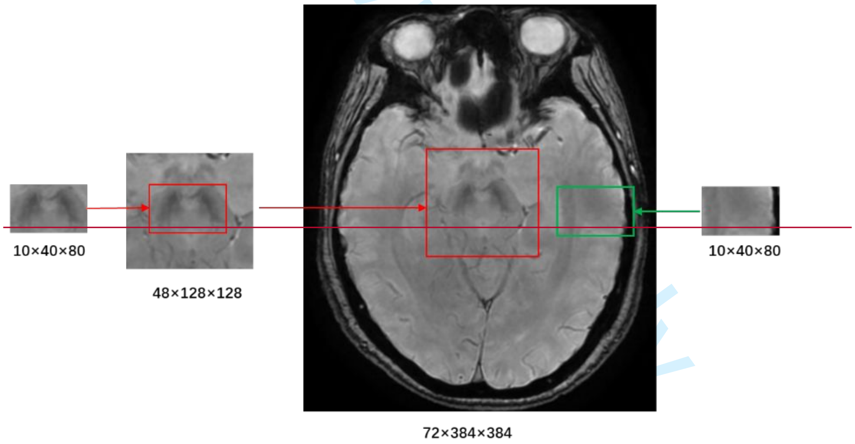

Figure 4: The 3D sub-regions extracted with different size and different location to validate whether only the 3D subregion covering the SNAS carry information relevant to prognostic prediction. The red rectangles indicate the 3D sub-regions contain the SNAS. The green rectangle indicate a 3D sub-region randomly extracted from other locations in the SWI image that does not encompass any SNAS. 10×40×80, 48×128×128, 72×384×384 denotes the size of the 3D sub-regions.

The prognostic prediction performance was evaluated using accuracy, precision, recall, F1 score, and area under the receiver operating characteristic curve (AUC). Accuracy was defined as the proportion of correctly classified samples. Precision was defined as the proportion of true positives among samples predicted as positive. Recall was defined as the proportion of true positives among all actual positive samples. The F1 score was calculated as the harmonic mean of precision and recall. AUC was used to evaluate the discrimination ability of the binary classification model.

The quality of the prognostic prediction results is assessed using five metrics: Accuracy, Precision, Recall, F1 score, and AUC (Area Under the Curve). Accuracy gauges the proportion of correctly classified samples, Precision measures the proportion of true examples among samples classified as positive examples, Recall calculates the proportion of true examples among all actual positive examples. F1 Score represents the weighted harmonic average of Precision and Recall. Additionally, AUC is an indicator employed for evaluating the performance of a binary classification model.

$$Accuracy = \frac{TP + TN}{TP + FP + TN + FN}$$

$$Precision = \frac{TP}{TP + FP}$$

$$Recall = \frac{TP}{TP + FN}$$

$$F1 = \frac{2 \times Precision \times Recall}{Precision + Recall}$$

where TP, TN, FP, and FN denote true positives, true negatives, false positives, and false negatives, respectively.where TP is the true positive examples, TN is the true negative examples, FP is the false positive examples, FN is the false negative examples.

Baseline model

Because the primary prognostic prediction task was formulated as a binary classification problem, a 3D ResNet50 model was used as the baseline classification model. The same SNAS-centered 3D SWI sub-volumes were used as input for the baseline model. The baseline model was trained and evaluated using the same patient-level data splitting strategy, five-fold cross-validation procedure, data augmentation strategy, and performance metrics as the proposed multi-task fusion framework. The architecture of the 3D ResNet50 baseline model is shown in Supplementary Figure 1.

Statistical analysis

Continuous variables were summarized as mean ± standard deviation and median with interquartile range, and categorical variables were summarized as counts and percentages. Between-group comparisons of continuous clinical variables between optimal and suboptimal responders were performed using the Mann–Whitney U test because of the modest sample size and potential non-normal distribution of several clinical scales. Categorical variables were compared using Fisher's exact test or the chi-square test, as appropriate. Rank-biserial correlation was reported as an effect-size measure for Mann–Whitney U tests.

For additional clinical outcome analyses, preoperative values, postoperative values, absolute changes, favorable changes, and percentage favorable changes were compared between groups. Spearman correlation was used to assess associations between swallow-tail scores and continuous

For Review Only

UPDRS-III improvement rates. Receiver operating characteristic analysis was used to evaluate the ability of swallow-tail scores to discriminate optimal from suboptimal responders. Benjamini–Hochberg false discovery rate correction was applied for exploratory multiple comparisons. DBS stimulation parameters, active contact coordinates, VTA metrics, and VTA-STN overlap variables were compared between groups using Mann–Whitney U tests and were interpreted as exploratory analyses. A two-sided P value < 0.05 was considered nominally significant. Statistical analyses were performed using Python, including pandas, scipy, and scikit-learn.Demographic information and clinical details, including age, disease duration, preoperative Levodopa Challenge Test (LCT) responsiveness, pre-surgical Levodopa Equivalent Daily Dose (LEDD), and UPDRS III, were evaluated in the two groups using an independent sample t-test. Patient gender was examined using a chi-square test. The HoehnYahr Scale was analyzed using the Wilcoxon rank-sum test. A significance level of $p < 0.05$ was set for a two-tailed test. The statistical analyses mentioned above were conducted using IBM SPSS Statistics Version 22.0 (International Business Machines Corporation, Armonk, NY, United States).

RESULTSResults

Patient characteristics

A total of 60 patients were included, all of whom underwent successful surgery. The length of STN electrical signal was recorded with an average accuracy of 5 mm during the operation. Postoperative CT-MRI fusion was performed for routine assessment of lead location, and Lead-DBS-based reconstruction was available for exploratory electrode localization and stimulation-related analyses.Additionally, one week after surgery, the postoperative head CT image was co-registered with preoperative T2 MRI, validating the adherence of electrode implantation to the planned trajectory. Core demographic and baseline clinical characteristics are presented in Table 1, and detailed baseline clinical variables are provided in Supplementary Table 1. The optimal responder group included 37 patients, and the suboptimal responder group included 23 patients. Baseline demographic and clinical characteristics were generally comparable between groups, including age, sex, disease duration, age at onset, preoperative Hoehn-Yahr stage, LEDD, preoperative UPDRS-III, UPDRS-IV, LCT responsiveness, NMSS, PDQ-39, MMSE, MoCA, HAMD, HAMA, PDSS, and constipation score, with no nominally significant between-group differences.Detailed clinical data of the two groups are presented in Table 1, and there were no significant differences between the two groups in terms of age, gender, disease duration, preoperative UPDRS-III score, and the levodopa challenge test improvement rate (*P* >0.05).

Table 1. Demographic and clinical characteristics.

Variable	Optimal Group	Suboptimal Group	P
N	37	23	
Age, years	56.19 ± 6.51	60.00 ± 6.66	0.072
Sex, M/F	22/15	11/12	0.431
Disease duration, years	8.38 ± 2.91	8.91 ± 3.92	0.878
Hoehn-Yahr Scale, median [IQR]	3 [3, 4]	3 [3, 4]	0.545

For Review Only

Table 1. Demographic and clinical characteristics.

Variable	Optimal Group	Suboptimal Group	P
LEDD, mg	714.86 ± 383.90	646.20 ± 241.43	0.825
LCT response	0.57 ± 0.13	0.52 ± 0.14	0.146
UPDRS-III	54.38 ± 12.67	51.78 ± 10.86	0.420

*LCT, levodopa challenge test; LEDD, levodopa equivalent daily dosage. UPDRS-III, Unified Parkinson's Disease Rating Scale Part III; N, number of patients.

*LCT, levodopa challenge test; LEDD, levodopa equivalent daily dosage. UPDRS III, Unified Parkinson's Disease Rating Scale Part III; N, the case numbers.

Additional clinical outcomes beyond UPDRS-III

Additional postoperative clinical outcomes are summarized in Supplementary Table 2. Optimal responders showed greater improvement in quality of life as measured by PDQ-39. Postoperative PDQ-39 scores were lower in optimal responders than in suboptimal responders (37.95 ± 22.30 vs 56.13 ± 20.30, nominal P = 0.005). The percentage favorable change in PDQ-39 was also greater in optimal responders (39.19 ± 31.09% vs 13.75 ± 28.05%, nominal P = 0.003) and remained significant after false discovery rate correction (q = 0.031). The absolute favorable change in PDQ-39 was nominally greater in optimal responders (24.35 ± 21.58 vs 10.96 ± 19.05, nominal P = 0.011), although it did not remain significant after false discovery rate correction (q = 0.106). Changes in LEDD, UPDRS-IV, NMSS, MMSE, MoCA, HAMD, HAMA, PDSS, and constipation score did not significantly differ between groups.

Individual UPDRS-III values and continuous improvement rates

Individual preoperative UPDRS-III scores, 1-year postoperative stimulation-on/medication-off UPDRS-III scores, continuous UPDRS-III improvement rates, and binary response classifications are provided in Supplementary Table 3. The continuous UPDRS-III improvement rate was used by the regression branch of the proposed multi-task framework as complementary learning information, whereas the binary responder label was used for the primary prognostic classification task.

Swallow-tail sign analysis

Swallow-tail sign analyses are summarized in Supplementary Table 4. Left, right, and total swallow-tail scores did not significantly differ between optimal and suboptimal responders. The total swallow-tail score was 1.81 ± 0.52 in optimal responders and 1.74 ± 0.54 in suboptimal responders (P = 0.453). Swallow-tail scores were not significantly correlated with continuous UPDRS-III improvement rates (total score: Spearman's rho = 0.030, P = 0.817). The prognostic performance of swallow-tail scores was close to chance level, with AUC values of 0.503 for the left score, 0.533 for the right score, and 0.538 for the total score.

For Review Only

DBS electrode localization and stimulation-related analyses

All patients were implanted with bilateral non-directional Pins L301 electrodes. Postoperative CT-MRI fusion and Lead-DBS-based reconstruction were used to evaluate active contact coordinates and stimulation-related metrics. A group-level Lead-DBS reconstruction of bilateral electrode trajectories and active contacts relative to the STN region is shown in Supplementary Figure 3. Active contact coordinates in MNI space are summarized in Supplementary Table 5. No significant between-group differences were observed in the left or right active contact coordinates along the x, y, or z axes.

Stimulation parameters and VTA-related metrics are summarized in Supplementary Table 6, and patient-level DBS stimulation parameters, active contact coordinates, VTA-related metrics, and VTA-STN overlap values are provided in Supplementary Table 7. The total bilateral TEED was 114.43 ± 69.23 μ J/s in optimal responders and 133.58 ± 65.74 μ J/s in suboptimal responders, with no significant between-group difference ($P = 0.176$). The mean bilateral VTA volume was numerically larger in suboptimal responders than in optimal responders (110.75 ± 50.31 vs. 83.70 ± 43.94 mm^3; nominal $P = 0.040$), but this finding did not remain significant after false discovery rate correction.

Exploratory VTA-STN overlap analyses showed that the total bilateral VTA-STN overlap was numerically larger in suboptimal responders than in optimal responders (190.69 ± 88.32 vs. 144.28 ± 72.84 mm^3; nominal $P = 0.046$). The total bilateral VTA overlap with the motor STN subregion was also larger in suboptimal responders (46.65 ± 21.97 vs. 32.49 ± 20.65 mm^3; nominal $P = 0.015$). However, none of these VTA-related differences remained significant after false discovery rate correction. These exploratory findings suggest that stimulation-related factors may also contribute to postoperative motor outcome and should be incorporated into future multimodal prediction models.

Prediction performance comparison of different models

We first evaluated prognostic prediction performance using the initial 36-patient cohort collected from January 2019 to December 2021. A 3D ResNet50 model was used as the baseline classification model and was trained and tested using the same SNAS-centered inputs and five-fold cross-validation strategy. As shown in Table 2, the proposed multi-task fusion strategy outperformed the 3D ResNet50 baseline and the single-task branches in terms of accuracy, recall, F1 score, and AUC. Compared with the classification-only branch, the fused model achieved higher overall performance, suggesting that complementary regression learning using the continuous UPDRS-III improvement rate contributed additional prognostic information. The five-fold cross-validation results are shown in Table 3. ~~In this section, we firstly analyze the quality of our prognostic prediction performance by selecting 36 cases from January 2019 to December 2021 for training and testing at a ratio of 4:1. A 3D ResNet50 is used as the baseline for the classification task. Our proposed multi-task fusion strategy outperforms others in terms of prediction accuracy, Recall and F1 score. With the complementary-task of the regression task, the final prediction results across all the evaluation metrics are improved compared to the prediction strategy that only employs~~

For Review Only

~~use the classification branch (Table 2), and the five-fold cross-validation outcomes are detailed in Table 3 and Figure 5.~~

Table 2. The performance with different prediction strategy.

Method	Accuracy	Precision	Recall	F1	AUC
3D ResNet50	0.575	0.68	0.647	0.653	0.417
Classification	0.825	0.843	0.92	0.871	0.8
Regression	0.7	0.9	0.607	0.709	0.817
Fused	0.85	0.867	0.92	0.887	0.807

*Classification, only using the classification branch; Regression, only using the regression branch; Fused, using the multi-task fusion strategy.

Table 3. Five-fold cross-validation for the performance of the prognostic prediction using multi-task fusion strategy.

Fold	Accuracy	Precision	Recall	F1	AUC
1	0.75	0.75	1.0	0.857	0.5
2	1.0	1.0	1.0	1.0	1.0
3	1.0	1.0	1.0	1.0	1.0
4	0.625	0.75	0.6	0.667	0.8
5	0.875	0.833	1.0	0.909	0.733
Average	0.85	0.867	0.92	0.887	0.807

For Review O

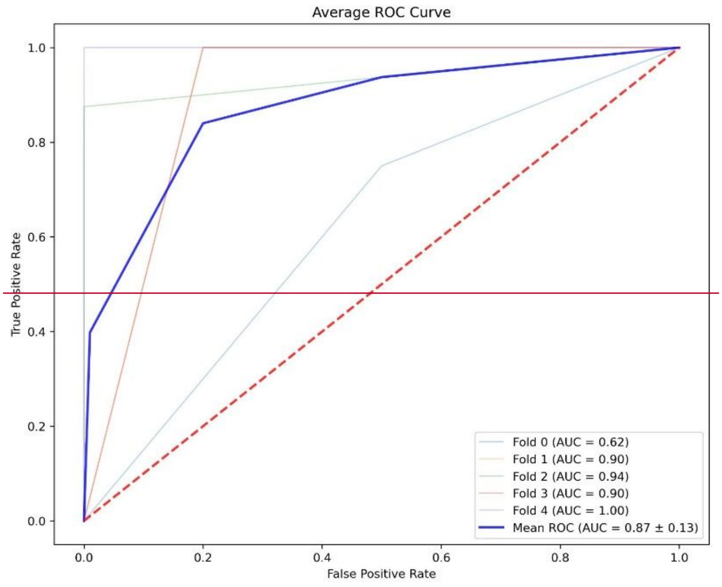

~~Figure 5: The ROC results of five-fold cross-validation.~~

~~We additiongally assess the multi-task prediction framework using different loss components. As show in Table 4, compared to only using the final logistic regression loss L_{log}, using the multi-task loss achieves considerable improvement, with relative improvements of 9.7% in accuracy (0.85 vs 0.775), 19.0% in recall (0.92 vs 0.773), and 8.6% in F1-score (0.887 vs 0.817).~~ We additionally assessed the multi-task prediction framework using different loss components. As shown in Table 4, compared with using only the final logistic regression loss, the multi-task loss achieved improved performance, with absolute improvements in accuracy (0.850 vs 0.775), recall (0.920 vs 0.773), and F1 score (0.887 vs 0.817).

Table 4. The performance of the multi-task prediction framework using different loss components.

Loss	Accuracy	Precision	Recall	F1	AUC
L_{log}	0.775	0.883	0.773	0.817	0.847
$L_{log} + L_{cls} + L_{reg}$	0.85	0.867	0.92	0.887	0.807

Prognostic Specificity of SNAS SWI Features

To examine the anatomical specificity of the SWI-based prognostic features, we compared SNAS-centered sub-volumes of different sizes with control sub-volumes extracted from non-SNAS regions. As shown in Table 5, the SNAS-centered 10 × 40 × 80 sub-volume achieved better prognostic performance than larger sub-volumes and non-SNAS control regions. In contrast, non-SNAS regions showed substantially lower AUC values, suggesting that prognostically relevant information appeared to be concentrated around the SNAS rather than being driven by nonspecific whole-image features.~~Training paradigms incorporated substantia nigra (SN)-centric subvolumes of varying dimensions (10 × 40 × 80 to 72 × 384 × 384 voxels) alongside control regions from extranigral loci. The results demonstrated SN-focused training achieved 85.3% prediction accuracy versus 62.5% for extranigral inputs, and despite uniform labeling, non-nigral 3D regions demonstrated statistically inferior performance (mean accuracy: 62.5% vs 85%, AUC: 0.37 vs 0.87) (Table 5).~~

Table 5. The prediction performance using the 3D sub-regions with different size and different location.

Region-Size(Pixel)	Method	Accuracy	Precision	Recall	F1	AUC
10×40×80	Fused	0.85	0.867	0.92	0.887	0.807
	3D ResNet50	0.575	0.68	0.647	0.653	0.417
48×128×128	Fused	0.725	0.725	1.0	0.833	0.673
	3D ResNet50	0.625	0.808	0.64	0.642	0.483
	Fused	0.65	0.65	1.0	0.787	0.567

For Review Only

72×384×384	3D ResNet50	0.625	0.693	0.813	0.734	0.6
non-SNAS regions~~Without SNAS~~	Fused	0.625	0.643	0.967	0.769	0.373
	3D ResNet50	0.65	0.671	0.88	0.757	0.613

Internal scalability analysis of the multi-task prognostic model~~Generalizability and Data Scalability of the Multi-task Prognostic Model~~

To evaluate the internal scalability of the prediction network, the dataset was expanded to 60 patients by adding 24 cases collected from January 2022 to December 2023. The same five-fold patient-level cross-validation strategy was applied to the expanded cohort. As detailed in Table 6, the multi-task fusion architecture achieved a mean accuracy of 88.3% and a mean AUC of 0.873 in the expanded cohort. Because this analysis was performed within a single-center retrospective dataset, it should be interpreted as an internal scalability analysis rather than external validation.~~To investigate the generalizability of the prediction network, we increased the volume to 60 by adding 24 cases from January 2022 to December 2023, and all of them were utilized for training and testing at a ratio of 4:1. As detailed in Table 6, the multi-task fusion architecture achieved a mean accuracy of 88.3%, representing a 3.9% absolute improvement over baseline models.~~

For Review Only

Table 6. Five-fold cross-validation for the performance of the prognostic prediction using multi-task fusion strategy with 60 cases.

Fold	Accuracy	Precision	Recall	F1	AUC
1	0.666667	0.75	0.75	0.75	0.625
2	0.916667	0.875	1.0	0.933333	0.9
3	0.916667	1.0	0.875	0.933333	0.9375
4	0.916667	0.875	1.0	0.933333	0.9
5	1.0	1.0	1.0	1.0	1.0
Average	0.883333	0.9	0.925	0.91	0.8725

The ROC curves of the five-fold cross-validation in the expanded 60-patient cohort are shown

in Fig. 5.

Furthermore, feature maps before each pooling operation in the backbone are displayed in Fig. 6. The heatmaps showed that the feature extraction process primarily emphasized the region where the substantia nigra is located, supporting that the features used for prognostic prediction were mainly derived from the SNAS-centered sub-volume.Furthermore, we display the feature maps before each pooling operation in the backbone, as shown in Fig. 6. The heatmaps reveal that the feature extraction process primarily emphasizes the region where the substantia nigra is located. This indicates that the features utilized for prognostic prediction predominantly originate from the SNAS.

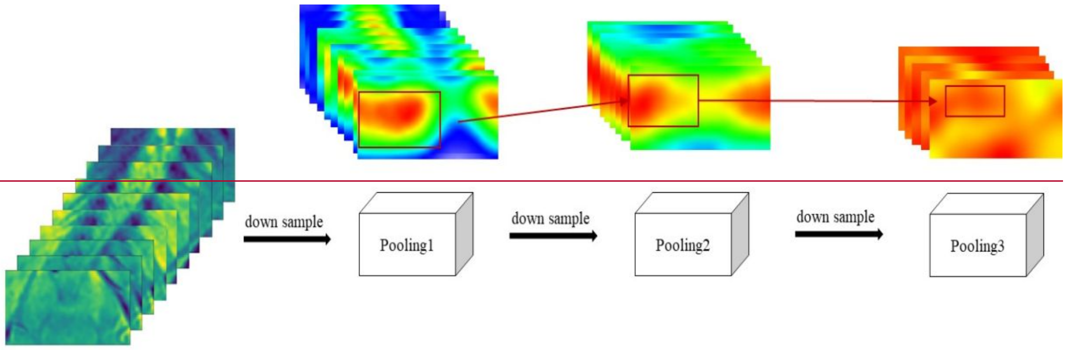

Figure 6: The feature maps before each pooling operation in the backbone.

Discussions

Even though serving as a neuroregulatory therapy with significant potential benefits for patients[13],[13] DBS should be guided by stringent patient selection in clinical practice[14],[14] owing to its invasiveness and heterogeneous efficacy. With increasing experience and enhanced understanding, these selection criteria have become more stringent[15],[15] incorporating considerations such as neuropsychology, cognitive ability, and genetic characteristics[[16, 17]],,[16,17] with the aim ofaiming to achievinge favorable outcomes for patients. Accurate preoperative prediction of deep brain stimulation (DBS) efficacy could better support treatment decision-making, and this has been a major research focus in the field. Obviously, accurately predicting the efficacy of deep brain stimulation (DBS) preoperatively could better support treatment decision-making. which has been a major research focus in this field. However, the method of using LCT as a traditional clinical assessment to predict prognosis has certain limitations.

Therefore, a number of studies have explored alternative methods for prognosis prediction, including those based on clinical symptoms[18],[18] nucleus electrophysiological signals[19],[19] EMG signals[20],[20] electroencephalograms[21],[21] functional magnetic resonance imaging (fMRI) data,a[22],[22] the cortical thickness of the left lateral occipital region[23],[23] as well as prognosis prediction through Radiomics using MRI data[24].[24] Radiomics is a method for extracting subtle quantitative features from medical images for clinical applications and has been applied to the diagnosis of PD[25] and prognostic prediction by leveraging machine learning algorithms.[18,24,26-29] Radiomics is a method capable of analyzing subtle features in images for clinical purposes, which has been applied to the diagnose of PD[25] by leveraging machine learning algorithms[18,24,26-29]. Liu et al.[2424] employed

imaging genomics in conjunction with machine learning methods, focusing on fine features of the substantia nigra and employing binary logistic regression analysis. This innovative approach demonstrates the ability to predict the prognosis outcomes of STN-DBS in improving motor symptoms of Parkinson's disease with an accuracy of 82%, a significant improvement compared to the currently clinically used levodopa challenge test. Tsai et al.[28][28] employed a stepwise multivariate regression model, utilizing 13 handcrafted features from the preoperative diffusion tensor imaging (DTI) of PD patients, to accurately predict clinical outcomes two years after surgery. Saudargiene et al.[27][27] similarly employed imaging genomics in conjunction with machine learning, focusing on radiological feature analysis of the amygdala-hippocampus. Their findings suggest that changes in imaging genomics reveal the migration and deposition of pathological α-synuclein protein. A decrease in α-synuclein in the amygdala indicates advanced Parkinson's disease and a poorer prognosis for DBS treatment.

We hypothesized that alterations in the SNAS may correlate with disease status and DBS prognosis in patients with PD, and that such alterations could be reflected on SWI images. We hypothesizes that alterations in the SNAS correlate with disease status and the prognosis of DBS in PD patients, and that such alterations could be reflected by SWI image. In this study, the results showed that SNAS-centered SWI images could be used to predict motor improvement after DBS surgery, which is supported by previous studies.In this study, the results showed that the SNAS in SWI image could be used to predict the improvement of motor symptoms after DBS surgery, and this also can be supported with other studies as follows. The pathology of PD is characterized by the depletion of dopamine neurons and the aggregation of alpha-synuclein in the substantia nigra[30].[30] Numerous studies have demonstrated that iron deposition in the substantia nigra is a contributory factor in PD, while SWI can reflect the deposition well. Ariz et al. have proved that quantification of neuromelanin and iron in Nigrosome-1 could emerge as a potential biomarker for PD by SWI image[31].[31] Additionally, the degree of iron deposition exhibits a positive correlation with the disease's progression[32],[32] while the progression of iron deposition is also associated with different subtypes of Parkinson's disease[33].[33] Although the STN represents a crucial node within the motor circuit and is commonly targeted for DBS, it is not the primary pathological nucleus in PD.[34] Given that the substantia nigra is closely related to basal ganglia motor circuits and adjacent deep brain structures, we used SWI features of the SNAS to predict the efficacy of STN-DBS, which was consistent with our hypothesisAlthough STN represents a crucial node within the motor circuit commonly targeted for DBS, it is not a pathological nucleus for PD[34], furthermore, given that the substantia nigra functions as a node in the motor circuit and is associated with adjacent structures, in this study, SWI imaging of the SNAS was employed to predict the efficacy of STN-DBS, which aligned with our hypothesis. Our study results indicate that the prediction performance using SNAS-centered regions was higher than that using non-SNAS region accuracy of model training and prediction using the substantia nigra region is significantly higher than that using non-substantia nigra regions, suggesting that prognostically relevant information may be enriched in the substantia nigra and adjacent structuressuggesting that substantia nigra pathology drives this prediction accuracy. In addition, the prediction accuracy decreases when the extended substantia nigra region is used, which may be due to background interference.

In this study, patients were categorized into optimal and suboptimal groups based on whether postoperative motor symptoms improved by more than 50%. We acknowledge that dichotomizing a continuous motor improvement measure may lead to loss of information. To partly address this

For Review Only

issue, individual continuous UPDRS-III improvement rates are now provided, and the regression branch of the proposed multi-task framework incorporated the continuous improvement rate as complementary learning information. Nevertheless, responder classification was retained as the primary endpoint because it provides an intuitive and clinically interpretable threshold for identifying patients with suboptimal motor benefit after STN-DBS. Although most studies have set the threshold at $\geq$30% with reference to the levodopa challenge test,[24,35] improvement of less than 50% in the total motor score often results in patient dissatisfaction. Moreover, improvement exceeding 30% was observed in the majority of patients in clinical practice. Hence, we defined the demarcation threshold as 50%, which has also been adopted in previous studies.[36] A comparative analysis of preoperative baseline data revealed no significant between-group differences in core demographic and clinical characteristics, including age, sex, disease duration, Hoehn-Yahr stage, LEDD, medication-off UPDRS-III score, and LCT improvement rate, indicating that the two groups were statistically comparable. Notably, there was no significant difference in the LCT improvement rate, consistent with previous studies suggesting that LCT alone may not optimally predict motor outcome after DBS.[5,6] Quality of life has also been reported to be associated with DBS outcome, further supporting the clinical relevance of preoperative prognostic assessment.[37]

~~In this study, patients were categorized into optimal group and suboptimal groups based on whether postoperative motor symptoms improved by more than 50%. Although most studies set the threshold at ≥ 30% referencing LCT[24, 35], however, this often results in patient dissatisfaction when the improvement in the total motor score is less than 50%. And prognosis improvement exceeding 30% was observed in the majority of patients in practice. Hence, we defined the demarcation line as 50%, which also adopted in other studies[36]. A comparative analysis of preoperative baseline data revealed no significant differences between the two groups in terms of age, disease duration, Hoehn-Yahr Scale, levodopa equivalent daily dose (LEDD), "med-off" UPDRS III score, and levodopa challenge test (LCT) improvement rate, which indicates that the two groups were statistically comparable. Notably, there was no significant distinction in the rate of LCT improvement, indicating that LCT might not be optimal for predicting motor outcome after DBS[37].~~

In contrast to previous studies that relied on handcrafted feature design followed by conventional machine learning models such as random forest,[18,24,26,29] our approach directly used SWI images as model input in an end-to-end manner. This avoided manual feature engineering and allowed the network to learn latent prognostic features directly from the substantia nigra and adjacent structures. Adams et al.[38] developed a convolutional neural network using DaTscan SPECT images obtained at baseline and year 1 to regress UPDRS-III scores at year 4. In contrast, we formulated prognostic prediction primarily as a classification task and introduced a multi-task fusion strategy in which the regression branch served as a complementary task to improve classification performance. We also evaluated a regression-only strategy using SWI images, and the results are shown in Table 2. As demonstrated in Table 2, the proposed multi-task fusion strategy outperformed the regression-only model. ~~In contrast to previous studies that need to design hand-crafted features and then used the traditional machine learning model[18,24,26,29], such as random forest, to predict deep brain stimulation outcome, our approach directly employs Susceptibility Weighted Imaging (SWI) images as the input for our model in an end-to-end manner. This eliminates the need for manually designing feature extraction operators. Hand-crafted features are typically carefully designed based~~

For Review Only

on visual neural theories to match the data's characteristics. However, this process introduces limitations associated with human intervention. In this work, the model itself extracts features from the substantia nigra and adjacent structures, enabling better extraction of hidden features. The work that most similar with us is Matthew's study [38], which developed a convolutional neural network using the SPECT images at year 0 and year 1 to regress the UPDRS III scores at year 4. Different with it[38] , we formulate the prognostic prediction as a classification task, and propose a multi-task fusion strategy to enhance the representation ability of the neural network, the regression network in our study only acts as a complementary-task to improve the performance of the classification task. Additionally, we also used the SWI images as input and tried to predict the UPDRS III scores with a regression network, which is similar with the network used in[38], the regression results is shown in Table 2. From Table 2, it is evident that our proposed multi-task fusion strategy outperforms the regression network.

We developed a deep learning-based multi-task fusion framework for SWI-based prognostic prediction of STN-DBS motor outcome.We employed a deep learning-based approach, defining the prognosis prediction task as a classification problem, and proposed a multi-task fusion network based on SWI images. By incorporating complementary-task learning, the model achieved an accuracy of 88.3% and an AUC of 0.873. These findings suggest that subtle differences in SNAS-centered SWI images may serve as promising imaging biomarkers related to DBS prognosis in PD and that the proposed model may provide neurosurgeons with a practical decision-support tool for identifying patients who are more likely to benefit fromBy employing the concept of complementary task learning, we conducted prognosis prediction, achieving an accuracy of 88.3% (AUC = 0.873). The results indicate the presence of subtle differences in substantia nigra SWI images, which may serve as promising biomarkers associated with the DBS prognosis in PD patients. This also suggests that our model could provide neurosurgeons with a novel and practical decision-support tool, aiding in the selection of Parkinson's disease patients suitable for surgery. Although feature map visualization suggested that the network mainly focused on the SNAS region, the proposed deep learning framework remains less transparent than conventional statistical or radiomics-based models. Therefore, the current visualization results should be regarded as a preliminary interpretability analysis rather than a complete explanation of the model decision-making process. Future studies should incorporate more advanced explainable artificial intelligence methods to improve model transparency and clinical interpretability.

We also performed exploratory analyses of DBS electrode localization and stimulation-related variables to address the potential influence of surgical and programming factors on postoperative outcome. Active contact coordinates did not significantly differ between optimal and suboptimal responders, suggesting that gross between-group differences in active contact location were unlikely to fully explain the observed model performance. However, suboptimal responders showed numerically larger VTA volume and VTA-STN overlap, particularly within the motor STN subregion. These findings should be interpreted cautiously because they were exploratory and did not remain significant after correction for multiple comparisons. One possible explanation is that patients with poorer motor response required broader or stronger stimulation during postoperative programming. We did not systematically model stimulation-induced collateral effects such as dysarthria, mood changes, or cognitive/behavioral adverse effects, which may also depend on contact location and current spread. Future studies should integrate preoperative imaging features, electrode localization, stimulation parameters, VTA-based metrics, and adverse-effect profiles into

For Review Only

a multimodal prognostic framework.

This study has several limitations. Firstly, the sample size was relatively small, which may limit the generalizability of the proposed model and increase the risk of overfitting. Although five-fold cross-validation was used to mitigate this issue, the findings should still be interpreted cautiously. More cases and a longer study period are required to further verify the robustness and effectiveness of the modelThis study has several limitations. Firstly, more cases need to be collected and a longer time span is required to further verify the effectiveness of the model. To mitigate potential overfitting issues arising from sample sizes, we employed a five-fold cross-validation. Secondly, no independent external validation cohort was available in the present study. The expanded 60-patient analysis was based on the same single-center retrospective cohort and should therefore be interpreted as an internal scalability analysis rather than external validation. Future multicenter prospective studies are needed to assess the robustness, reproducibility, and transportability of the proposed framework. ThirdlySecondly, the postoperative follow-up period in this study was limited to 1 year, and a more extendelongerd follow-up period would be essential to assess the long-term prognosis of patients. Additionally, although non-motor symptoms, motor complications, and quality-of-life measures were explored in supplementary analyses, these outcomes were not incorporated into the present prediction model and were limited by retrospective data availability. The current framework was also primarily developed using imaging features from preoperative SWI and did not fully integrate other potentially relevant clinical, neurophysiological, surgical, or postoperative programming variables, such as active-contact localization, VTA distribution, electric-field metrics, and longitudinal stimulation settings. Although these stimulation-related variables were explored in supplementary analyses, they were not incorporated into the present prediction model. Future studies may explore multimodal fusion strategies combining imaging biomarkers with clinical and surgical information to further improve predictive performance. Additionally, non-motor symptoms and motor complications significantly impact the prognosis and patient experience after DBS, but these factors were not considered in this study. Therefore, future research should conduct long-term follow-up studies with larger sample sizes to further elucidate the prognostic predictive power of preoperative SWI of the SNAS. Importantly, the proposed model is intended to assist rather than replace clinical decision-making. Prognostic predictions should be interpreted in the context of comprehensive clinical evaluation, surgical planning, patient preference, and multidisciplinary judgment.

In conclusion, our study demonstrated that preoperative SWI images covering the SNAS can be used for prognostic prediction of STN-DBS motor outcome in PD. To improve predictive performance, we proposed a deep learning-based multi-task fusion network that formulated prognostic prediction as a classification task and incorporated complementary regression learning based on continuous motor improvement rates. The proposed framework achieved a prognostic prediction accuracy of 88.3%, suggesting that it may provide a novel and practical decision-support tool for neurosurgeons and aid in identifying suitable PD patients for surgery. Overall, the framework provides a promising imaging-based strategy for preoperative prognostic assessment of STN-DBS in PD, although further external validation and methodological refinement are still required before routine clinical application. In the conclusion, our study demonstrated that the SWI images covering the SNAS can be used for prognostic prediction of STN-DBS for PD. To improve the prediction performance, we proposed a deep learning based multi-task fusion network. By formulating the prognostic prediction as a classification task, and fusing the complementary-task

regression learning results, our prognostic prediction accuracy achieves 88.3%, which suggests that our approach could offer a novel and practical decision-support tool for neurosurgeons and aid in screening suitable Parkinson's patients for surgery.

Data availability

The clinical and imaging data supporting the findings of this study are available from the corresponding author upon reasonable request, subject to institutional and ethical restrictions.

Code availability

The code used for model training, performance evaluation, statistical analysis, and DBS-related metric extraction is available at https://github.com/ceasorcai/PD. Clinical and imaging data are not publicly available because of institutional and ethical restrictions.

Declaration of Competing Interest

The authors declare that they have no known competing financial interests or personal relationships that could have appeared to influence the work reported in this paper.

FundingAcknowledgments

This work was supported in part by the National Natural Science Foundation of China under Grant 61973294, in part by the Anhui Provincial Key R&D Program under Grant (2023s07020017, 2022i01020020), in part by the University Synergy Innovation Program of Anhui Province, China, under Grant GXXT-2021-030, and in part by the Anhui Provincial Key Laboratory of Bionic Sensing and Advanced Robot Technology.

Competing interests

The authors declare no competing interests.

Author contributions

Chi Xiong: Data collection, data analysis, manuscript writing, study conception, and scientific integrity.

Kun Wang: Data analysis and manuscript writing.

Erkang Cheng: Critical revision of the manuscript.

Zhiyong Sun: Critical revision of the manuscript.

Bin Cai: Data analysis, manuscript writing, data collection, and critical revision of the manuscript.

Chaoshi Niu: Critical revision of the manuscript, study conception, and scientific integrity.

Bo Song: Critical revision of the manuscript.Chi Xiong: Data collection, data analysis, writing the manuscript, conceived the study and responsible for scientific integrity. Kung Wang: data analysis, writing the manuscript. Erkang Cheng: critical revision of the manuscript. Zhiyong Sun: critical revision of the manuscript. Bin Cai: data analysis, writing the manuscript, data collection, critical revision of the manuscript. Chaoshi Niu: critical revision of the manuscript, conceived the study and responsible for scientific integrity. Bo Song: critical revision of the manuscript.

References

1. Lachenmayer ML, Murset M, Antih N, et al. Subthalamic and pallidal deep brain stimulation for Parkinson's disease-meta-analysis of outcomes. NPJ Parkinsons Dis. 2021;7(1):77.

2. Liang K, Li RP, Gao Y, et al. Emotional symptoms and cognitive function outcomes of

For Review Only

subthalamic stimulation in Parkinson's disease depend on location of active contacts and the volume of tissue activated. CNS Neurosci Ther. 2023;29(8):2355-2365.
3. Geraedts VJ, Koch M, Contarino MF, et al. Machine learning for automated EEG-based biomarkers of cognitive impairment during deep brain stimulation screening in patients with Parkinson's disease. Clin Neurophysiol. 2021;132(5):1041-1048.
4. Saranza G, Lang AE. Levodopa challenge test: indications, protocol, and guide. J Neurol. 2021;268(9):3135-3143.
5. Lin W, Shi D, Wang D, et al. Can levodopa challenge testing predict the effect of deep brain stimulation? One-year outcomes in a Chinese cohort. Front Aging Neurosci. 2021;13:764308.
6. Geraedts VJ, van Vugt JPP, Marinus J, Kuiper R, et al. Predicting motor outcome and quality of life after subthalamic deep brain stimulation for Parkinson's disease: the role of standard screening measures and wearable-data. J Parkinsons Dis. 2023;13(4):575-588.
7. Mueller K, Urgošík D, Ballarini T, et al. Differential effects of deep brain stimulation and levodopa on brain activity in Parkinson's disease. Brain Commun. 2020;2(1):fcaa005.
8. Zhao CH, Wu HT, Che HB, et al. Prediction of fatal adverse prognosis in patients with fever-related diseases based on machine learning: A retrospective study. Chin Med J (Engl). 2020;133(5):583-589.
9. Qiu QT, Zhang J, Duan JH, et al. Development and validation of radiomics model built by incorporating machine learning for identifying liver fibrosis and early-stage cirrhosis. Chin Med J (Engl). 2020;133(22):2653-2659.
10. Zhu X, Shi W, Ling Y, et al. Contactless evaluation of rigidity in Parkinson's disease by machine vision and machine learning. Chin Med J (Engl). 2023;136(18):2254-2256.
11. Postuma RB, Berg D, Stern M, et al. MDS clinical diagnostic criteria for Parkinson's disease. Mov Disord. 2015;30(12):1591-1601.
12. Hoops S, Nazem S, Siderowf AD, et al. Validity of the MoCA and MMSE in the detection of MCI and dementia in Parkinson disease. Neurology. 2009;73(21):1738-1745.
13. Castrioto A, Lozano AM, Poon YY, et al. Ten-year outcome of subthalamic stimulation in Parkinson disease: a blinded evaluation. Arch Neurol. 2011;68(12):1550-1556.
14. Defer GL, Widner H, Marie RM, Remy P, Levivier M. Core assessment program for surgical interventional therapies in Parkinson's disease (CAPSIT-PD). Mov Disord. 1999;14(4):572-584.
15. Bronstein JM, Tagliati M, Alterman RL, et al. Deep brain stimulation for Parkinson disease: an expert consensus and review of key issues. Arch Neurol. 2011;68(2):165.
16. de Oliveira LM, Barbosa ER, Aquino CC, et al. Deep brain stimulation in patients with mutations in Parkinson's disease-related genes: A systematic review. Mov Disord Clin Pract. 2019;6(5):359-368.
17. Tang V, Zhu XL, Lau C, et al. Pre-operative cognitive burden as predictor of motor outcome following bilateral subthalamic nucleus deep brain stimulation in Parkinson's disease. Neurol Sci. 2022;43(12):6803-6811.
18. Habets J, Janssen M, Duits AA, et al. Machine learning prediction of motor response after deep brain stimulation in Parkinson's disease-proof of principle in a retrospective cohort. PeerJ. 2020;8:e10317.
19. Ozturk M, Telkes I, Jimenez-Shahed J, et al. Randomized, double-blind assessment of LFP versus SUA guidance in STN-DBS lead implantation: a pilot study. Front Neurosci. 2020;14:611.

For Review Only

20. Lagerweij S, van Zijl JC, van Laar T, et al. Intermuscular coherence as a biomarker of subthalamic nucleus deep brain stimulation efficacy in Parkinson's disease. Clin Neurophysiol. 2022;142:36-43.
21. Geraedts VJ, Koch M, Kuiper R, et al. Preoperative electroencephalography-based machine learning predicts cognitive deterioration after subthalamic deep brain stimulation. Mov Disord. 2021;36(10):2324-2334.
22. Wu C, Matias C, Foltynie T, et al. Dynamic network connectivity reveals markers of response to deep brain stimulation in Parkinson's disease. Front Hum Neurosci. 2021;15:729677.
23. Frizon LA, Gopalakrishnan R, Hogue O, et al. Cortical thickness in visuo-motor areas is related to motor outcomes after STN DBS for Parkinson's disease. Parkinsonism Relat Disord. 2020;71:17-22.
24. Liu Y, Xiao B, Zhang C, et al. Predicting motor outcome of subthalamic nucleus deep brain stimulation for Parkinson's disease using quantitative susceptibility mapping and radiomics: A pilot study. Front Neurosci. 2021;15:731109.
25. Xiao B, He N, Wang Q, et al. Quantitative susceptibility mapping based hybrid feature extraction for diagnosis of Parkinson's disease. Neuroimage Clin. 2019;24:102070.
26. Hirschmann J, Steina A, Vesper J, Florin E, Schnitzler A. Neuronal oscillations predict deep brain stimulation outcome in Parkinson's disease. Brain Stimul. 2022;15(3):792-802.
27. Saudargiene A, Radziunas A, Dainauskas JJ, et al. Radiomic features of amygdala nuclei and hippocampus subfields help to predict subthalamic deep brain stimulation motor outcomes for Parkinson's disease patients. Front Neurosci. 2022;16:1028996.
28. Tsai CC, Lin YC, Ng SH, et al. A method for the prediction of clinical outcome using diffusion magnetic resonance imaging: application on Parkinson's disease. J Clin Med. 2020;9(3):647.
29. Wang J, Shang R, He L, et al. Prediction of deep brain stimulation outcome in Parkinson's disease with connectome based on hemispheric asymmetry. Front Neurosci. 2021;15:620750.
30. Hardman CD, McRitchie DA, Halliday GM, Cartwright HR, Morris JG. Substantia nigra pars reticulata neurons in Parkinson's disease. Neurodegeneration. 1996;5(1):49-55.
31. Ariz M, Martinez M, Alvarez I, et al. Automatic segmentation and quantification of Nigrosome-1 neuromelanin and iron in MRI: A candidate biomarker for Parkinson's disease. J Magn Reson Imaging. 2024;60(2):534-547.
32. Yan S, Lu J, Li Y, et al. Spatiotemporal patterns of brain iron-oxygen metabolism in patients with Parkinson's disease. Eur Radiol. 2024;34(5):3074-3083.
33. Bu S, Pang H, Li X, et al. Spatiotemporal nigrostriatal iron accumulation in motor subtypes of Parkinson's disease: from early to late stage. Brain Commun. 2025;7(6):fcaf473.
34. Mazumder S, Bahar AY, Shepherd CE, Prasad AA. Post-mortem brain histological examination in the substantia nigra and subthalamic nucleus in Parkinson's disease following deep brain stimulation. Front Neurosci. 2022;16:948523.
35. Rodriguez RL, Fernandez HH, Haq I, Okun MS. Pearls in patient selection for deep brain stimulation. Neurologist. 2007;13(5):253-260.
36. Hayashi Y, Mishima T, Fujioka S, et al. Unilateral GPi-DBS improves ipsilateral and axial motor symptoms in Parkinson's disease as evidenced by a brain perfusion single photon emission computed tomography study. Front Hum Neurosci. 2022;16:888701.
37. Schuepbach W, Tonder L, Schnitzler A, et al. Quality of life predicts outcome of deep brain stimulation in early Parkinson disease. Neurology. 2019;92(10):e1109-e1120.

For Review Only

38. Adams MP, Rahmim A, Tang J. Improved motor outcome prediction in Parkinson's disease applying deep learning to DaTscan SPECT images. Comput Biol Med. 2021;132:104312.

Figure legends

Figure 1. Multi-task fusion framework. Overview of the proposed framework for prognostic prediction of subthalamic nucleus deep brain stimulation motor outcome in Parkinson's disease. A preoperative susceptibility-weighted imaging sub-volume covering the substantia nigra and adjacent structures is first processed by a three-dimensional feature-learning backbone. The extracted features are then fed into a regression branch and a classification branch. The outputs of the two branches are fused by a logistic regression classifier to generate the final prognostic prediction. The arrow indicates the substantia nigra.

Figure 2. Classification pathway. Architecture of the classification pathway. The input substantia nigra and adjacent structures-centered three-dimensional susceptibility-weighted imaging sub-volume is processed by three consecutive three-dimensional convolutional blocks, each consisting of Conv3d, InstanceNorm, LeakyReLU, and MaxPool3d. The resulting feature representation is then passed through the classification head composed of dropout, flattening, and three linear layers. The first two linear layers are followed by rectified linear unit activation, and the final linear layer is followed by a softmax operation to generate class probabilities. The arrow indicates the substantia nigra.

Figure 3. Multi-task fusion architecture. Architecture of the proposed multi-task fusion framework. The input substantia nigra and adjacent structures-centered three-dimensional susceptibility-weighted imaging sub-volume is first processed by a shared three-dimensional convolutional backbone. The learned features are then fed into two parallel branches: a classification branch for predicting optimal versus suboptimal motor outcome and a regression branch for predicting the continuous Unified Parkinson's Disease Rating Scale Part III improvement rate. The outputs of the two branches are finally fused by a logistic regression classifier to generate the final prognostic prediction. The arrow indicates the substantia nigra.

Figure 4. Anatomical specificity analysis. Illustration of the three-dimensional sub-regions extracted from preoperative susceptibility-weighted imaging for anatomical specificity analysis. The outlined boxes indicate substantia nigra and adjacent structures-centered sub-regions used for model training at different spatial scales and a control sub-region extracted from a non-substantia nigra and adjacent structures location. The substantia nigra and adjacent structures-centered region covered the substantia nigra and adjacent deep brain structures, including part of the subthalamic nucleus region. These experiments were used to examine whether prognostic information was concentrated in the substantia nigra and adjacent structures.

Figure 5. Receiver operating characteristic curves. Receiver operating characteristic curves of the five-fold cross-validation in the expanded 60-patient cohort. Thin lines represent the receiver operating characteristic curves of individual folds, and the thick blue line represents the mean receiver operating characteristic curve.

Figure 6. Feature map visualization. Visualization of feature maps before each pooling

For Review Only

operation in the backbone. The heatmaps show that feature responses progressively concentrate around the substantia nigra and adjacent structures, suggesting that the model primarily extracted prognostically relevant information from the substantia nigra and adjacent structures-centered region.References:

1. Lachenmayer ML, Murset M, Antih N et al. Subthalamic and pallidal deep brain stimulation for Parkinson's disease-meta-analysis of outcomes. NPJ Parkinsons Dis. 2021;7(1):77

2. Liang K, Li RP, Gao Y et al. Emotional symptoms and cognitive function outcomes of subthalamic stimulation in Parkinson's disease depend on location of active contacts and the volume of tissue activated. CNS Neurosci Ther. 2023;29(8):2355-2365

3. Geraedts VJ, Koch M, Contarino MF et al. Machine learning for automated EEG-based biomarkers of cognitive impairment during Deep Brain Stimulation screening in patients with Parkinson's Disease. Clin Neurophysiol. 2021;132(5):1041-1048

4. Saranza G, Lang AE. Levodopa challenge test: indications, protocol, and guide. J Neurol. 2021;268(9):3135-3143

5. Lin W, Shi D, Wang D et al. Can Levodopa Challenge Testing Predict the Effect of Deep Brain Stimulation? One-Year Outcomes in a Chinese Cohort. Front Aging Neurosci. 2021;13:764308

6. Tsafack EG, Mbiantcha M, Ateufack G et al. Antihypernociceptive and Neuroprotective Effects of the Aqueous and Methanol Stem-Bark Extracts of Nauclea pobeguinii (Rubiaceae) on STZ-Induced Diabetic Neuropathic Pain. Evid Based Complement Alternat Med. 2021;2021:6637584

7. Mueller K, Urgošík D, Ballarini T et al. Differential effects of deep brain stimulation and levodopa on brain activity in Parkinson's disease. Brain Commun. 2020;2(1):fcaa005

8. Zhao CH, Wu HT, Che HB et al. Prediction of fatal adverse prognosis in patients with fever-related diseases based on machine learning: A retrospective study. Chin Med J (Engl). 2020;133(5):583-589

9. Qiu QT, Zhang J, Duan JH et al. Development and validation of radiomics model built by incorporating machine learning for identifying liver fibrosis and early-stage cirrhosis. Chin Med J (Engl). 2020;133(22):2653-2659

10. Zhu X, Shi W, Ling Y et al. Contactless evaluation of rigidity in Parkinson's disease by machine vision and machine learning. Chin Med J (Engl). 2023;136(18):2254-2256

11. Postuma RB, Berg D, Stern M et al. MDS clinical diagnostic criteria for Parkinson's disease. Mov Disord. 2015;30(12):1591-601

12. Hoops S, Nazem S, Siderowf AD et al. Validity of the MoCA and MMSE in the detection of MCI and dementia in Parkinson disease. Neurology. 2009;73(21):1738-45

13. Castrioto A, Lozano AM, Poon YY et al. Ten-year outcome of subthalamic stimulation in Parkinson disease: a blinded evaluation. Arch Neurol. 2011;68(12):1550-6

14. Defer GL, Widner H, Marie RM, Remy P, Levivier M. Core assessment program for surgical interventional therapies in Parkinson's disease (CAPSIT-PD). Mov Disord. 1999;14(4):572-84

15. Bronstein JM, Tagliati M, Alterman RL et al. Deep brain stimulation for Parkinson disease: an expert consensus and review of key issues. Arch Neurol. 2011;68(2):165

16. de Oliveira LM, Barbosa ER, Aquino CC et al. Deep Brain Stimulation in Patients With Mutations in Parkinson's Disease-Related Genes: A Systematic Review. Mov Disord Clin

For Review Only

Pract. 2019;6(5):359-368
17. Tang V, Zhu XL, Lau C et al. Pre-operative cognitive burden as predictor of motor outcome following bilateral subthalamic nucleus deep brain stimulation in Parkinson's disease. Neurol Sci. 2022;43(12):6803-6811
18. Habets J, Janssen M, Duits AA et al. Machine learning prediction of motor response after deep brain stimulation in Parkinson's disease-proof of principle in a retrospective cohort. PeerJ. 2020;8:e10317
19. Ozturk M, Telkes I, Jimenez-Shahed J et al. Randomized, Double-Blind Assessment of LFP Versus SUA Guidance in STN-DBS Lead Implantation: A Pilot Study. Front Neurosci. 2020;14:611
20. Lagerweij S, van Zijl JC, van Laar T et al. Intermuscular coherence as a biomarker of subthalamic nucleus deep brain stimulation efficacy in Parkinson's disease. Clin Neurophysiol. 2022;142:36-43
21. Geraedts VJ, Koch M, Kuiper R et al. Preoperative Electroencephalography-Based Machine Learning Predicts Cognitive Deterioration After Subthalamic Deep Brain Stimulation. Mov Disord. 2021;36(10):2324-2334
22. Wu C, Matias C, Foltynie T et al. Dynamic Network Connectivity Reveals Markers of Response to Deep Brain Stimulation in Parkinson's Disease. Front Hum Neurosci. 2021;15:729677
23. Frizon LA, Gopalakrishnan R, Hogue O et al. Cortical thickness in visuo-motor areas is related to motor outcomes after STN DBS for Parkinson's disease. Parkinsonism Relat Disord. 2020;71:17-22
24. Liu Y, Xiao B, Zhang C et al. Predicting Motor Outcome of Subthalamic Nucleus Deep Brain Stimulation for Parkinson's Disease Using Quantitative Susceptibility Mapping and Radiomics: A Pilot Study. Front Neurosci. 2021;15:731109
25. Xiao B, He N, Wang Q et al. Quantitative susceptibility mapping based hybrid feature extraction for diagnosis of Parkinson's disease. Neuroimage Clin. 2019;24:102070
26. Hirschmann J, Steina A, Vesper J, Florin E, Schnitzler A. Neuronal oscillations predict deep brain stimulation outcome in Parkinson's disease. Brain Stimul. 2022;15(3):792-802
27. Saudargiene A, Radziunas A, Dainauskas JJ et al. Radiomic features of amygdala nuclei and hippocampus subfields help to predict subthalamic deep brain stimulation motor outcomes for Parkinson's disease patients. Front Neurosci. 2022;16:1028996
28. Tsai CC, Lin YC, Ng SH et al. A Method for the Prediction of Clinical Outcome Using Diffusion Magnetic Resonance Imaging: Application on Parkinson's Disease. J Clin Med. 2020;9(3)
29. Wang J, Shang R, He L et al. Prediction of Deep Brain Stimulation Outcome in Parkinson's Disease With Connectome Based on Hemispheric Asymmetry. Front Neurosci. 2021;15:620750
30. Hardman CD, McRitchie DA, Halliday GM, Cartwright HR, Morris JG. Substantia nigra pars reticulata neurons in Parkinson's disease. Neurodegeneration. 1996;5(1):49-55
31. Ariz M, Martinez M, Alvarez I et al. Automatic Segmentation and Quantification of Nigrosome-1 Neuromelanin and Iron in MRI: A Candidate Biomarker for Parkinson's Disease. J Magn Reson Imaging. 2024;60(2):534-547
32. Yan S, Lu J, Li Y et al. Spatiotemporal patterns of brain iron-oxygen metabolism in patients with Parkinson's disease. Eur Radiol. 2024;34(5):3074-3083
33. Bu S, Pang H, Li X et al. Spatiotemporal nigrostriatal iron accumulation in motor subtypes of

For Review Only

Parkinson's disease: from early to late stage. Brain Commun. 2025;7(6):fcaf473
34. Mazumder S, Bahar AY, Shepherd CE, Prasad AA. Post-mortem brain histological examination in the substantia nigra and subthalamic nucleus in Parkinson's disease following deep brain stimulation. Front Neurosci. 2022;16:948523
35. Rodriguez RL, Fernandez HH, Haq I, Okun MS. Pearls in patient selection for deep brain stimulation. Neurologist. 2007;13(5):253-60
36. Hayashi Y, Mishima T, Fujioka S et al. Unilateral GPi-DBS Improves Ipsilateral and Axial Motor Symptoms in Parkinson's Disease as Evidenced by a Brain Perfusion Single Photon Emission Computed Tomography Study. Front Hum Neurosci. 2022;16:888701
37. Schuepbach W, Tonder L, Schnitzler A et al. Quality of life predicts outcome of deep brain stimulation in early Parkinson disease. Neurology. 2019;92(10):e1109-e1120
38. Adams MP, Rahmim A, Tang J. Improved motor outcome prediction in Parkinson's disease applying deep learning to DaTscan SPECT images. Comput Biol Med. 2021;132:104312

For Review Only

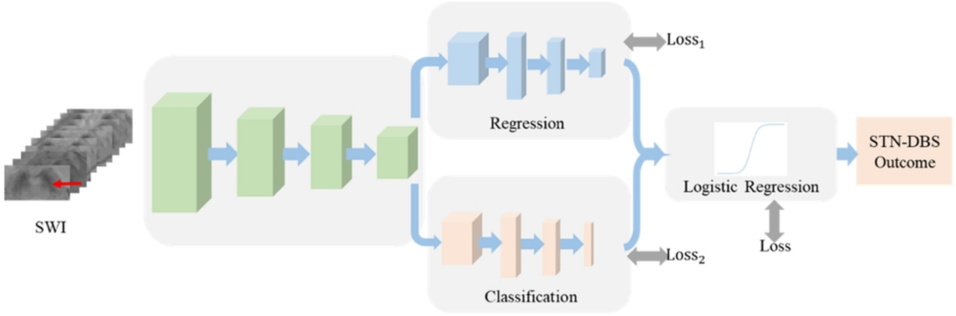

Figure 1

192x69mm (300 x 300 DPI)

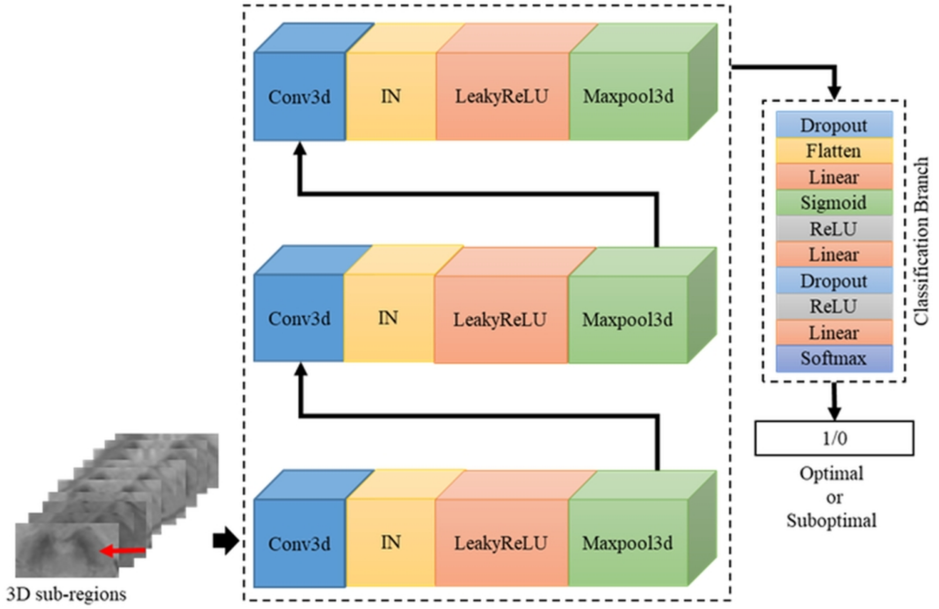

Figure 2

130x85mm (300 x 300 DPI)

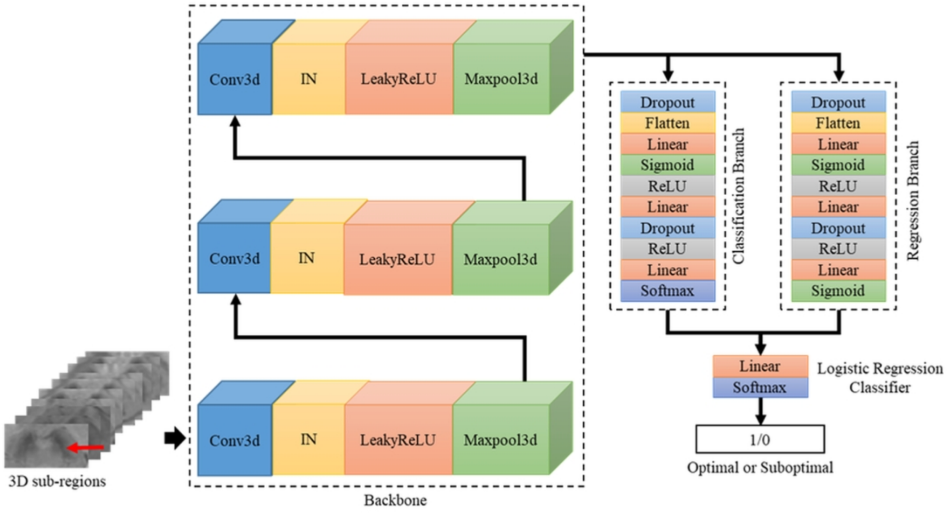

Figure 3

162x91mm (300 x 300 DPI)

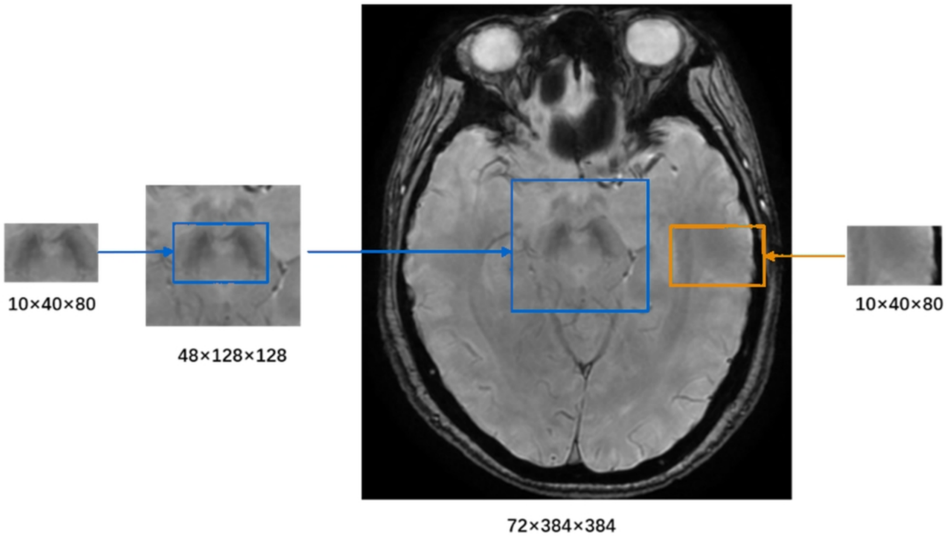

Figure 4

164x97mm (300 x 300 DPI)

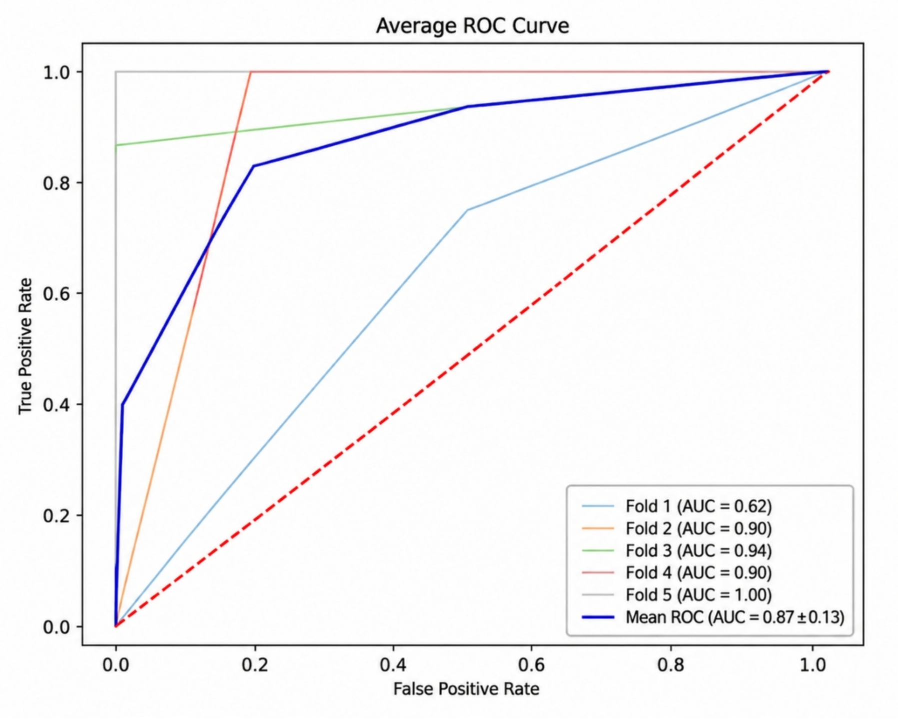

Figure 5

169x138mm (300 x 300 DPI)

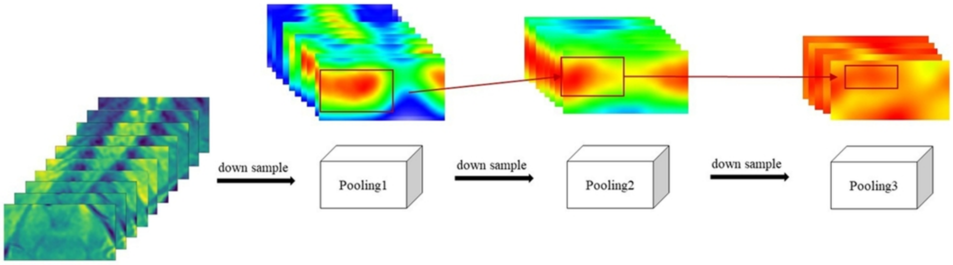

Figure 6

200x61mm (300 x 300 DPI)

Supplementary Material

Development and Application of a Multi-Task Fusion Model Using SWI-based Substantia Nigra and Adjacent Structures for Prognostic Prediction of STN-DBS in Parkinson's Disease

Abbreviations: AUC, area under the receiver operating characteristic curve; BN, batch normalization; CT, computed tomography; DBS, deep brain stimulation; FDR, false discovery rate; HAMA, Hamilton Anxiety Rating Scale; HAMD, Hamilton Depression Rating Scale; LCT, levodopa challenge test; LEDD, levodopa equivalent daily dose; MNI, Montreal Neurological Institute; MoCA, Montreal Cognitive Assessment; MRI, magnetic resonance imaging; NMSS, Non-Motor Symptoms Scale; PDQ-39, 39-item Parkinson's Disease Questionnaire; PDSS, Parkinson's Disease Sleep Scale; SNAS, substantia nigra and adjacent structures; STN, subthalamic nucleus; SWI, susceptibility-weighted imaging; TEED, total electrical energy delivered; UPDRS-III, Unified Parkinson's Disease Rating Scale Part III; VTA, volume of tissue activated.

Supplementary Figures

Supplementary Figure 1. Architecture of the 3D ResNet50 baseline model.

For Review Only

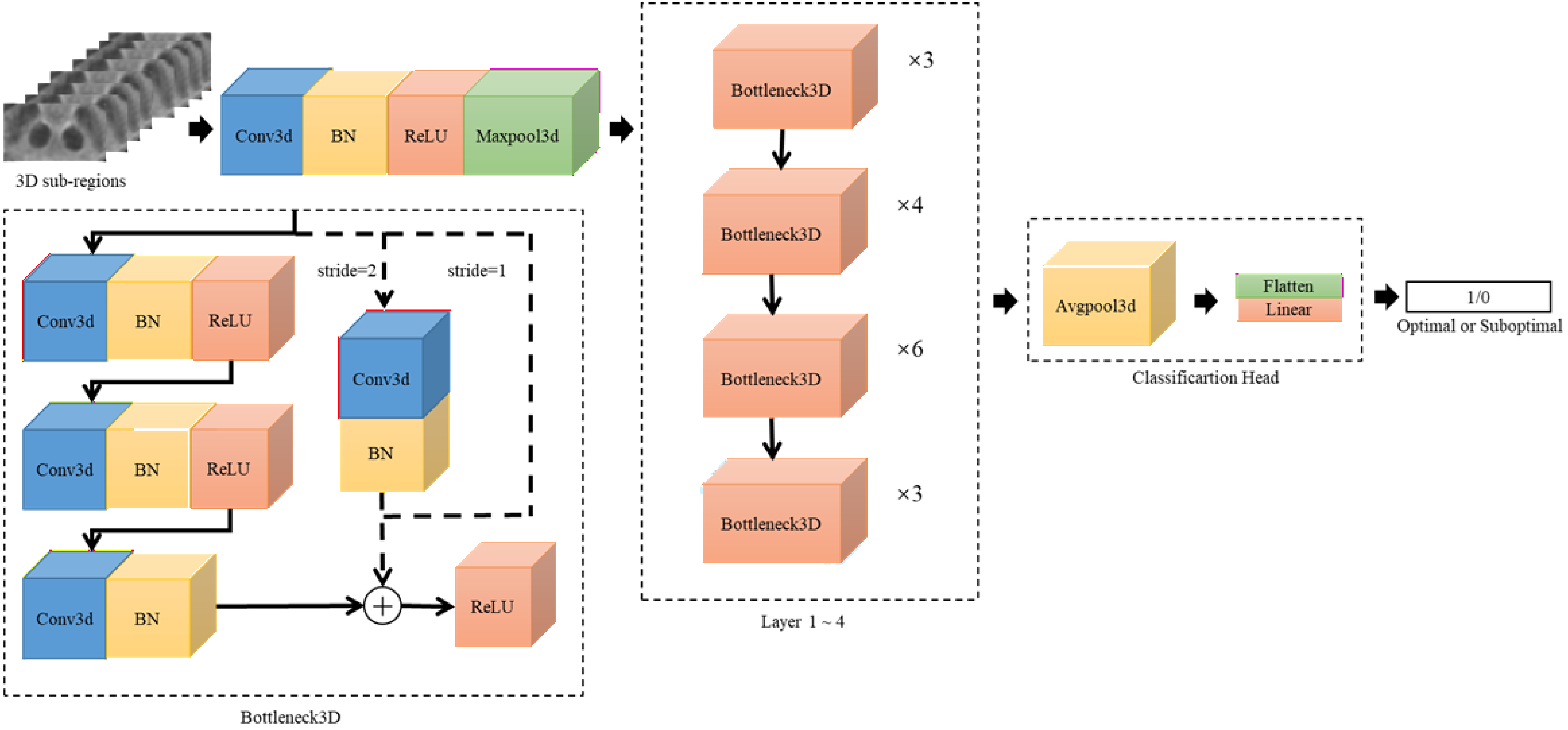

Legend: The 3D SWI sub-volume was processed by an initial convolutional block, four Bottleneck3D residual stages, global average pooling, and a linear classification head to predict optimal versus suboptimal motor response. BN indicates batch normalization.

Supplementary Figure 2. Representative illustration of SNAS-centered sub-volume extraction from preoperative SWI.

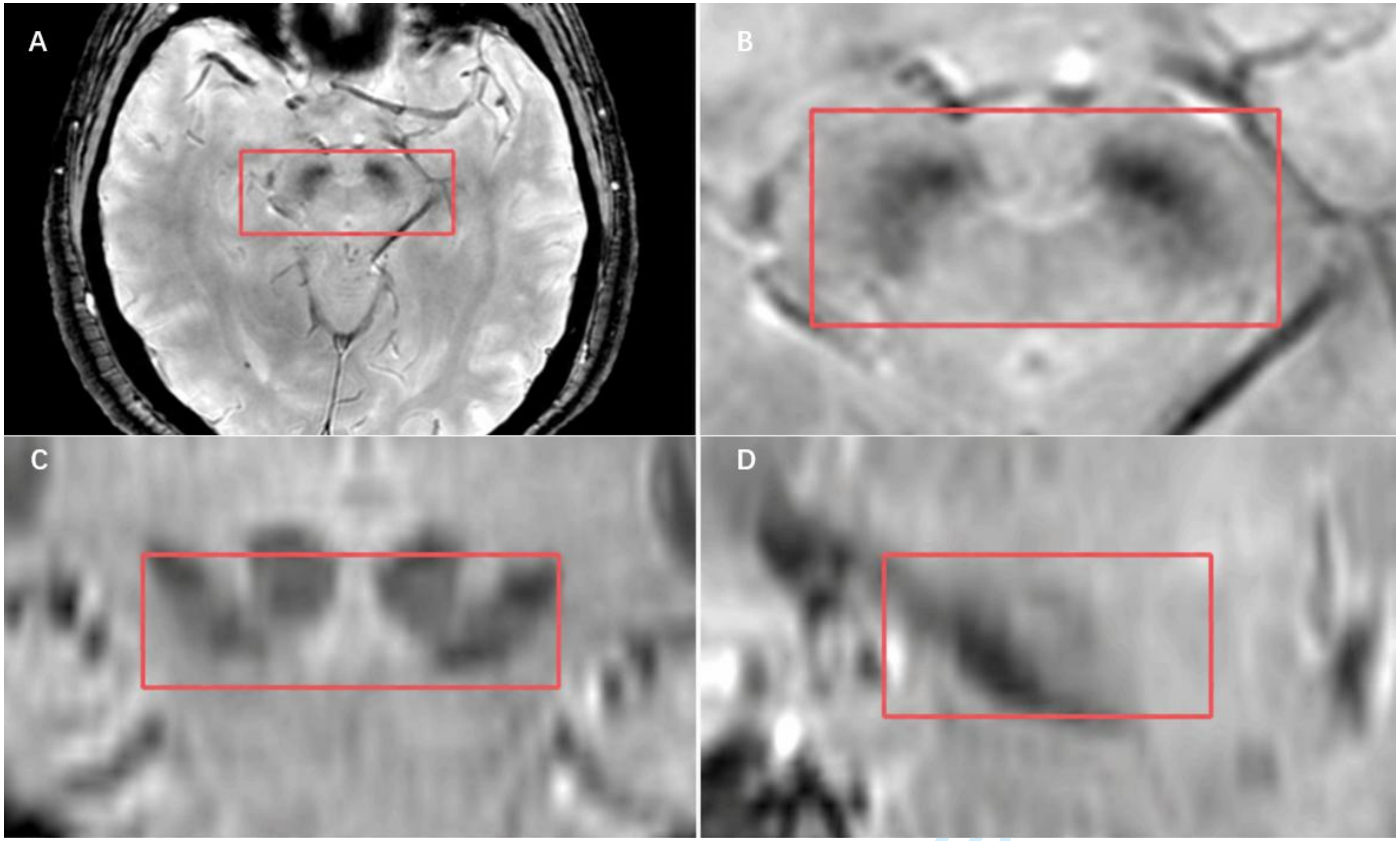

Legend: (A) Whole axial SWI image showing the anatomical location of the selected SNAS-centered region. (B) Magnified axial view showing that the selected region covered the bilateral substantia nigra and adjacent structures. (C, D) Coronal and sagittal views illustrating the three-dimensional spatial extent of the extracted sub-volume.

The red box denotes the fixed-size SNAS-centered sub-volume manually localized on 3D SWI images, with dimensions of 10 × 40 × 80 voxels. The extracted region covered the substantia nigra and adjacent deep brain structures, including part of the STN region.

Supplementary Figure 3. Group-level Lead-DBS reconstruction of bilateral STN-DBS electrodes and active contacts.

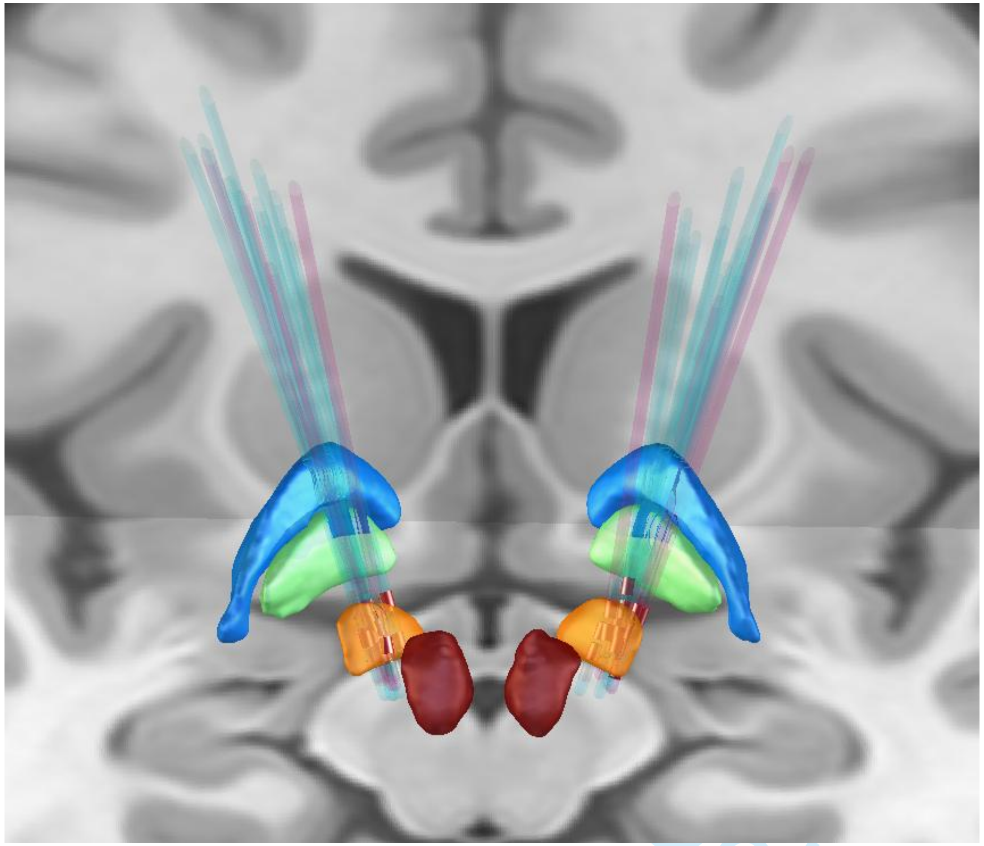

Legend: Postoperative CT images were co-registered with preoperative MRI, and electrode trajectories and active contacts were reconstructed using Lead-DBS. Electrode trajectories from optimal responders are shown in cyan, whereas those from suboptimal responders are shown in magenta. The clinically active contacts are highlighted on each electrode. Colored surfaces represent atlas-defined STN functional subregions and adjacent deep brain structures to illustrate the anatomical relationship between electrode locations and the STN region. Quantitative active contact coordinates, stimulation parameters, VTA metrics, and VTA-STN overlap analyses are provided in Supplementary Tables 5–7.

Supplementary Tables

Supplementary Table 1. Detailed demographic and baseline clinical characteristics.

Variable	Optimal responders, mean ± SD	Suboptimal responders, mean ± SD	Optimal responders, median [IQR]	Suboptimal responders, median [IQR]	Test	Statistic	P value	Rank-biserial r
Age, years	56.19 ± 6.51	60.00 ± 6.66	56.00 [52.00, 61.00]	59.00 [55.00, 65.00]	Mann–Whitney U	307.00	0.072	-0.278
Sex, male/female	22/15	11/12			Fisher exact		0.431	
Disease duration, years	8.38 ± 2.91	8.91 ± 3.92	9.00 [6.00, 10.00]	8.00 [6.00, 10.00]	Mann–Whitney U	436.00	0.878	0.025
Age at onset, years	48.62 ± 7.90	51.09 ± 7.12	48.00 [44.00, 53.00]	51.00 [46.00, 54.50]	Mann–Whitney U	334.00	0.166	-0.215
Hoehn-Yahr stage	3.31 ± 0.73	3.37 ± 0.68	3.00 [3.00, 4.00]	3.00 [3.00, 4.00]	Mann–Whitney U	389.00	0.545	-0.086
LEDD, mg	714.86 ± 383.90	646.20 ± 241.43	650.00 [537.50, 800.00]	675.00 [450.00, 837.50]	Mann–Whitney U	440.50	0.825	0.035
LCT improvement rate	0.57 ± 0.13	0.52 ± 0.14	0.58 [0.49, 0.67]	0.52 [0.42, 0.61]	Mann–Whitney U	521.50	0.146	0.226

UPDRS-III	54.38 ± 12.67	51.78 ± 10.86	52.00 [47.00, 64.00]	49.00 [45.50, 57.00]	Mann–Whitney U	479.00	0.420	0.126
UPDRS-IV	6.03 ± 1.77	6.30 ± 1.43	6.00 [5.00, 7.00]	6.00 [5.50, 7.00]	Mann–Whitney U	386.50	0.548	-0.092
NMSS	72.19 ± 35.91	76.30 ± 28.37	62.00 [48.00, 95.00]	70.00 [55.00, 93.50]	Mann–Whitney U	374.50	0.442	-0.120
PDQ-39	62.30 ± 19.06	67.09 ± 19.11	58.00 [49.00, 76.00]	63.00 [56.00, 80.00]	Mann–Whitney U	367.00	0.378	-0.137
MMSE	26.57 ± 2.72	24.78 ± 4.26	27.00 [25.00, 29.00]	26.00 [21.00, 28.00]	Mann–Whitney U	516.00	0.168	0.213
MoCA	20.92 ± 5.23	20.09 ± 6.22	22.00 [18.00, 25.00]	20.00 [16.00, 25.00]	Mann–Whitney U	461.00	0.594	0.083
HAMD	12.81 ± 5.83	12.70 ± 5.25	13.00 [9.00, 17.00]	13.00 [8.00, 16.00]	Mann–Whitney U	432.00	0.927	0.015
HAMA	14.24 ± 5.85	17.70 ± 6.57	15.00 [10.00, 18.00]	17.00 [12.00, 23.00]	Mann–Whitney U	305.50	0.069	-0.282
PDSS	99.46 ± 24.56	99.00 ± 19.54	100.00 [81.00, 120.00]	98.00 [90.00, 114.00]	Mann–Whitney U	444.50	0.778	0.045
Constipation score	10.22 ± 6.34	11.61 ± 5.15	9.00 [6.00, 14.00]	13.00 [8.50, 14.50]	Mann–Whitney U	347.00	0.235	-0.184

Clinical baseline variables including age, sex, disease duration, age at onset, LEDD, Hoehn-Yahr stage, preoperative UPDRS-III, and LCT improvement rate were derived from the original 60-patient clinical table after manual correction of a known age entry error for one patient. Group classification was based on the finalized outcome definition in the analysis-ready dataset.

Supplementary Table 2. Additional clinical outcomes beyond UPDRS-III.

Outcome	Direction of favorable change	Preoperative optimal	Preoperative suboptimal	Preoperative P	Postoperative optimal	Postoperative suboptimal	Postoperative P	Favorable change optimal	Favorable change suboptimal	Change P	Change FDR q	Favorable change, % optimal	Favorable change, % suboptimal	Percentage change P	Percentage change FDR q
LEDD, mg	Pre − post	714.86 ± 383.90	646.20 ± 241.43	0.825	483.50 ± 253.09	409.74 ± 214.18	0.283	231.36 ± 368.14	236.46 ± 277.59	0.704	0.873	27.69 ± 38.45	27.51 ± 50.48	0.654	0.921
UPDRS-IV	Pre − post	6.03 ± 1.77	6.30 ± 1.43	0.548	4.22 ± 2.48	5.00 ± 1.48	0.174	1.81 ± 3.41	1.30 ± 1.96	0.736	0.873	20.70 ± 53.87	17.24 ± 30.61	0.546	0.910
NMSS	Pre − post	72.19 ± 35.91	76.30 ± 28.37	0.442	51.14 ± 31.64	62.22 ± 33.46	0.204	21.05 ± 39.38	14.09 ± 33.57	0.687	0.873	-38.64 ± 368.49	13.18 ± 41.78	0.543	0.910
PDQ-39	Pre − post	62.30 ± 19.06	67.09 ± 19.11	0.378	37.95 ± 22.30	56.13 ± 20.30	0.005	24.35 ± 21.58	10.96 ± 19.05	0.011	0.106	39.19 ± 31.09	13.75 ± 28.05	0.003	0.031
MMSE	Post − pre	26.57 ± 2.72	24.78 ± 4.26	0.168	26.78 ± 3.37	24.78 ± 5.15	0.180	0.22 ± 2.37	0.00 ± 4.08	0.800	0.873	0.91 ± 8.98	0.94 ± 19.57	0.921	0.921
MoCA	Post − pre	20.92 ± 5.23	20.09 ± 6.22	0.594	23.16 ± 4.94	20.83 ± 5.57	0.085	2.24 ± 3.45	0.74 ± 4.01	0.136	0.680	13.88 ± 20.97	8.33 ± 29.97	0.138	0.689
HAMD	Pre − post	12.81 ± 5.83	12.70 ± 5.25	0.927	8.97 ± 6.03	11.61 ± 6.24	0.107	3.84 ± 8.20	1.09 ± 7.17	0.241	0.804	12.22 ± 68.36	-0.07 ± 59.47	0.338	0.910
HAMA	Pre − post	14.24 ± 5.85	17.70 ± 6.57	0.069	8.92 ± 6.05	12.26 ± 7.29	0.076	5.32 ± 8.71	5.43 ± 8.73	0.873	0.873	16.14 ± 77.99	22.19 ± 54.52	0.861	0.921
PDSS	Post − pre	99.46 ± 24.56	99.00 ± 19.54	0.778	114.84 ± 24.59	110.17 ± 24.50	0.204	15.38 ± 32.34	11.17 ± 20.75	0.461	0.873	23.36 ± 41.44	13.19 ± 28.16	0.494	0.910
Constipation score	Pre − post	10.22 ± 6.34	11.61 ± 5.15	0.235	8.83 ± 7.93	9.70 ± 6.14	0.334	1.39 ± 8.64	1.91 ± 5.26	0.703	0.873	-6.47 ± 88.05	12.22 ± 56.12	0.738	0.921

LEDD baseline values were derived from the original clinical table, while postoperative and additional scale variables were obtained from the finalized merged dataset. For scales in which lower scores indicate better status, favorable change was defined as preoperative score minus postoperative score. For scales in which higher scores indicate better status, favorable change was defined as postoperative score minus preoperative score. P values were calculated using Mann–Whitney U tests. FDR correction was performed using the Benjamini–Hochberg method.

Supplementary Table 3. Individual preoperative and postoperative UPDRS-III scores and continuous improvement rates.

ID	Preoperative UPDRS-III	1-year postoperative stimulation-on/medication-off UPDRS-III	UPDRS-III improvement rate	Response classification
p001	60	11	81.67%	optimal
p002	63	28	55.56%	optimal
p003	51	18	64.71%	optimal
p004	55	22	60.00%	optimal
p005	78	37	52.56%	optimal
p006	65	35	46.15%	suboptimal
p007	47	40	14.89%	suboptimal
p008	65	23	64.62%	optimal
p009	69	21	69.57%	optimal
p010	37	10	72.97%	optimal
p011	57	33	42.11%	suboptimal
p012	47	22	53.19%	optimal
p013	52	39	25.00%	suboptimal
p014	63	49	22.22%	suboptimal
p015	48	38	20.83%	suboptimal
p016	44	17	61.36%	optimal
p017	42	30	28.57%	suboptimal
p018	49	43	12.24%	suboptimal
p019	43	20	53.49%	optimal
p020	43	21	51.16%	optimal
p021	34	11	67.65%	optimal
p022	62	22	64.52%	optimal
p023	75	35	53.33%	optimal
p024	70	39	44.29%	suboptimal
p025	57	31	45.61%	suboptimal
p026	56	34	39.29%	suboptimal
p027	78	18	76.92%	optimal
p028	66	42	36.36%	suboptimal
p029	73	64	12.33%	suboptimal
p030	70	28	60.00%	optimal
p031	57	30	47.37%	suboptimal
p032	39	5	87.18%	optimal
p033	69	5	92.75%	optimal
p034	45	27	40.00%	suboptimal
p035	52	23	55.77%	optimal
p036	69	19	72.46%	optimal
p037	57	20	64.91%	optimal

For Review Only

p038	54	28	48.15%	suboptimal
p039	34	4	88.24%	optimal
p040	42	24	42.86%	suboptimal
p041	51	24	52.94%	optimal
p042	28	16	42.86%	suboptimal
p043	32	9	71.88%	optimal
p044	46	27	41.30%	suboptimal
p045	39	10	74.36%	optimal
p046	47	9	80.85%	optimal
p047	47	20	57.45%	optimal
p048	58	23	60.34%	optimal
p049	59	20	66.10%	optimal
p050	68	20	70.59%	optimal
p051	64	5	92.19%	optimal
p052	51	19	62.75%	optimal
p053	54	18	66.67%	optimal
p054	43	28	34.88%	suboptimal
p055	49	18	63.27%	optimal
p056	47	24	48.94%	suboptimal
p057	38	27	28.95%	suboptimal
p058	48	12	75.00%	optimal
p059	46	30	34.78%	suboptimal
p060	51	21	58.82%	optimal

Preoperative and postoperative UPDRS-III values were derived from the original clinical table. The continuous UPDRS-III improvement rate and binary response classification followed the finalized analysis dataset. Only de-identified patient IDs are shown.

For Review Only

Supplementary Table 4. Swallow-tail sign analysis.

Variable	Optimal responders, mean ± SD	Suboptimal responders, mean ± SD	Optimal responders, median [IQR]	Suboptimal responders, median [IQR]	Mann–Whitney U	P value	Rank-biserial r	Spearman rho with UPDRS-III improvement	Spearman P	AUC
Left swallow-tail score	0.92 ± 0.28	0.91 ± 0.29	1.00 [1.00, 1.00]	1.00 [1.00, 1.00]	428.00	0.949	0.006	-0.016	0.905	0.503
Right swallow-tail score	0.89 ± 0.31	0.83 ± 0.39	1.00 [1.00, 1.00]	1.00 [1.00, 1.00]	453.50	0.478	0.066	0.014	0.915	0.533
Total swallow-tail score	1.81 ± 0.52	1.74 ± 0.54	2.00 [2.00, 2.00]	2.00 [2.00, 2.00]	458.00	0.453	0.076	0.030	0.817	0.538

Swallow-tail signs were rated on preoperative SWI images. Final ratings were determined by consensus after review by two radiologists. Between-group comparisons were performed using Mann–Whitney U tests. Spearman correlation was calculated against continuous UPDRS-III improvement rates. AUC values indicate discrimination performance for optimal versus suboptimal responders.

Supplementary Table 5. Active contact MNI coordinates in optimal and suboptimal responders.

Variable	Optimal responders, mean ± SD	Optimal responders, median [IQR]	Suboptimal responders, mean ± SD	Suboptimal responders, median [IQR]	Mann–Whitney U	P value	Rank-biserial r	FDR q value
Left active contact MNI x, mm	-12.12 ± 1.20	-11.81 [-12.52, -11.33]	-12.25 ± 1.23	-12.15 [-12.75, -11.60]	473.00	0.475	0.112	0.570
Left active contact MNI y, mm	-12.83 ± 1.53	-12.98 [-14.01, -11.92]	-12.99 ± 1.28	-12.86 [-13.89, -12.16]	435.00	0.891	0.022	0.891
Left active contact MNI z, mm	-7.44 ± 1.57	-7.68 [-8.71, -6.67]	-6.95 ± 1.71	-7.53 [-7.84, -6.27]	371.00	0.412	-0.128	0.570
Right active contact MNI x, mm	12.92 ± 1.24	13.07 [12.33, 13.58]	13.28 ± 1.21	13.06 [12.87, 14.00]	372.00	0.420	-0.126	0.570
Right active contact MNI y, mm	-12.18 ± 1.56	-11.88 [-13.27, -11.01]	-12.71 ± 1.70	-12.89 [-13.82, -12.06]	529.00	0.117	0.243	0.570
Right active contact MNI z, mm	-6.79 ± 1.91	-6.47 [-7.65, -5.86]	-7.13 ± 1.97	-7.49 [-8.73, -5.64]	478.00	0.429	0.123	0.570

Values are summarized as mean ± standard deviation and median [interquartile range]. Between-group comparisons were performed using Mann–Whitney U tests. FDR correction was performed using the Benjamini–Hochberg method.

Supplementary Table 6. Stimulation parameters, VTA metrics, and VTA-STN overlap analyses by outcome group.

Variable	Optimal responders, mean ± SD	Optimal responders, median [IQR]	Suboptimal responders, mean ± SD	Suboptimal responders, median [IQR]	Mann–Whitney U	P value	Rank-biserial r	FDR q value
Total bilateral TEED, μJ/s	114.43 ± 69.23	98.44 [59.15, 134.90]	133.58 ± 65.74	122.03 [86.73, 176.36]	336.00	0.176	-0.210	0.264
Mean bilateral TEED, μJ/s	57.21 ± 34.62	49.22 [29.57, 67.45]	66.79 ± 32.87	61.02 [43.36, 88.18]	336.00	0.176	-0.210	0.264
Total bilateral VTA volume, mm^3	167.39 ± 87.89	142.98 [102.84, 199.01]	221.50 ± 100.62	210.80 [127.77, 322.98]	290.00	0.040	-0.318	0.115
Mean bilateral VTA volume, mm^3	83.70 ± 43.94	71.49 [51.42, 99.51]	110.75 ± 50.31	105.40 [63.88, 161.49]	290.00	0.040	-0.318	0.115
Mean bilateral maximum electric field	4230.27 ± 749.73	4146.75 [3676.83, 4791.46]	4452.20 ± 826.99	4406.62 [3879.08, 5013.00]	352.00	0.267	-0.173	0.356
Mean bilateral electric field within VTA	399.00 ± 12.62	399.06 [392.48, 402.84]	396.96 ± 14.87	397.27 [386.63, 402.73]	468.00	0.523	0.100	0.571
Total bilateral VTA-STN overlap, mm^3	144.28 ± 72.84	131.19 [93.39, 170.67]	190.69 ± 88.32	166.72 [112.52, 283.57]	294.00	0.046	-0.309	0.115
Total bilateral VTA-motor STN overlap, mm^3	32.49 ± 20.65	27.61 [20.33, 43.76]	46.65 ± 21.97	47.13 [26.87, 66.64]	265.00	0.015	-0.377	0.115

Total bilateral VTA-associative STN overlap, mm^3	29.01 ± 20.28	29.80 [11.16, 40.36]	30.47 ± 21.74	21.34 [11.99, 46.04]	412.00	0.843	-0.032	0.843
Total bilateral VTA-limbic STN overlap, mm^3	9.48 ± 8.72	8.01 [3.73, 10.53]	11.43 ± 7.49	12.36 [6.42, 15.42]	328.00	0.140	-0.229	0.264
Mean bilateral VTA inside STN, %	88.61 ± 13.20	94.08 [81.50, 99.45]	87.25 ± 14.92	92.34 [84.58, 96.41]	476.50	0.442	0.120	0.531
Mean bilateral STN coverage by VTA, %	9.84 ± 4.91	8.84 [6.33, 11.76]	12.96 ± 5.93	11.24 [7.66, 19.17]	295.00	0.048	-0.307	0.115

Values are summarized as mean ± standard deviation and median [interquartile range]. Between-group comparisons were performed using Mann–Whitney U tests and interpreted as exploratory analyses. FDR correction was performed using the Benjamini–Hochberg method. TEED, total electrical energy delivered; VTA, volume of tissue activated; STN, subthalamic nucleus.

Supplementary Table 7. Patient-level DBS stimulation parameters, active contact coordinates, VTA metrics, and VTA-STN overlap values.

This table is provided as a separate Excel file: Supplementary_Table_7_patient_level_DBS_metrics_FINAL.xlsx. Only de-identified patient IDs are included.

For Review Only

ID	OutcomeG	UPDRS3_In	L_Contact	R_Contact	L_StimMoc	R_StimMoc	L_Voltage_	R_Voltage_
sub-sub001	optimal	0.816667	C+7-	C+3-	monopolar	monopolar	1.6	1.5
sub-sub002	optimal	0.555556	C+6-	C+3-	monopolar	monopolar	2.5	2.5
sub-sub003	optimal	0.647059	C+6-	C+2-	monopolar	monopolar	2.2	2.5
sub-sub004	optimal	0.6	C+6-	C+3-	monopolar	monopolar	1.7	1.9
sub-sub005	optimal	0.525641	C+6-	C+3-4-	monopolar	monopolar	3	2.5
sub-sub006	suboptimal	0.461538	C+6-7-	C+3-	monopolar	monopolar	1.7	1.7
sub-sub007	suboptimal	0.148936	C+7-	C+3-	monopolar	monopolar	2.6	2.1
sub-sub008	optimal	0.646154	C+8-	C+3-	monopolar	monopolar	2.4	1.5
sub-sub009	optimal	0.695652	7+6-	C+4-	bipolar	monopolar	2.5	2.5
sub-sub010	optimal	0.72973	C+6-	C+3-	monopolar	monopolar	2.2	2.5
sub-sub011	suboptimal	0.421053	C+7-	C+3-	monopolar	monopolar	2	2
sub-sub012	optimal	0.531915	C+6-	C+3-	monopolar	monopolar	2.2	2.75
sub-sub013	suboptimal	0.25	C+6-	C+3-	monopolar	monopolar	2.6	2.6
sub-sub014	suboptimal	0.222222	C+8- C+7-	C+2- C+3- (	monopolar	monopolar	1.866667	3
sub-sub015	suboptimal	0.208333	C+7-	C+2-	monopolar	monopolar	1.8	2.1
sub-sub016	optimal	0.613636	C+6-	C+3-	monopolar	monopolar	2	1.6
sub-sub017	suboptimal	0.285714	C+8- C+7-	C+2-3-	monopolar	monopolar	2.8	3
sub-sub018	suboptimal	0.122449	C+6-7-	C+2-	monopolar	monopolar	2.1	2.9
sub-sub019	optimal	0.534884	C+7-	C+3-	monopolar	monopolar	1.8	2
sub-sub020	optimal	0.511628	C+7-	C+3-	monopolar	monopolar	2.4	2.7
sub-sub021	optimal	0.676471	C+6-8-	C+3-	monopolar	monopolar	2	2.5
sub-sub022	optimal	0.645161	C+7-	C+3-	monopolar	monopolar	1.8	2.5
sub-sub023	optimal	0.533333	C+6-	C+3-	monopolar	monopolar	2.3	2
sub-sub024	suboptimal	0.442857	C+7-	C+4-	monopolar	monopolar	1.7	1.75
sub-sub025	suboptimal	0.45614	C+7-	C+2-3-	monopolar	monopolar	3.5	2.8
sub-sub026	suboptimal	0.392857	C+7-8-	C+2-3+-	monopolar	bipolar	3	3
sub-sub027	optimal	0.769231	C+6-	C+1-	monopolar	monopolar	2.3	1.7
sub-sub028	suboptimal	0.363636	C+7-	C+3-	monopolar	monopolar	2.5	2.5
sub-sub029	suboptimal	0.123288	C+6-	C+2-3-	monopolar	monopolar	2.5	2.2
sub-sub030	optimal	0.6	C+7-	C+3-	monopolar	monopolar	1.8	2
sub-sub031	suboptimal	0.473684	C+7-	C+2-	monopolar	monopolar	3	2.1
sub-sub032	optimal	0.871795	C+7-	C+1-2-	monopolar	monopolar	2.3	2.9
sub-sub033	optimal	0.927536	C+7-	3-4+	monopolar	bipolar	1.7	2.5
sub-sub034	suboptimal	0.4	C+8-	C+3-	monopolar	monopolar	2.2	1.9
sub-sub035	optimal	0.557692	C+7-	C+4-	monopolar	monopolar	2.4	2.4
sub-sub036	optimal	0.724638	C+7-	C+2-	monopolar	monopolar	2.3	2.3
sub-sub037	optimal	0.649123	C+6-	C+3-	monopolar	monopolar	2.3	3.4
sub-sub038	suboptimal	0.481481	C+6-7-	C+2- 3+2-	monopolar	bipolar	2.45	2.8
sub-sub039	optimal	0.882353	C+7-	C+3-	monopolar	monopolar	1.8	2
sub-sub040	suboptimal	0.428571	5-6-	1-2-	noncase_o	noncase_o	2.5	2.5
sub-sub041	optimal	0.529412	C+7-	C+2-3-	monopolar	monopolar	2.15	2
sub-sub042	suboptimal	0.428571	C+6- C+7-	C+2-3-	monopolar	monopolar	2.5	2.65
sub-sub043	optimal	0.71875	C+7-	C+4-	monopolar	monopolar	2.35	2.35
sub-sub044	suboptimal	0.413043	C+7-	C+3-	monopolar	monopolar	2.3	2.1
sub-sub045	optimal	0.74359	C+6-	C+3-4-	monopolar	monopolar	3	3
sub-sub046	optimal	0.808511	C+6-	C+1-	monopolar	monopolar	1.75	1.55
sub-sub047	optimal	0.574468	C+6-	C+2-3-	monopolar	monopolar	2.5	2
sub-sub048	optimal	0.603448	C+7-	C+2-	monopolar	monopolar	2.2	2.2
sub-sub049	optimal	0.661017	C+6-	C+2-	monopolar	monopolar	3.3	3

For Review Only

sub-sub05(	optimal	0.705882	C+6-	C+2-	monopolar	monopolar	2.2	2.1
sub-sub051	optimal	0.921875	C+6-C+8-	C+2-C+1-	monopolar	monopolar	2.75	2.5
sub-sub052	optimal	0.627451	C+6-	C+3-	monopolar	monopolar	2.7	2.7
sub-sub053	optimal	0.666667	C+6-7-	C+2- C+3-	monopolar	monopolar	2.5	2.5
sub-sub054	suboptimal	0.348837	C+6-	C+2-3-	monopolar	monopolar	3.5	2.5
sub-sub055	optimal	0.632653	C+6-7-	C+2-3-	monopolar	monopolar	3	2.5
sub-sub056	suboptimal	0.489362	C+6-7-	C+2-3-	monopolar	monopolar	2.5	2.5
sub-sub057	suboptimal	0.289474	C+6-7-	C+3-	monopolar	monopolar	3	2.8
sub-sub058	optimal	0.75	C+8-	C+2-3-	monopolar	monopolar	3	3
sub-sub059	suboptimal	0.347826	C+6-	C+2-1-	monopolar	monopolar	2.5	1.8
sub-sub06(	optimal	0.588235	C+6-7-	C+3-	monopolar	monopolar	1.5	1.9

For Review Only

L_PulseWic	R_PulseWi	L_Frequenc	R_Frequen	Total_TEED	Mean_LR_	L_ActiveCo	L_ActiveCo	L_ActiveCo
50	50	130	130	31.265	15.6325	-12.3292	-13.4368	-5.46835
60	60	130	130	97.5	48.75	-11.3317	-12.9835	-7.76101
70	60	140	140	99.932	49.966	-15.5113	-13.5335	-5.25652
70	70	130	130	59.15	29.575	-11.6552	-14.7383	-8.33319
80	60	165	165	180.675	90.3375	-11.7324	-14.1189	-9.31515
60	70	150	150	56.355	28.1775	-10.6872	-13.8095	-8.9141
80	60	130	130	104.702	52.351	-13.3224	-11.6049	-6.47064
70	60	150	150	80.73	40.365	-14.6863	-9.53752	-3.9534
70	70	150	150	98.4375	49.21875	-11.327	-13.9957	-9.43266
70	70	150	150	116.445	58.2225	-10.7744	-12.7061	-8.56088
70	70	140	140	78.4	39.2	-11.9922	-12.8748	-7.59746
60	70	140	140	114.7685	57.38425	-11.1609	-13.7287	-8.74277
70	70	150	150	141.96	70.98	-12.1462	-15.5424	-7.85161
56.66667	60	140	140	160.09	80.045	-11.9448	-14.2414	-6.07634
60	70	130	130	65.403	32.7015	-12.9347	-12.1372	-7.75763
60	60	130	130	51.168	25.584	-12.9442	-14.4951	-7.68368
70	70	150	150	176.82	88.41	-12.0727	-13.5812	-3.84232
60	70	150	150	127.995	63.9975	-10.9765	-13.9739	-9.39666
50	60	140	140	56.28	28.14	-11.4061	-12.8852	-7.11519
60	70	140	140	119.826	59.913	-12.2823	-14.3912	-5.72809
60	80	140	140	103.6	51.8	-11.9309	-11.4675	-6.9635
60	60	130	130	74.022	37.011	-12.154	-11.9326	-5.97635
60	60	140	140	78.036	39.018	-12.2325	-14.2186	-10.5526
50	60	130	130	42.6725	21.33625	-13.2267	-12.8886	-5.95441
80	70	140	150	219.52	109.76	-15.7439	-12.577	-3.05681
90	90	180	180	218.7	109.35	-12.3847	-11.5913	-7.45776
60	60	120	120	58.896	29.448	-11.8068	-13.7402	-8.84587
70	70	150	150	131.25	65.625	-12.6141	-12.102	-7.82825
70	70	140	140	108.682	54.341	-11.8127	-14.5983	-9.32435
60	50	140	140	55.216	27.608	-13.4101	-11.4351	-6.16909
70	70	130	130	122.031	61.0155	-12.5743	-12.1875	-7.99578
60	70	130	130	117.793	58.8965	-11.8131	-12.447	-6.94243
60	60	130	130	46.917	23.4585	-12.4555	-12.1149	-5.79003
70	50	130	130	67.509	33.7545	-14.3844	-10.8398	-3.26209
70	70	145	145	116.928	58.464	-14.106	-14.9557	-6.05318
70	80	170	170	134.895	67.4475	-14.4531	-11.3007	-3.72352
60	80	150	150	186.33	93.165	-10.5578	-13.4253	-6.86767
75	70	150	150	175.905	87.9525	-10.6953	-10.8916	-6.49035
60	60	130	130	56.472	28.236	-11.2056	-11.9225	-7.85993
60	60	140	140	52.5	26.25	-11.3888	-12.7767	-6.87287
80	80	170	170	117.266	58.633	-13.9402	-11.6644	-6.8159
70	75	150	150	223.29	111.645	-10.5603	-12.7785	-8.11635
60	70	130	130	93.33025	46.66513	-11.9172	-12.3607	-7.70098
70	70	140	140	95.06	47.53	-12.8936	-14.8412	-5.70472
60	60	130	130	140.4	70.2	-11.3274	-14.9343	-7.89502
60	70	130	130	45.75025	22.87513	-12.612	-14.8443	-8.70842
70	60	130	130	88.075	44.0375	-11.572	-12.2944	-7.29955
60	60	140	140	81.312	40.656	-11.7084	-11.6213	-6.67114
100	90	170	170	322.83	161.415	-12.7716	-14.0133	-7.56164

For Review Only

60	70	130	130	77.883	38.9415	-11.3251	-14.6203	-8.90633
80	65	150	150	317.625	158.8125	-10.0656	-10.5491	-7.71284
70	70	170	170	173.502	86.751	-11.2285	-12.5788	-10.3463
75	80	130	130	186.42	93.21	-12.1743	-10.2867	-8.04514
110	70	160	160	285.6	142.8	-10.6128	-14.9619	-7.5339
70	70	150	150	160.125	80.0625	-11.219	-13.2253	-8.8777
70	70	120	120	105	52.5	-11.9644	-12.8643	-7.73116
80	70	170	170	215.696	107.848	-12.4932	-12.1849	-6.86501
90	80	160	160	244.8	122.4	-10.7998	-8.89833	-6.85637
80	60	140	140	97.216	48.608	-12.214	-13.0162	-7.75603
60	60	140	140	49.224	24.612	-12.5246	-13.3195	-8.89204

For Review Only

R_ActiveCc	R_ActiveCc	R_ActiveCc	Total_VTA_	Mean_LR_\	Mean_LR_I	Mean_LR_I	Total_VTA_	Total_VTA_
12.4933	-13.729	-4.54871	57.96507	28.98253	3195.457	394.9983	57.74063	23.7414
13.3806	-13.0532	-5.82101	165.2279	82.61394	5239.457	392.7594	165.1903	59.60633
11.5186	-15.0444	-7.70303	147.1333	73.56663	4919.77	398.0945	140.675	43.76461
12.5683	-14.0845	-6.79345	74.26256	37.13128	3676.826	399.7731	74.26256	53.16852
13.58295	-10.2729	-6.41141	278.1393	139.0696	4459.969	392.4826	229.4477	40.17293
13.7914	-13.4212	-7.99034	104.7374	52.36871	3125.641	386.7747	104.217	30.6791
12.8606	-12.0239	-6.32107	133.8553	66.92767	4557.378	406.6157	129.55	24.86184
12.661	-11.4978	-3.66053	105.0609	52.53046	3761.031	402.8389	40.64605	0.435627
12.9401	-11.299	-5.85447	131.2292	65.61459	4571.05	435.1636	131.1876	28.85916
12.9668	-10.526	-5.97339	142.9775	71.48875	4117.286	408.4143	141.0165	20.92826
12.9036	-12.5071	-7.48574	101.3401	50.67004	3790.082	401.4749	101.3401	34.87425
14.9676	-10.2866	-6.00983	157.7495	78.87477	5129.926	400.9838	122.0033	26.98036
10.0678	-12.9151	-5.59305	170.7969	85.39844	5314.952	400.5446	160.5753	50.15863
13.40347	-16.0628	-9.28706	322.4926	161.2463	4406.617	400.2808	284.9962	94.67239
12.9246	-13.9907	-9.32084	93.17389	46.58695	3544.749	401.6408	89.39061	19.56647
13.5006	-13.1645	-6.11501	77.65252	38.82626	3256.054	407.6747	77.65252	51.17223
14.2129	-12.8207	-6.96424	355.2498	177.6249	5307.702	415.5683	282.1358	73.37697
13.0083	-14.9509	-8.83877	217.6834	108.8417	4277.237	386.483	199.7188	62.12999
13.7548	-14.2359	-7.33824	98.12002	49.06001	3558.313	399.0594	98.12002	49.42704
12.176	-12.8125	-5.5115	172.0251	86.01254	4586.323	400.7638	170.6715	62.37814
11.5233	-11.0142	-5.86454	199.0129	99.50647	3834.001	375.4362	185.3995	24.73623
13.3389	-11.3519	-5.88963	119.9213	59.96066	3911.044	391.093	119.0299	21.97527
12.8776	-11.8789	-7.44427	118.3905	59.19526	3579.691	402.3478	98.18655	20.32587
15.7075	-9.22138	-4.78285	75.59525	37.79762	2819.595	395.4107	45.0536	17.02736
14.20185	-10.891	-2.86242	376.0323	188.0161	6341.066	388.7551	150.8584	24.98617
12.6818	-12.4462	-9.03946	277.0046	138.5023	5182.909	447.1437	254.172	34.90576
10.6645	-14.1498	-10.4545	102.8426	51.42132	3294.423	401.3	96.93372	29.19349
14.2318	-8.3043	-3.90573	164.7266	82.36328	4944.343	394.8494	106.2581	15.70841
13.45365	-13.9389	-8.61703	210.7966	105.3983	3968.076	381.3985	197.2527	67.72958
12.8536	-10.6737	-5.95222	86.9515	43.47575	3686.883	403.815	86.22255	6.631005
12.8819	-13.8178	-9.74718	176.4753	88.23763	4946.449	404.0974	166.7231	28.75117
11.9135	-13.2665	-9.86084	274.2935	137.1467	4475.008	388.4323	226.2662	47.4961
13.2835	-10.5299	-4.05155	73.50645	36.75323	3897.337	437.5861	51.65154	9.644701
12.9034	-13.4994	-5.48663	104.861	52.43052	4150.031	403.5056	71.06857	23.595
12.3294	-11.3223	-1.5005	146.1417	73.07085	4248.175	399.8922	84.64149	36.1727
11.1065	-13.8512	-6.18245	138.8319	69.41594	4346.317	406.6809	108.6657	23.24084
14.9995	-11.7999	-5.19476	202.8455	101.4228	5232.763	404.0428	167.5446	40.37282
13.0625	-13.8271	-7.99486	277.2983	138.6491	4438.683	404.1483	258.5507	52.55954
11.9633	-11.7133	-8.3478	90.86381	45.4319	3638.082	402.7919	90.86381	10.6485
11.7921	-12.4458	-8.24872	330.0908	165.0454	4633.089	383.626	326.531	67.14368
14.8041	-12.5916	-7.08976	161.0708	80.53541	4146.751	386.6891	140.4521	27.61214
13.32985	-13.164	-8.51351	341.4163	170.7082	4208.555	378.4172	314.6359	67.63367
14.0235	-11.906	-6.46525	139.9185	69.95924	4583.657	398.7193	138.7125	32.30703
12.1946	-12.8862	-4.63312	121.684	60.84198	4230.373	401.9503	118.7844	47.1283
13.8023	-13.5644	-6.20617	325.9526	162.9763	4791.456	396.5305	316.0848	100.7234
9.15859	-16.2999	-10.8941	75.48823	37.74411	3198.306	390.0105	53.41648	22.73979
13.56695	-10.6672	-7.10558	176.2085	88.10425	4159.34	388.0532	165.1379	25.85096
13.084	-14.0744	-7.79986	119.6909	59.84545	4132.25	395.0759	119.6909	42.07213
13.0664	-11.119	-7.60882	253.7342	126.8671	5979.372	400.5949	247.3764	67.88532

For Review Only

13.2119	-12.6702	-7.56377	110.1795	55.08973	4022.124	410.7379	109.6007	42.91954
13.07345	-12.3175	-10.1946	313.8293	156.9147	5576.576	413.4816	234.6947	7.445686
14.5871	-11.3351	-7.17857	188.8277	94.41384	5176.605	397.2252	149.4762	17.51073
13.9113	-9.31121	-5.29754	343.0821	171.5411	3563.502	378.5719	229.2227	7.298217
12.21915	-11.4694	-6.66999	307.3687	153.6844	5635.414	397.2704	296.6084	66.12735
11.0791	-11.8768	-9.03766	385.7329	192.8665	5055.195	385.0531	353.0975	50.13586
14.79	-12.1016	-6.37199	323.4584	161.7292	3774.743	380.2051	289.0352	70.64696
15.5282	-11.6065	-5.69119	336.227	168.1135	5079.542	389.9684	285.9234	57.84141
14.0866	-10.5702	-7.6495	337.1594	168.5797	4838.696	398.1217	223.9848	11.59978
13.38535	-13.9364	-9.69725	172.2342	86.11708	3723.38	380.0354	152.5046	40.86237
13.3427	-10.7335	-8.77604	101.5278	50.76391	2681.062	377.8542	93.38908	15.0488

For Review Only

Total_VTA_	Total_VTA_	Mean_LR_\	Mean_LR_STN_Coverage_pct_resliced
8.077242	0.2237	99.59544	3.888276
49.15302	8.009868	99.97688	11.1763
4.550184	9.857149	94.52193	9.688398
5.275595	3.044481	100	5.051223
39.36487	13.1531	83.55815	15.47969
20.01642	6.766442	99.63933	7.159506
37.0593	9.094247	97.40695	8.881501
4.895877	0.090232	52.18788	2.894742
43.00596	6.5529	99.9756	8.838246
54.71966	7.859341	98.77242	9.490514
27.55517	12.36007	100	6.870641
24.12345	3.645394	81.49935	8.537351
20.1988	2.92314	93.88371	10.96178
9.695775	6.085877	92.19921	19.24223
8.430801	12.54683	96.48637	6.065573
5.278625	4.531115	100	5.303877
23.00849	9.374101	77.66715	19.09381
20.49427	12.62822	91.7655	13.44616
22.40309	6.001891	100	6.658982
33.09282	2.92309	99.28753	11.49967
68.62003	8.713844	94.07549	12.48174
37.64742	5.124708	99.44929	8.087492
14.86958	8.591886	85.02244	6.652089
6.766585	3.137863	61.76277	3.302452
11.91103	2.079213	39.83778	10.57551
55.40949	17.01541	94.92215	17.24884
11.16188	9.936678	92.26159	6.541546
21.34217	6.759621	64.1481	7.336653
11.35671	14.44311	93.74687	13.28663
29.79774	3.731453	99.14683	5.89973
27.40127	15.79151	92.33509	11.23654
46.16618	28.60283	88.24318	15.19782
15.83009	1.947389	73.05494	3.551119
9.091466	0.42828	73.03624	5.034407
0.004947	4.171811	58.85624	6.002726
15.36906	0.920433	78.63975	7.475645
29.83705	0.664372	86.90231	11.75941
62.2419	18.4739	94.43678	17.46641
39.14568	17.51718	100	6.127766
85.84505	30.09547	98.92508	21.9401
10.94645	7.828295	87.97078	10.0814
60.08912	25.64629	92.24727	21.1586
40.15903	9.16461	99.13973	9.436814
12.06421	2.332971	97.2507	7.992918
27.20539	11.3626	97.63482	21.27653
0.029218	2.967693	68.02729	3.680697
50.69538	12.37316	94.34944	11.27151
32.50774	10.53003	100	8.130576
40.35761	18.42734	97.27014	16.56057

For Review Only

13.18307	8.824329	99.50672	7.41666
45.68044	31.27241	73.42773	15.86517
23.24946	7.809425	79.15926	10.40055
56.39041	8.448675	66.40307	15.90453
57.68562	17.49213	96.32868	19.90221
83.908	41.10208	91.73371	23.7888
48.45338	12.92634	89.46276	19.59169
43.63288	9.366391	80.34477	19.99492
39.82561	15.99489	68.38879	15.64215
21.0351	15.04583	88.81347	10.31564
6.817855	8.915229	90.60698	6.326586

For Review Only
