# Supplementary material for: Development and application of a multi-task fusion model using susceptibility-weighted imaging-based substantia nigra and adjacent structures for prognostic prediction of subthalamic nucleus deep brain stimulation in Parkinson’s disease
Source: Brain Commun. 2026 Sep 11;8(5):fcag333. doi: 10.1093/braincomms/fcag333 (PMC13563298; doi:10.1093/braincomms/fcag333)
Supplement: Supplementary file 1 [file fcag333_supplementary_data.zip › Supplementary_material.docx]

**Supplementary Material**

Development and application of a multi-task fusion model using susceptibility-weighted imaging-based substantia nigra and adjacent structures for prognostic prediction of subthalamic nucleus deep brain stimulation in Parkinson’s disease

Abbreviations: AUC, area under the receiver operating characteristic curve; BN, batch normalization; CT, computed tomography; DBS, deep brain stimulation; FDR, false discovery rate; HAMA, Hamilton Anxiety Rating Scale; HAMD, Hamilton Depression Rating Scale; LCT, levodopa challenge test; LEDD, levodopa equivalent daily dose; MNI, Montreal Neurological Institute; MoCA, Montreal Cognitive Assessment; MRI, magnetic resonance imaging; NMSS, Non-Motor Symptoms Scale; PDQ-39, 39-item Parkinson’s Disease Questionnaire; PDSS, Parkinson’s Disease Sleep Scale; SNAS, substantia nigra and adjacent structures; STN, subthalamic nucleus; SWI, susceptibility-weighted imaging; TEED, total electrical energy delivered; UPDRS-III, Unified Parkinson’s Disease Rating Scale Part III; VTA, volume of tissue activated.

**Supplementary Figures**

**Supplementary Figure 1. Architecture of the 3D ResNet50 baseline model.**

Legend: The 3D susceptibility-weighted imaging (SWI) sub-volume was processed by an initial convolutional block, four Bottleneck3D residual stages, global average pooling, and a linear classification head to predict optimal versus suboptimal motor response. BN indicates batch normalization.

Alt text: Diagram showing the three-dimensional ResNet50 baseline model, including an initial convolutional block, four residual stages, global average pooling, and a linear classification head.

**Supplementary Figure 2. Representative illustration of SNAS-centered sub-volume extraction from preoperative SWI.**

Legend: (A) Whole axial susceptibility-weighted imaging (SWI) image showing the anatomical location of the selected substantia nigra and adjacent structures (SNAS)-centered region. (B) Magnified axial view showing that the selected region covered the bilateral substantia nigra and adjacent structures. (C, D) Coronal and sagittal views illustrating the three-dimensional spatial extent of the extracted sub-volume.

The red box denotes the fixed-size SNAS-centered sub-volume manually localized on 3D SWI images, with dimensions of 10 × 40 × 80 voxels. The extracted region covered the substantia nigra and adjacent deep brain structures, including part of the subthalamic nucleus (STN) region.

Alt text: Axial, coronal, and sagittal susceptibility-weighted images showing extraction of a fixed-size three-dimensional sub-volume centered on the bilateral substantia nigra and adjacent structures.

**Supplementary Figure 3. Group-level Lead-DBS reconstruction of bilateral STN-DBS electrodes and active contacts.**

Legend: Postoperative computed tomography (CT) images were co-registered with preoperative magnetic resonance imaging (MRI), and electrode trajectories and active contacts for subthalamic nucleus deep brain stimulation (STN-DBS) were reconstructed using Lead-DBS. Electrode trajectories from optimal responders are shown in cyan, whereas those from suboptimal responders are shown in magenta. The clinically active contacts are highlighted on each electrode. Colored surfaces represent atlas-defined STN functional subregions and adjacent deep brain structures to illustrate the anatomical relationship between electrode locations and the STN region. Quantitative active contact coordinates, stimulation parameters, volume of tissue activated (VTA) metrics, and VTA-STN overlap analyses are provided in Supplementary Tables 5–7.

Alt text: Group-level reconstruction showing bilateral deep brain stimulation electrode trajectories and active contacts relative to the subthalamic nucleus and adjacent functional regions. Optimal responders are shown in cyan and suboptimal responders in magenta.

**Supplementary Tables**

**Supplementary Table 1. Detailed demographic and baseline clinical characteristics.**

| **Variable** | **Optimal responders, mean ± SD** | **Suboptimal responders, mean ± SD** | **Optimal responders, median [IQR]** | **Suboptimal responders, median [IQR]** | **Test** | **Statistic** | **P value** | **Rank-biserial r** |
| --- | --- | --- | --- | --- | --- | --- | --- | --- |
| Age, years | 56.19 ± 6.51 | 60.00 ± 6.66 | 56.00 [52.00, 61.00] | 59.00 [55.00, 65.00] | Mann–Whitney U | 307.00 | 0.072 | -0.278 |
| Sex, male/female | 22/15 | 11/12 |  |  | Fisher exact |  | 0.431 |  |
| Disease duration, years | 8.38 ± 2.91 | 8.91 ± 3.92 | 9.00 [6.00, 10.00] | 8.00 [6.00, 10.00] | Mann–Whitney U | 436.00 | 0.878 | 0.025 |
| Age at onset, years | 48.62 ± 7.90 | 51.09 ± 7.12 | 48.00 [44.00, 53.00] | 51.00 [46.00, 54.50] | Mann–Whitney U | 334.00 | 0.166 | -0.215 |
| Hoehn-Yahr stage | 3.31 ± 0.73 | 3.37 ± 0.68 | 3.00 [3.00, 4.00] | 3.00 [3.00, 4.00] | Mann–Whitney U | 389.00 | 0.545 | -0.086 |
| LEDD, mg | 714.86 ± 383.90 | 646.20 ± 241.43 | 650.00 [537.50, 800.00] | 675.00 [450.00, 837.50] | Mann–Whitney U | 440.50 | 0.825 | 0.035 |
| LCT improvement rate | 0.57 ± 0.13 | 0.52 ± 0.14 | 0.58 [0.49, 0.67] | 0.52 [0.42, 0.61] | Mann–Whitney U | 521.50 | 0.146 | 0.226 |
| UPDRS-III | 54.38 ± 12.67 | 51.78 ± 10.86 | 52.00 [47.00, 64.00] | 49.00 [45.50, 57.00] | Mann–Whitney U | 479.00 | 0.420 | 0.126 |
| UPDRS-IV | 6.03 ± 1.77 | 6.30 ± 1.43 | 6.00 [5.00, 7.00] | 6.00 [5.50, 7.00] | Mann–Whitney U | 386.50 | 0.548 | -0.092 |
| NMSS | 72.19 ± 35.91 | 76.30 ± 28.37 | 62.00 [48.00, 95.00] | 70.00 [55.00, 93.50] | Mann–Whitney U | 374.50 | 0.442 | -0.120 |
| PDQ-39 | 62.30 ± 19.06 | 67.09 ± 19.11 | 58.00 [49.00, 76.00] | 63.00 [56.00, 80.00] | Mann–Whitney U | 367.00 | 0.378 | -0.137 |
| MMSE | 26.57 ± 2.72 | 24.78 ± 4.26 | 27.00 [25.00, 29.00] | 26.00 [21.00, 28.00] | Mann–Whitney U | 516.00 | 0.168 | 0.213 |
| MoCA | 20.92 ± 5.23 | 20.09 ± 6.22 | 22.00 [18.00, 25.00] | 20.00 [16.00, 25.00] | Mann–Whitney U | 461.00 | 0.594 | 0.083 |
| HAMD | 12.81 ± 5.83 | 12.70 ± 5.25 | 13.00 [9.00, 17.00] | 13.00 [8.00, 16.00] | Mann–Whitney U | 432.00 | 0.927 | 0.015 |
| HAMA | 14.24 ± 5.85 | 17.70 ± 6.57 | 15.00 [10.00, 18.00] | 17.00 [12.00, 23.00] | Mann–Whitney U | 305.50 | 0.069 | -0.282 |
| PDSS | 99.46 ± 24.56 | 99.00 ± 19.54 | 100.00 [81.00, 120.00] | 98.00 [90.00, 114.00] | Mann–Whitney U | 444.50 | 0.778 | 0.045 |
| Constipation score | 10.22 ± 6.34 | 11.61 ± 5.15 | 9.00 [6.00, 14.00] | 13.00 [8.50, 14.50] | Mann–Whitney U | 347.00 | 0.235 | -0.184 |

*Clinical baseline variables including age, sex, disease duration, age at onset, LEDD, Hoehn-Yahr stage, preoperative UPDRS-III, and LCT improvement rate were derived from the original 60-patient clinical table after manual correction of a known age entry error for one patient. Group classification was based on the finalized outcome definition in the analysis-ready dataset.*

**Supplementary Table 2. Additional clinical outcomes beyond UPDRS-III.**

| **Outcome** | **Direction of favorable change** | **Preoperative optimal** | **Preoperative suboptimal** | **Preoperative P** | **Postoperative optimal** | **Postoperative suboptimal** | **Postoperative P** | **Favorable change optimal** | **Favorable change suboptimal** | **Change P** | **Change FDR q** | **Favorable change, % optimal** | **Favorable change, % suboptimal** | **Percentage change P** | **Percentage change FDR q** |
| --- | --- | --- | --- | --- | --- | --- | --- | --- | --- | --- | --- | --- | --- | --- | --- |
| LEDD, mg | Pre − post | 714.86 ± 383.90 | 646.20 ± 241.43 | 0.825 | 483.50 ± 253.09 | 409.74 ± 214.18 | 0.283 | 231.36 ± 368.14 | 236.46 ± 277.59 | 0.704 | 0.873 | 27.69 ± 38.45 | 27.51 ± 50.48 | 0.654 | 0.921 |
| UPDRS-IV | Pre − post | 6.03 ± 1.77 | 6.30 ± 1.43 | 0.548 | 4.22 ± 2.48 | 5.00 ± 1.48 | 0.174 | 1.81 ± 3.41 | 1.30 ± 1.96 | 0.736 | 0.873 | 20.70 ± 53.87 | 17.24 ± 30.61 | 0.546 | 0.910 |
| NMSS | Pre − post | 72.19 ± 35.91 | 76.30 ± 28.37 | 0.442 | 51.14 ± 31.64 | 62.22 ± 33.46 | 0.204 | 21.05 ± 39.38 | 14.09 ± 33.57 | 0.687 | 0.873 | -38.64 ± 368.49 | 13.18 ± 41.78 | 0.543 | 0.910 |
| PDQ-39 | Pre − post | 62.30 ± 19.06 | 67.09 ± 19.11 | 0.378 | 37.95 ± 22.30 | 56.13 ± 20.30 | 0.005 | 24.35 ± 21.58 | 10.96 ± 19.05 | 0.011 | 0.106 | 39.19 ± 31.09 | 13.75 ± 28.05 | 0.003 | 0.031 |
| MMSE | Post − pre | 26.57 ± 2.72 | 24.78 ± 4.26 | 0.168 | 26.78 ± 3.37 | 24.78 ± 5.15 | 0.180 | 0.22 ± 2.37 | 0.00 ± 4.08 | 0.800 | 0.873 | 0.91 ± 8.98 | 0.94 ± 19.57 | 0.921 | 0.921 |
| MoCA | Post − pre | 20.92 ± 5.23 | 20.09 ± 6.22 | 0.594 | 23.16 ± 4.94 | 20.83 ± 5.57 | 0.085 | 2.24 ± 3.45 | 0.74 ± 4.01 | 0.136 | 0.680 | 13.88 ± 20.97 | 8.33 ± 29.97 | 0.138 | 0.689 |
| HAMD | Pre − post | 12.81 ± 5.83 | 12.70 ± 5.25 | 0.927 | 8.97 ± 6.03 | 11.61 ± 6.24 | 0.107 | 3.84 ± 8.20 | 1.09 ± 7.17 | 0.241 | 0.804 | 12.22 ± 68.36 | -0.07 ± 59.47 | 0.338 | 0.910 |
| HAMA | Pre − post | 14.24 ± 5.85 | 17.70 ± 6.57 | 0.069 | 8.92 ± 6.05 | 12.26 ± 7.29 | 0.076 | 5.32 ± 8.71 | 5.43 ± 8.73 | 0.873 | 0.873 | 16.14 ± 77.99 | 22.19 ± 54.52 | 0.861 | 0.921 |
| PDSS | Post − pre | 99.46 ± 24.56 | 99.00 ± 19.54 | 0.778 | 114.84 ± 24.59 | 110.17 ± 24.50 | 0.204 | 15.38 ± 32.34 | 11.17 ± 20.75 | 0.461 | 0.873 | 23.36 ± 41.44 | 13.19 ± 28.16 | 0.494 | 0.910 |
| Constipation score | Pre − post | 10.22 ± 6.34 | 11.61 ± 5.15 | 0.235 | 8.83 ± 7.93 | 9.70 ± 6.14 | 0.334 | 1.39 ± 8.64 | 1.91 ± 5.26 | 0.703 | 0.873 | -6.47 ± 88.05 | 12.22 ± 56.12 | 0.738 | 0.921 |

*LEDD baseline values were derived from the original clinical table, while postoperative and additional scale variables were obtained from the finalized merged dataset. For scales in which lower scores indicate better status, favorable change was defined as preoperative score minus postoperative score. For scales in which higher scores indicate better status, favorable change was defined as postoperative score minus preoperative score. P values were calculated using Mann–Whitney U tests. FDR correction was performed using the Benjamini–Hochberg method.*

**Supplementary Table 3. Individual preoperative and postoperative UPDRS-III scores and continuous improvement rates.**

| **ID** | **Preoperative UPDRS-III** | **1-year postoperative stimulation-on/medication-off UPDRS-III** | **UPDRS-III improvement rate** | **Response classification** |
| --- | --- | --- | --- | --- |
| p001 | 60 | 11 | 81.67% | optimal |
| p002 | 63 | 28 | 55.56% | optimal |
| p003 | 51 | 18 | 64.71% | optimal |
| p004 | 55 | 22 | 60.00% | optimal |
| p005 | 78 | 37 | 52.56% | optimal |
| p006 | 65 | 35 | 46.15% | suboptimal |
| p007 | 47 | 40 | 14.89% | suboptimal |
| p008 | 65 | 23 | 64.62% | optimal |
| p009 | 69 | 21 | 69.57% | optimal |
| p010 | 37 | 10 | 72.97% | optimal |
| p011 | 57 | 33 | 42.11% | suboptimal |
| p012 | 47 | 22 | 53.19% | optimal |
| p013 | 52 | 39 | 25.00% | suboptimal |
| p014 | 63 | 49 | 22.22% | suboptimal |
| p015 | 48 | 38 | 20.83% | suboptimal |
| p016 | 44 | 17 | 61.36% | optimal |
| p017 | 42 | 30 | 28.57% | suboptimal |
| p018 | 49 | 43 | 12.24% | suboptimal |
| p019 | 43 | 20 | 53.49% | optimal |
| p020 | 43 | 21 | 51.16% | optimal |
| p021 | 34 | 11 | 67.65% | optimal |
| p022 | 62 | 22 | 64.52% | optimal |
| p023 | 75 | 35 | 53.33% | optimal |
| p024 | 70 | 39 | 44.29% | suboptimal |
| p025 | 57 | 31 | 45.61% | suboptimal |
| p026 | 56 | 34 | 39.29% | suboptimal |
| p027 | 78 | 18 | 76.92% | optimal |
| p028 | 66 | 42 | 36.36% | suboptimal |
| p029 | 73 | 64 | 12.33% | suboptimal |
| p030 | 70 | 28 | 60.00% | optimal |
| p031 | 57 | 30 | 47.37% | suboptimal |
| p032 | 39 | 5 | 87.18% | optimal |
| p033 | 69 | 5 | 92.75% | optimal |
| p034 | 45 | 27 | 40.00% | suboptimal |
| p035 | 52 | 23 | 55.77% | optimal |
| p036 | 69 | 19 | 72.46% | optimal |
| p037 | 57 | 20 | 64.91% | optimal |
| p038 | 54 | 28 | 48.15% | suboptimal |
| p039 | 34 | 4 | 88.24% | optimal |
| p040 | 42 | 24 | 42.86% | suboptimal |
| p041 | 51 | 24 | 52.94% | optimal |
| p042 | 28 | 16 | 42.86% | suboptimal |
| p043 | 32 | 9 | 71.88% | optimal |
| p044 | 46 | 27 | 41.30% | suboptimal |
| p045 | 39 | 10 | 74.36% | optimal |
| p046 | 47 | 9 | 80.85% | optimal |
| p047 | 47 | 20 | 57.45% | optimal |
| p048 | 58 | 23 | 60.34% | optimal |
| p049 | 59 | 20 | 66.10% | optimal |
| p050 | 68 | 20 | 70.59% | optimal |
| p051 | 64 | 5 | 92.19% | optimal |
| p052 | 51 | 19 | 62.75% | optimal |
| p053 | 54 | 18 | 66.67% | optimal |
| p054 | 43 | 28 | 34.88% | suboptimal |
| p055 | 49 | 18 | 63.27% | optimal |
| p056 | 47 | 24 | 48.94% | suboptimal |
| p057 | 38 | 27 | 28.95% | suboptimal |
| p058 | 48 | 12 | 75.00% | optimal |
| p059 | 46 | 30 | 34.78% | suboptimal |
| p060 | 51 | 21 | 58.82% | optimal |

*Preoperative and postoperative UPDRS-III values were derived from the original clinical table. The continuous UPDRS-III improvement rate and binary response classification followed the finalized analysis dataset. Only de-identified patient IDs are shown.*

**Supplementary Table 4. Swallow-tail sign analysis.**

| **Variable** | **Optimal responders, mean ± SD** | **Suboptimal responders, mean ± SD** | **Optimal responders, median [IQR]** | **Suboptimal responders, median [IQR]** | **Mann–Whitney U** | **P value** | **Rank-biserial r** | **Spearman rho with UPDRS-III improvement** | **Spearman P** | **AUC** |
| --- | --- | --- | --- | --- | --- | --- | --- | --- | --- | --- |
| Left swallow-tail score | 0.92 ± 0.28 | 0.91 ± 0.29 | 1.00 [1.00, 1.00] | 1.00 [1.00, 1.00] | 428.00 | 0.949 | 0.006 | -0.016 | 0.905 | 0.503 |
| Right swallow-tail score | 0.89 ± 0.31 | 0.83 ± 0.39 | 1.00 [1.00, 1.00] | 1.00 [1.00, 1.00] | 453.50 | 0.478 | 0.066 | 0.014 | 0.915 | 0.533 |
| Total swallow-tail score | 1.81 ± 0.52 | 1.74 ± 0.54 | 2.00 [2.00, 2.00] | 2.00 [2.00, 2.00] | 458.00 | 0.453 | 0.076 | 0.030 | 0.817 | 0.538 |

*Swallow-tail signs were rated on preoperative SWI images. Final ratings were determined by consensus after review by two radiologists. Between-group comparisons were performed using Mann–Whitney U tests. Spearman correlation was calculated against continuous UPDRS-III improvement rates. AUC values indicate discrimination performance for optimal versus suboptimal responders.*

**Supplementary Table 5. Active contact MNI coordinates in optimal and suboptimal responders.**

| **Variable** | **Optimal responders, mean ± SD** | **Optimal responders, median [IQR]** | **Suboptimal responders, mean ± SD** | **Suboptimal responders, median [IQR]** | **Mann–Whitney U** | **P value** | **Rank-biserial r** | **FDR q value** |
| --- | --- | --- | --- | --- | --- | --- | --- | --- |
| Left active contact MNI x, mm | -12.12 ± 1.20 | -11.81 [-12.52, -11.33] | -12.25 ± 1.23 | -12.15 [-12.75, -11.60] | 473.00 | 0.475 | 0.112 | 0.570 |
| Left active contact MNI y, mm | -12.83 ± 1.53 | -12.98 [-14.01, -11.92] | -12.99 ± 1.28 | -12.86 [-13.89, -12.16] | 435.00 | 0.891 | 0.022 | 0.891 |
| Left active contact MNI z, mm | -7.44 ± 1.57 | -7.68 [-8.71, -6.67] | -6.95 ± 1.71 | -7.53 [-7.84, -6.27] | 371.00 | 0.412 | -0.128 | 0.570 |
| Right active contact MNI x, mm | 12.92 ± 1.24 | 13.07 [12.33, 13.58] | 13.28 ± 1.21 | 13.06 [12.87, 14.00] | 372.00 | 0.420 | -0.126 | 0.570 |
| Right active contact MNI y, mm | -12.18 ± 1.56 | -11.88 [-13.27, -11.01] | -12.71 ± 1.70 | -12.89 [-13.82, -12.06] | 529.00 | 0.117 | 0.243 | 0.570 |
| Right active contact MNI z, mm | -6.79 ± 1.91 | -6.47 [-7.65, -5.86] | -7.13 ± 1.97 | -7.49 [-8.73, -5.64] | 478.00 | 0.429 | 0.123 | 0.570 |

*Values are summarized as mean ± standard deviation and median [interquartile range]. Between-group comparisons were performed using Mann–Whitney U tests. FDR correction was performed using the Benjamini–Hochberg method.*

**Supplementary Table 6. Stimulation parameters, VTA metrics, and VTA-STN overlap analyses by outcome group.**

| **Variable** | **Optimal responders, mean ± SD** | **Optimal responders, median [IQR]** | **Suboptimal responders, mean ± SD** | **Suboptimal responders, median [IQR]** | **Mann–Whitney U** | **P value** | **Rank-biserial r** | **FDR q value** |
| --- | --- | --- | --- | --- | --- | --- | --- | --- |
| Total bilateral TEED, μJ/s | 114.43 ± 69.23 | 98.44 [59.15, 134.90] | 133.58 ± 65.74 | 122.03 [86.73, 176.36] | 336.00 | 0.176 | -0.210 | 0.264 |
| Mean bilateral TEED, μJ/s | 57.21 ± 34.62 | 49.22 [29.57, 67.45] | 66.79 ± 32.87 | 61.02 [43.36, 88.18] | 336.00 | 0.176 | -0.210 | 0.264 |
| Total bilateral VTA volume, mm³ | 167.39 ± 87.89 | 142.98 [102.84, 199.01] | 221.50 ± 100.62 | 210.80 [127.77, 322.98] | 290.00 | 0.040 | -0.318 | 0.115 |
| Mean bilateral VTA volume, mm³ | 83.70 ± 43.94 | 71.49 [51.42, 99.51] | 110.75 ± 50.31 | 105.40 [63.88, 161.49] | 290.00 | 0.040 | -0.318 | 0.115 |
| Mean bilateral maximum electric field | 4230.27 ± 749.73 | 4146.75 [3676.83, 4791.46] | 4452.20 ± 826.99 | 4406.62 [3879.08, 5013.00] | 352.00 | 0.267 | -0.173 | 0.356 |
| Mean bilateral electric field within VTA | 399.00 ± 12.62 | 399.06 [392.48, 402.84] | 396.96 ± 14.87 | 397.27 [386.63, 402.73] | 468.00 | 0.523 | 0.100 | 0.571 |
| Total bilateral VTA-STN overlap, mm³ | 144.28 ± 72.84 | 131.19 [93.39, 170.67] | 190.69 ± 88.32 | 166.72 [112.52, 283.57] | 294.00 | 0.046 | -0.309 | 0.115 |
| Total bilateral VTA-motor STN overlap, mm³ | 32.49 ± 20.65 | 27.61 [20.33, 43.76] | 46.65 ± 21.97 | 47.13 [26.87, 66.64] | 265.00 | 0.015 | -0.377 | 0.115 |
| Total bilateral VTA-associative STN overlap, mm³ | 29.01 ± 20.28 | 29.80 [11.16, 40.36] | 30.47 ± 21.74 | 21.34 [11.99, 46.04] | 412.00 | 0.843 | -0.032 | 0.843 |
| Total bilateral VTA-limbic STN overlap, mm³ | 9.48 ± 8.72 | 8.01 [3.73, 10.53] | 11.43 ± 7.49 | 12.36 [6.42, 15.42] | 328.00 | 0.140 | -0.229 | 0.264 |
| Mean bilateral VTA inside STN, % | 88.61 ± 13.20 | 94.08 [81.50, 99.45] | 87.25 ± 14.92 | 92.34 [84.58, 96.41] | 476.50 | 0.442 | 0.120 | 0.531 |
| Mean bilateral STN coverage by VTA, % | 9.84 ± 4.91 | 8.84 [6.33, 11.76] | 12.96 ± 5.93 | 11.24 [7.66, 19.17] | 295.00 | 0.048 | -0.307 | 0.115 |

*Values are summarized as mean ± standard deviation and median [interquartile range]. Between-group comparisons were performed using Mann–Whitney U tests and interpreted as exploratory analyses. FDR correction was performed using the Benjamini–Hochberg method. TEED, total electrical energy delivered; VTA, volume of tissue activated; STN, subthalamic nucleus.*

**Supplementary Table 7. Patient-level DBS stimulation parameters, active contact coordinates, VTA metrics, and VTA-STN overlap values.**

*This table is provided as a separate Excel file: Supplementary_Table_7_patient_level_DBS_metrics_FINAL.xlsx. Only de-identified patient IDs are included.*
